# A cell abundance analysis based on efficient PAM clustering for a better understanding of the dynamics of endometrial remodelling

**DOI:** 10.1186/s12859-023-05569-6

**Published:** 2023-11-22

**Authors:** Juan Domingo, Oleksandra Kutsyr-Kolesnyk, Teresa Leon, Raul Perez-Moraga, Guillermo Ayala, Beatriz Roson

**Affiliations:** 1 Department of Informatics, ETSE, University of Valencia, Avda. de la Universidad, s/n, 46100 Burjasot, Valencia Spain; 2 Department of Statistics and Operations Research, University of Valencia, Avda. Vicente Andres Estelles, 46100 Burjasot, Valencia Spain; 3 Carlos Simon Foundation, INCLIVA Health Research Institute, Eduardo Primo Yufera, 46012 Valencia, Valencia Spain; 4 Igenomix R&D, Technology Park, 46980 Paterna, Valencia Spain

**Keywords:** Single-cell RNA-seq, Partitioning around medoids, Differential abundance testing, Human endometrial cell types

## Abstract

**Background:**

Single-cell RNA sequencing (scRNA-seq) is a powerful tool for investigating cell abundance changes during tissue regeneration and remodeling processes. Differential cell abundance supports the initial clustering of all cells; then, the number of cells per cluster and sample are evaluated, and the dependence of these counts concerning the phenotypic covariates of the samples is studied. Analysis heavily depends on the clustering method. Partitioning Around Medoids (PAM or k-medoids) represents a well-established clustering procedure that leverages the downstream interpretation of clusters by pinpointing real individuals in the dataset as cluster centers (medoids) without reducing dimensions. Of note, PAM suffers from high computational costs and memory requirements.

**Results:**

This paper proposes a method for differential abundance analysis using PAM as a clustering method and negative binomial regression as a statistical model to relate covariates to cluster/cell counts. We used this approach to study the differential cell abundance of human endometrial cell types throughout the natural secretory phase of the menstrual cycle. We developed a new R package *-scellpam-*, that incorporates an efficient parallel C++ implementation of PAM, and applied this package in this study. We compared the PAM-BS clustering method with other methods and evaluated both the computational aspects of its implementation and the quality of the classifications obtained using distinct published datasets with known subpopulations that demonstrate promising results.

**Conclusions:**

The implementation of PAM-BS, included in the *scellpam* package, exhibits robust performance in terms of speed and memory usage compared to other related methods. PAM allowed quick and robust clustering of sets of cells with a size ranging from 70,000 to 300,000 cells. https://cran.r-project.org/web/packages/scellpam/index.html. Finally, our approach provides important new insights into the transient subpopulations associated with the fertile time frame when applied to the study of changes in the human endometrium during the secretory phase of the menstrual cycle.

**Supplementary Information:**

The online version contains supplementary material available at 10.1186/s12859-023-05569-6.

## Background

Single-cell transcriptomic maps have provided unprecedented insights into the identity of the cellular components of a given tissue. Each cell is described using a high dimensional vector, providing the number of short reads aligned over unitary regions (genes) of a reference genome. As a result, we obtain a high-dimensional transcriptomic description of each cell. Each biological condition (described with phenotypic covariates) provides one or several samples containing thousands of cells, and generally, we consider a selected set of phenotypic variables describing the distribution of samples. A central biological question involves detecting changes in cell populations with regard to their phenotypic description.

Differential abundance analyses comprise three steps: Step one employs a cluster analysis for all cells without considering their biological condition (phenotypic variation) to define (possible) cell types/states; Step two calculates the number of cells per sample and cluster, which yields a count per cluster for each sample, with each sample having associated phenotypic covariates; Step three consists of studying how these counts depend on said phenotypic covariates, revealing the population changes in the area of interest.

The classification of sequenced cells into functional groups (clusters) determines the populations and states constituting the tissue under study [1]. Cell population abundances can become altered under pathological conditions [2, 3]. Evaluating alterations in population abundances under varying conditions using single-cell RNA sequencing (scRNA-seq) data requires thoughtful consideration of every step of the analytical workflow: quality control filters, clustering, and statistical hypothesis testing of differential abundance [4, 5].

This paper proposes the use of partitioning around medoids (PAM) [6] as the clustering procedure for the first step and a negative binomial regression for the third step [7, 8].

A range of clustering methods have been proposed and used for single-cell analysis. Duó et al. [9] systematically evaluated the performance of 14 clustering algorithms implemented in R [10]. The authors “found substantial differences in the performance, run time and stability between the methods with SC3 and Seurat showing the most favorable results”.  Single-Cell Consensus Clustering (SC3) is a tool for unsupervised clustering of scRNA-seq data [11], and Seurat [12–15] is a widely used toolkit for single-cell genomics.

As Luecken et al. [16] highlight in their tutorial on best practices in scRNA-seq analysis, the default clustering algorithm implemented both in Seurat and Scanpy (Single-Cell Analysis in Python) [17] is the Louvain community detection algorithm [18], a hierarchical clustering method initially developed to extract the community structure of large networks. In addition, the authors recommend the use of this algorithm. In contrast, Traag et al. [19] reported that the Louvain algorithm may yield arbitrary and poorly connected communities; instead, they introduced the Leiden algorithm to overcome associated issues. We emphasize that no single method exists that works optimally in all cases; clustering success and method choice should be (at least partially) evaluated through the biological meaning of the cell groups obtained.

As proposed in [6] -chapter 2, PAM represents a robust example of an intuitive clustering method that minimizes a well-defined reasonable objective function: the sum of distances to the nearest cluster center.

The original algorithm was later improved with faster versions (FASTPAM1 with and without eager swapping [20]), which has been evaluated using several benchmark problems and displays robustness and interpretability. Section 2.2 details the clustering algorithm itself. Nevertheless, the direct application of PAM to large-scale problems such as single-cell analysis had been prevented by implementation problems, mainly due to memory requirements and long execution times. The most commonly used PAM implementation, the R package *cluster* [21], cannot manage more than 65,536 observations/cells, and the distances are saved using double precision; therefore, any R package based on the cluster package inherits these constraints (as is the case of RaceID2 [22]).

Our implementation of the PAM-BS method (named after PAM with BUILD + SWAP) in the R package *scellpam* [23], allows the choice of the distance data type and yields a memory reduction of 50% using float precision vs. double. Further details of this implementation and its possibilities are included in Sect. 2.2.2 (description of the original algorithms) and Sect. 3.1.1 (computational evaluation of our implementation).

Although we primarily aimed to provide a reliable software implementation of PAM that can manage a large number of single cells, we have an equal interest in analyzing those changes in the human endometrium that occur during the menstrual cycle using PAM as the clustering method.

During a natural human menstrual cycle, the endometrium undergoes shedding, complete regeneration, and remodeling. The relevance of this cycle to human reproduction has driven many studies since its first description, from classical histology [24] to whole-tissue transcriptomic profiling [25, 26]. Current knowledge at the single-cell level [27] has provided insight into the temporal gene expression changes in the epithelial and stromal cell compartments; however, how transcriptomic changes become reflected in the shape of detected cell populations remains incompletely explored.

## Materials and methods

### Menstrual cycle data

The dataset analysed later in the biological application is described bellow. scRNA-seq 10X data was collected from a previous study on the natural menstrual cycle [27] (available in the GEO repository under accession number GSE111976). Fresh endometrial biopsies from healthy individuals were used in this previous study. Isolation and sequencing protocols are detailed in a previous publication.

Raw expression counts per cell formed the input material to Seurat package (v.3.1.2) and downstream scripts in R. Briefly, count matrices were evaluated by quality control metrics that included feature counts ($\ge 2500$ counts per cell), the number of genes detected ($\ge 750$ genes per cell), and the percentage of expression of mitochondrial genes ($\le 25\%$ mitochondrial ratio per cell), and then submitted to dimensional reduction, default clustering, and identification of cell populations. Multiple sets of highly variable genes (HVGs) were obtained using *FindVariableFeatures* with the *vst* method containing: 100, 500, 1000, 2000, 3000, 4000, and 5000 HVGs. Each cluster differentially expressed genes were assessed (one vs. rest) using the *FindMarkers* function (which internally uses the Wilcoxon test), the p-values were adjusted using the Benjamini-Hochberg method, and the false discovery rate (FDR) used was 0.05.

Potential poor-quality clusters with no uniquely expressed genes, doublets (detected by DoubletFinder 2.0.2), or enriched in cells with extreme values in the three quality metrics cited above were evaluated. Finally, no low-quality cluster was found, and 71,032 cells in total were retained for downstream analysis.

### Methods

#### Outline

**Fig. 1:**
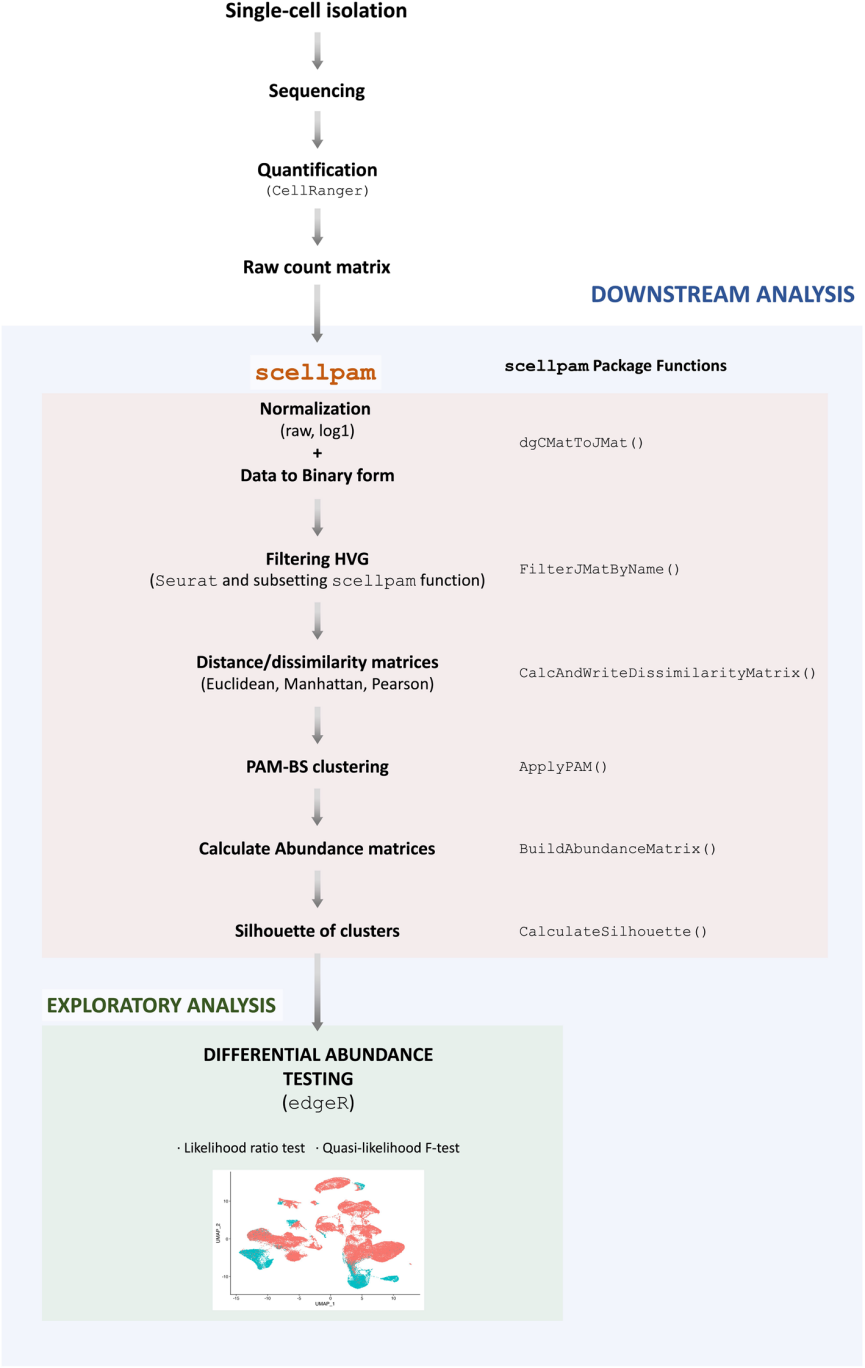
Workflow for applying *scellpam* to the analysis of the changes in cell abundances in the endometrium during the menstrual cycle

Figure 1 provides a detailed outline of our approach for analyzing RNA-seq data.

1. The process starts with the raw count matrix, which provides the short read aligned counts for different cells and samples.
2. The raw count matrix was normalized using two different procedures: *rawn* and *log1n*. The *rawn* method normalizes the expression of each gene in each cell, by dividing it by the total cell count. The *log1n* method is analogous, but with the logarithm of the counts plus one.
3. The highly variable genes (HVGs) in the dataset were identified using the *vst* method implemented in the FindVariableFeatures function of the R package Seurat [15], and this list is used by *scellpam* to filter out HVGs.
4. The dissimilarity matrix of the cells is calculated by using the *scellpam* package [23], where the Euclidean and the Manhattan metrics, alongside one minus the modulus of the *Pearson* correlation coefficient have been implemented. A dissimilarity matrix for each metric is also calculated.
5. The whole set of cells is classified into *k* groups using *PAM-BS* implemented in *scellpam*.
6. The number of cells for each cluster and sample is calculated.
7. Finally, the likelihood and quasi-likelihood approaches proposed in [7, 8] respectively are used to evaluate the differential abundance among the obtained clusters for the different biological conditions or samples.

Additional file 1 contains the entire workflow in R code and applied functions from the new *scellpam* package.

#### Algorithms

We first review the algorithmic principles of PAM to understand its suitability in this context.

Let *X* be a set of *n* points (in this case representing cells) in a p-dimensional space (representing gene expression), and let *d* be a metric or dissimilarity between them. Let *k* be the number of groups being considered. The method obtains an optimal set $M\subset X$ consisting of *k* points called medoids $M=\{x_{m_1},..,x_{m_k}\}$ taken from *X* which minimizes to

$$\begin{aligned} TD = \sum _{i=1}^n d(x_i,x_{m_i}), \end{aligned}$$

where the sum extends to all points in *X* and $x_{m_i}$ is the element of *M* such that $m_i = arg \min _{k\in M} d(x_i,x_{m_k})$ i.e. the medoid closest to each $x_i$. This induces clustering: each cluster comprises the points closer to medoid *k* than any other point. This subsequently implies that cluster representatives (the medoids) always represent members of the initial set, different from other algorithms such as k-means.

The algorithm for obtaining the set *M* entails two stages: selecting an initial set of medoids and swapping pairs of points between the set *M* and the rest of the set *X* until no further reduction of *TD* is found.

We refer the reader to [28] for additional information on the available options for these stages, including the BUILD and LAB alternatives for the first stage.

The algorithm commonly selected for the initial stage is BUILD due to the superior quality of results (a lower initial *TD* value compared to alternatives like LAB in all our datasets); however, BUILD is more computationally intensive.

For the second stage, the algorithm called FATSPAM1 in [20] represents the best option balancing speed and quality of results.

FASTPAM1 is the fastest known algorithm that proceeds deterministically (swapping is always carried out by choosing the option that reduces TD to the most significant degree at each iteration). It is the one implemented in *scellpam*.

The pseudocodes for BUILD and FASTPAM are shown as Algorithms 1 and 2, respectively. Both have been taken from [20].

**Algorithm 1:**
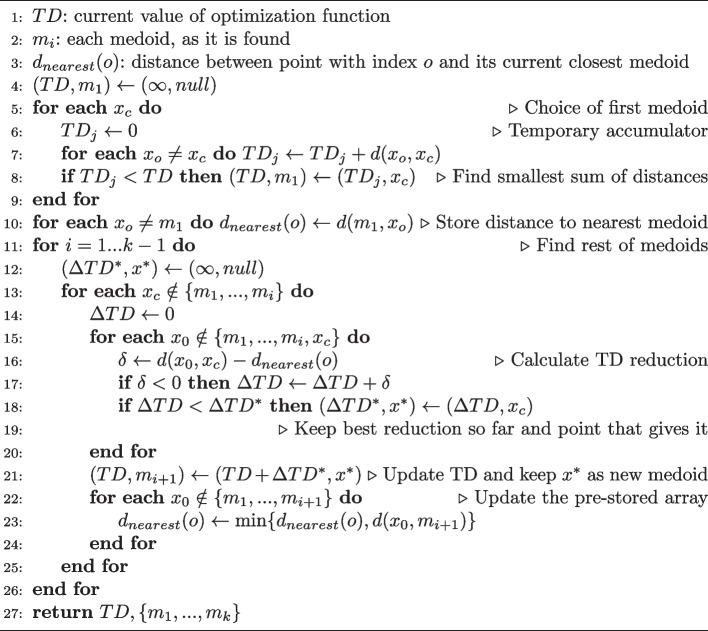
BUILD algorithm. The first initial medoid is found by the loop in lines 5–9. The rest of the medoids require two nested loops (those in lines 10–26 and 13–25). The first loop runs through all points not yet found as medoids. The second loop does the same but takes into account the distance of each point to its closest medoid ($d_{nearest}$) to easily calculate the contribution of such point to the global distance, *TD*. $d_{nearest}$ must be updated in the inner loop (lines 23–24). Our implementation executes the inner loop (lines 13-25) in parallel by groups of points, dividing the points into as many groups as simultaneous threads.

**Algorithm 2:**
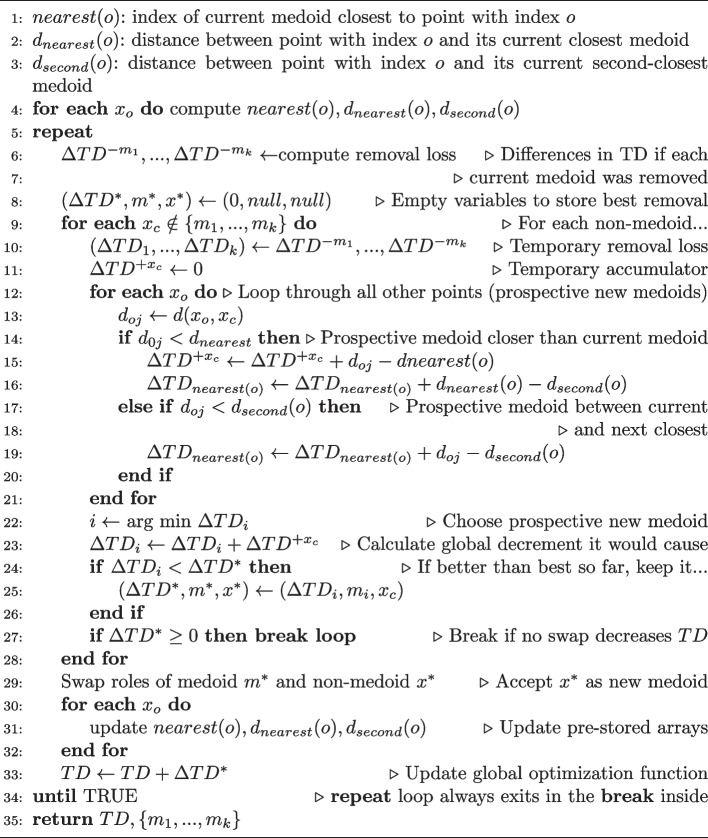
FASTPAM1 algorithm. This algorithm exchanges one point at a time between the current set of medoids and the rest of the points selecting the exchange that most reduces *TD*. Many possible swaps are eliminated by clever use of two arrays of distances, $d_{nearest}$ and $d_{second}$ which hold the distances of each point to its nearest and to its second-nearest medoid; they must be updated (loop in lines 30–32). The global structure contains two nested loops (lines 9–28 and lines 12–21). Our implementation runs the inner one (lines 12–21) in parallel dividing the set of points into as many groups as simultaneous threads.

Our implementation of PAM, programmed in C++, involves several improvements concerning previous packages:

- Parallel computing is transparently used (i.e.: without user intervention) in many parts. The number of cores used can be selected automatically, and work division is carried out automatically and internally by all functions.
- Our implementation allows the use of any storage data type (signed/unsigned int, float, double) for the original data *X* and either float or double for the distance matrix *d* (i.e., float can be used instead of double, which requires half the memory if the precision is sufficient for the user’s purposes).
- The distance matrix calculation, a step prior to PAM, is carried out in parallel. The parallelization has been carefully designed, i.e., the number of distance pairs calculated by each thread is the same, so that all threads finish simultaneously and the time spent by one thread is the total execution time. (See [29] for more details).
- As in other packages (*Seurat, SC3*), initial data *X* can be loaded as a sparse matrix, which significantly reduces the memory requirements. Moreover, this fact is also considered in the distance calculation: components whose value is equal to zero in both vectors are bypassed and components with value equal to zero in exactly one of the vectors receive special treatment, which contributes to the time reduction.
- The choice of the initialization phase for PAM can be BUILD or LAB, and a parallel implementation compensates for the higher computational cost of BUILD. The internal loop in lines 13–25 of the Algorithm 1 is done in parallel.
- Similarly, the SWAP phase uses the fastest variant (FASTPAM1), and is also implemented in parallel (loop in lines 12–21 of the Algorithm 2).
- A function for calculating silhouettes [30] is also implemented in parallel.

For each cell *i*, the silhouette index *s*(*i*) is obtained as follows,

$$\begin{aligned} s(i)=\frac{b(i)-a(i)}{max\left\{ a(i),b(i)\right\} } \text{ if } \mid C_I\mid >1 \text{ and } \text{0 } \text{ otherwise } \end{aligned}$$

where $\mid C_I \mid$ is the number of points in cluster *I* and

$$\begin{aligned} a(i)=\frac{1}{\mid C_I\mid -1}\sum _{j\in C_I,i\ne j}d(i,j) \end{aligned}$$

and

$$\begin{aligned} b(i)=min_{J\ne I}\frac{1}{\mid C_J\mid }\sum _{j\in C_j}d(i,j) \end{aligned}$$

*d*(*i*, *j*) representing the dissimilarity between points *i* and *j*.

The silhouette index *s*(*i*) belongs to $[-1, 1]$. It is close to +1 when the point is well-centered in its own cluster. It is nearly 0 for points in the border between two clusters, and approaches -1 when the point would be better classified in its neighboring cluster. The average of the *s*(*i*)’s over all observations (cells) measures how well the data have been clustered or the cluster structure.

## Results

We present two types of results in this section: one focuses on the performance of the implementation of PAM-BS in *scellpam*, and the other concerns the study of changes in the endometrium. More precisely, Sect. 3.1, which evaluates the performance of PAM-BS, is divided into two subsections: Sect. 3.1.1 is devoted to evaluating purely computational aspects of the implementation of PAM-BS in *scellpam*. In this case, there is no “true” partition to compare against.

After the computational study (in terms of time and memory usage depending on the number of cells and genes), Sect. 3.1.2 is dedicated to the comparison between the classifications obtained by other clustering methods and those obtained by PAM-BS when “true” partitions are known, which is based on study by Duó et al. [9]. Here, we retrieve the results obtained by the authors in their comparison of different clustering methods on several datasets for which the true partition is assumed to be known. Then, we implement our method on these same datasets. Section 3.2 is the most interesting one, from a biological perspective, as it presents the results of our study of cell population changes that occur in the human endometrium during the secretory phase of the menstrual cycle.

### Two studies on PAM-BS performance

#### Computational comparisons

We compare the results obtained by applying the implementation of PAM-BS in *scellpam* with those obtained by the standard implementation of PAM in the *cluster* R package [21].

The first factor to validate is the use of the float data type. Obviously, using float reduces the memory by half, but it must be corroborated that results are equivalent. To validate our implementation, we took several samples of 60,000 cells from a total of 71,032 cells (due to the limitation of the package *cluster* to 65,536 observations). The use of double type requires a memory of 13.76 GiB to store the distance matrix; our package using float type requires 6.70 GiB. Clustering results were the same in all cases (same set of medoids *M*), which supports the correctness of our implementation (and also that of the *cluster* package) in algorithmic terms, indicating the probable absence of programming errors, since both implementations were created by different teams and do not share any code. Regarding execution times, we observed an almost linear time reduction in the distance matrix calculation with the number of distances computed (not the case for the *cluster* package). The structure of the PAM algorithm indicates that a time reduction strictly linear with the number of cells cannot be expected, but it was substantial. We evaluated the time performance and memory usage of PAM-BS using different datasets with increasing cell number sizes:

1. scRNA-seq on the human endometrium throughout the natural menstrual cycle; $N=71,032$ cells. NCBI GEO accession number GSE111976 [27], which we denoted as **Wang**.
2. scRNA-seq of superficial endometrial biopsies; $N=100,307$ cells. ArrayExpress accession number E-MTAB-10287 [31], which we denoted as **Garcia**.
3. scRNA-seq of the endometriotic endothelium; $N=118,144$ cells. NCBI GEO accession number GSE213216 [32], which we denoted as **Fonseca**.
4. The merging of the previous three datasets; $N=289,483$ cells, which we denoted as **Merge**.

We also compared PAM-BS to other broadly used clustering methods: Scanpy (1.9.3) [17], SC3 (1.28.3) [11], and Seurat (3.1.2) [13]. RaceID2 [22] could not be applied to any dataset due to memory exhaustion, even with the smallest dataset (Wang).

We made comparisons using the same computer (AMD-Ryzen Threadripper 3990X processor at 2.2GHz) with 128 GiB RAM exclusively devoted to this task. As the distance matrix calculation is only required as a first step by PAM, our package cannot be compared in this respect with Scanpy, Seurat or SC3. To make a meaningful comparison, we used the daisy function of the *cluster* package on a sample of 35, 516 cells. Calculating the distance matrix with daisy took approximately 6.25 days. The *scellpam* package took 18 h using the serial version (one thread) and 27 min with parallel implementation (with 128 threads) using double as data type.

Table 1 contains the results of the distance matrix calculation for *Pearson* dissimilarity with *scellpam* using 128 threads in the above datasets in terms of time spent (in s) and memory used (in MiB).

**Table 1:** Time in s and memory used in MiB for Pearson dissimilarity matrix calculation with 128 threads

		Number of genes				
Data	Cells	100	500	4000	All	Memory
Wang	71,032	5.69	29.43	228.25	1542.55	9623
Garcia	100,307	11.12	52.09	457.08	3133.30	19191
Fonseca	118,144	14.75	69.82	581.39	5397.11	26623
Merge	289,483	93.57	450.63	3755.06	40367.32	159837

Concerning the complete execution, application of PAM-BS to the aforementioned subset of 35, 516 cells takes approximately the same amount of time to package *cluster* (290 s) as our package *scellpam* (272 s) when using a single thread; however our parallel implementation reduced this time to 14 s.

The entire Wang set (71, 032 cells) cannot be managed by *cluster*; however, *scellpam* used 33 min to calculate the distance matrix and 3 min and 17 s for PAM-BS to run (both in parallel). Additional file 2 contains a table describing all results. This results demonstrate that, even if further reductions can still be achieved, integration with R and the overall ease of use favor the feasibility of our current approach.

Table 2 displays the results obtained by applying PAM-BS, using 64 threads for the aforementioned datasets with 30 medoids. and presents spent time (in s), used memory (in MiB), and the number of medoid swap iterations.

Of note, the number of SWAP iterations in the second phase of PAM-BS mainly determines the total time; the number of genes has no relevance here as this number has been subsumed when calculating the distance/dissimilarity matrix.

**Table 2:** Time (in s), used memory (in MiB) and number of SWAP iterations for PAM (BUILD+SWAP) calculation with $k=30$ using 64 threads

		Number of genes and number of iterations (It.) in SWAP								
Data	Cells	100	It.	500	It.	4000	It.	All	It.	Memory
Wang	71,032	488.65	40	399.41	17	349.43	18	293.96	15	10119
Garcia	100,307	1021.61	36	662.62	12	677.60	13	598.16	12	20155
Fonseca	118,144	1236.65	26	1049.29	17	1048.90	17	770.60	8	21660
Merge	289,483	14677.80	41	9908.06	16	11069.50	22	7810.15	10	169502

Other trials carried out with a number of medoids between 25 and 45 provided comparable results: total time for PAM in the Wang dataset varied from 159 to 252 s, increasing with the number of medoids. Additional file 2 contains a table with the details of this experiment.

Of note, while the algorithm that computes the silhouettes does not involve a significant time cost, it gains an advantage from parallelization; for example, the execution time associated with the Wang dataset becomes reduced from approximately 18 s in serial to 7 s using 64 threads.

Finally, we applied the Scanpy, SC3 and Seurat methods to the same datasets. Table 3 reports the results regarding execution time (in s) and used memory (in Mib).

**Table 3:** Time (in s) and memory (in MiB) for execution of Scanpy, SC3 and Seurat methods in several single cell banks

			Method					
Data	Number of	Number of	Scanpy		SC3		Seurat	
set	cells	genes	Time	Memory	Time	Memory	Time	Memory
Wang	71,032	100	81.97	9194.92	(1)		123.75	483.89
		500	100.69	9541.20	(1)		151.63	1238.48
		4000	229.94	11786.49	2907.28	8334.96	299.47	5828.26
		all	178.63	11572.80	6202.98	40560.79	717.55	27332.94
Garcia	100,307	100	117.89	13616.54	(1)		202.43	476.88
		500	142.09	14174.46	(1)		204.96	940.13
		4000	338.65	17515.35	3047.56	8411.55	347.43	5397.71
		all	340.17	12055.15	6050.07	51085.32	1198.42	32640.66
Fonseca	118,144	100	120.84	8014.76	(1)		191.96	448.31
		500	159.91	8944.68	(1)		252.76	1002.43
		4000	388.47	12011.64	2606.85	9017.33	415.30	4996.09
		all	178.63	11572.80	4752.91	73529.04	1678.49	31231.88
Merge	289,483	100	638.00	30738.78	(1)		631.02	950.68
		500	490.14	32033.64	(1)		706.44	2266.57
		4000	1111.51	40586.07	2960.67	21616.60	1017.365	13205.54
		all	1454.21	41961.79	(2)		(2)	

SC3 was applied with 64 threads; Scanpy and Seurat do not allow the number of threads to be chosen

(1): Application of SC3 with 100 and 500 genes caused a program error

(2): Program crashes without error messages due to memory exhaustion on a 256 GiB machine

SC3 was unsuccessful with 100 and 500 HVGs as the dimensionality reduction phase produced singular matrices; moreover, both Seurat and SC3 failed when applied to the most extensive cell set containing all genes due to insufficient memory.

Nevertheless, it is fair to remark that these methods were designed for application with smaller sets of relevant genes, rather than in a highly dimensional space; however, it is also true that PAM-BS is not subject to this limitation as dimensions are subsumed after distance calculation.

This factor provides an advantage, but it also makes PAM slower than Scanpy. Additionally, PAM-BS is also slower than Seurat when not all genes are used. This increase in comparative speed derives from the fact that these packages use hierarchical clustering algorithms (the Louvain community detection algorithm, [18]). In contrast, PAM represents a clustering algorithm that considers all distances between cells in all steps, which allows the cluster reassignment of any cell at any step.

#### Comparison of classifications

As noted above, this section uses a part of the study conducted by Duó et al. [9] The partitions obtained by the authors can be retrieved from their *DuoClustering2018* package [33]. This allowed the comparison of our own results with those of the other methods. While we provide information regarding the methodologies, the datasets analyzed and their conclusions for clarity we refer the reader to [9] for a more detailed explanation.

The authors considered and evaluated 14 clustering algorithms using 12 datasets: 9 real datasets and 3 simulated datasets. The true partition of each dataset is known. Three methods to reduce the number of genes provided as input to the clustering methods were used for each dataset. This provides $12 \times 3 = 36$ possibilities to evaluate the algorithms.

Table 1 in [9] provides an overview of the datasets used in the study (e.g. sequencing protocol, number of cells, number of features...), while Table 2 describes each method. We include the information shown in Table 2 to improve the readability of this paper. The clustering methods compared are:

- *ascend (v0.5.0)*: Principal Component Analysis (PCA) dimension reduction (dim=30) and iterative hierarchical clustering.
- *CIDR (v0.1.5)*: PCA dimension reduction based on zero-imputed similarities followed by hierarchical clustering.
- *FlowSOM (v1.12.0)*: PCA dimension reduction (dim=30) followed by self-organizing maps $(5 \times 5, 8 \times 8$ or $15 \times 15$ grid, depending on the number of cells in the dataset) and hierarchical consensus meta-clustering to merge clusters.
- *monocle* (v2.8.0): t-SNE (t-distributed stochastic neighbor embedding) dimension reduction (initial PCA dim=50, t-SNE dim=3) followed by density-based clustering.
- *PCAH*: PCA dimension reduction (dim=30) and hierarchical clustering with Ward.D2 linkage.
- *PCAKmeans*: PCA dimension reduction (dim=30) and k-means clustering with 25 random starts
- *pcaReduce (v1.0)*: PCA dimension reduction (dim=30) and k-means clustering through an iterative process; stepwise merging of clusters by joint probabilities and reducing the number of dimensions by PCA with the lowest variance; repeated 100 times following consensus clustering using the clue package ([34, 35]).
- *RACEID2 (version: March 3, 2017)*: k-medoids clustering based on *Pearson* correlation dissimilarities.
- *RtsneKmeans*: t-SNE dimension reduction ((initial PCA dim=50, t-SNE dim=3, perplexity = 30) and k-means clustering with 25 random starts.
- *SAFE (v2.1.0)*: Ensemble clustering using SC3, CDIR, Seurat and t-SNE + k-means.
- *SC3 (v1.8.0)*: PCA dimension reduction or Laplacian graph. k-means clustering on different dimensions; hierarchical clustering on consensus matrix obtained by k-means.
- *SC3svm (v1.8.0)*: Using SC3 to derive the clusters for half of the cells, then using a support vector machine (SVM) to classify the remaining cells.
- *Seurat (v2.3.1)*: Dimension reduction by PCA (dim=30) followed by nearest neighbor graph clustering.
- *TSCAN (v1.18.0)*: PCA dimension reduction followed by model-based clustering.

The hyperparameter values for all clustering algorithms and datasets can be accessed using the function duo_clustering_all_parameter_settings_v2() in the package [33]. For instance, the Seurat range resolutions for the KumarTCC dataset filtered by HVG10 ranged from 0.3 to 1.5 in increments of 0.1. Additional file 3 provides additional details.

The authors of [9] employed nine publicly available scRNA-seq datasets and three simulated datasets with varying degrees of separation to evaluate the methods. Table 4 (a reduced version of Table 1 in [9]) notes the dataset names and the number of cells, features, and true populations to be recovered.

**Table 4:** Overview of the datasets in [9].

Dataset	# cells	# feat.	# pop.
Koh	531	48,981	9
KohTCC	531	811,938	9
Kumar	246	45,159	3
KumarTCC	263	803,405	3
SimKumar4easy	500	43,606	4
SimKumar4hard	499	43,638	4
SimKumar8hard	499	43,601	8
Trapnell	222	41,111	3
TrapnelTCC	227	684,953	3
Zhengmix4eq	3,984	15,568	4
Zhengmix4uneq	6,498	16,443	4
Zhengmix8eq	3,994	15,716	8

The names of the simulated datasets are prefixed with “Sim”

The names of the three gene filtering methods are *Expr10* (10% genes with the highest average expression), *HVG10* (10% most highly variable genes, HVGs) and *M3Drop10* (the drop-out rate of the genes represents a function of the mean expression level, which keeps 10% of genes.

Duó et al. [9] aimed to assess the ability to recover known populations, the run times, and the stability of the methods.

The authors used the Adjusted Rand Index (ARI) [36] to compare two partitions to evaluate how well the clusters recovered the true populations. This index measures the agreement between two classifications, not necessarily with the same number of clusters. If $P=\{ 1,\ldots s\}$ and $P^*=\{ 1,\ldots ,r\}$ denote two partitions of a given dataset with *s* and *r* clusters respectively, the ARI($P,P^*$) is defined as

1 $$\begin{aligned}{} & {} \frac{Index-Expected\_index}{Max\_index-Expected\_index}= \nonumber \\{} & {} \frac{\sum _i\sum _j\left( {\begin{array}{c}n_{ij}\\ 2\end{array}}\right) -\left[ \sum _i\left( {\begin{array}{c}a_i\\ 2\end{array}}\right) \sum _j\left( {\begin{array}{c}b_j\\ 2\end{array}}\right) \right] /\left( {\begin{array}{c}n\\ 2\end{array}}\right) }{\frac{1}{2}\left[ \sum _i\left( {\begin{array}{c}a_i\\ 2\end{array}}\right) +\sum _j\left( {\begin{array}{c}b_j\\ 2\end{array}}\right) \right] - \left[ \sum _i\left( {\begin{array}{c}a_i\\ 2\end{array}}\right) \sum _j\left( {\begin{array}{c}b_j\\ 2\end{array}}\right) \right] /\left( {\begin{array}{c}n\\ 2\end{array}}\right) } \end{aligned}$$

where $n_{ij}$ is the number of individuals belonging to class *i* in the first clustering (*P*) and to class *j* in the second one ($P^*$), $a_i$ ($1 \le i \le r$) is the number of individuals in class i in $P^*$ and $b_j$ ($1 \le j \le s$) is the number of individuals in class j in *P*.

While this index has been widely used to quantify agreements between two partitions for all clusters simultaneously, it is not straightforward to interpret. The closer the index value is to 1, the better the agreement; however, a tendency to mainly reflect the degree of agreement between the partitions on the large clusters must be considered. Clusters with few elements have less influence on ARI values [37].

Within the conclusions (page 7 in [9]), the authors state that the highest ARI values are obtained for the “well-separated datasets” (Kumar, KumarTCC and SimKumar4easy). “All the methods failed to recover the partition of the cells by time point in the Trapnell datasets, where the ARIs were consistently below 0.5.” They also noted that “the M3Drop filtering consistently led to a worse performance for the simulated datasets, while the performance was more similar to the other filterings for real datasets”. They also comment, “While none of the methods consistently outperformed the others $\ldots$ *SC3* and *Seurat* often showed the best performance.”

The partitions obtained by applying each method on each dataset for different values of the number of clusters (*k*) can be retrieved using functions included in [33]. For instance, the function clustering_summary_filteredExpr10_KohTCC_v2() contains the clustering results from the performance evaluation of the clustering methods analyzed in [9] when tested on the dataset KohTCC filtered by Expr10.

Our primary interest was evaluating whether our implementation of PAM produces ARI values similar to those obtained by other methods.

Additional file 3 includes the R code for the following analysis and a detailed discussion.

- For each method and dataset in [33], the true partition is known. The partition obtained when *k* is equal to the true number of clusters is obtained; then the ARI between both is calculated.
- The *scellpam* package allows the choice of the dissimilarity measure ($L_1$, $L_2$ and *Pearson*), the normalization method (*rawn* and *log1n*), and the number of clusters *k*; for each dataset, *k* is set as its true number of clusters and PAM-BS was applied for all six possible combinations of dissimilarity and method, and for the 36 datasets mentioned above.
- Comparisons were carried out between the partitions obtained by each clustering method and the true partitions, resulting in an ARI value for each case. Table 5 displays the ARI scores for the different datasets in [9] and the three filtering methods obtained by applying PAM-BS with the options $L_1$ and *rawn*. Additional file 3 reports the ARI scores for all the methods and datasets in Tables 1, 2, 5, 6, 9 and 10. Tables 1 and 2 refer to the datasets filtered with the Expr10 procedure, Tables 5 and 6 refer to those filtered with HVG10, and Tables 9 and 10 refer to those filtered with M3Drop10.
- We performed three separate analyses, one for each gene filtering method as each produces a different dataset when applied to the same initial dataset. We used the Friedman rank sum test [38] for each analysis to evaluate for differences between methods. We used the implementation by Eisinga et al. [39] to perform exact all-pairs comparison tests for the post-hoc analysis. We excluded the *ascend* and *SAFE* methods from the comparison as they failed to return a partition with the true number of groups for some datasets.

**Table 5:** Each dataset in the first column has been filtered with the three gene filtering methods, giving rise to three different datasets.

Dataset	Expr10	HVG10	M3Drop
KohTCC	0.47	0.44	0.47
Koh	0.51	0.47	0.74
KumarTCC	1.00	1.00	0.98
Kumar	0.97	0.97	0.95
SimKumar4easy	1.00	1.00	0.18
SimKumar4hard	0.58	0.60	0.00
SimKumar8hard	0.22	0.20	0.01
TrapnelTCC	0.36	0.36	0.40
Trapnell	0.32	0.36	0.22
Zhengmix4eq	0.83	0.89	0.76
Zhengmix4uneq	0.77	0.75	0.77
Zhengmix8eq	0.54	0.56	0.48

Clusters were obtained using PAM-BS,distance, and *rawn* normalization. Columns 2, 3, and 4 contain the ARI scores obtained when the partitions obtained by PAM-BS are compared with the true partitions. The closer to 1, the better the agreement between the partitions

We now report the results obtained using the Expr10 gene filtering method. Additional file 3 details the results obtained with HVG10 and M3Drop.

The conclusion was to reject the null hypothesis of no difference between methods (Friedman chi-squared = 84.243, df = 17, p-value = 6.699e$-$11).

Concerning the post-hoc analysis, we found no significant differences ($\alpha =0.05$) between PAM-BS with normalization=*rawn* and distance=*L1* and SC3, Seurat, or any other method. However, when we parameterize PAM-BS with other combinations of normalization and distance, we found significant differences with some methods, such as SC3 or Seurat, which performed better. Tables 3 and 4 in Additional file 3 contain the p-values corresponding to the all pairs of comparisons.

Additional file 3 details the results obtained with HVG10 and M3Drop (Tables 7 and 8, and 11 and 12, respectively).

**Fig. 2:**
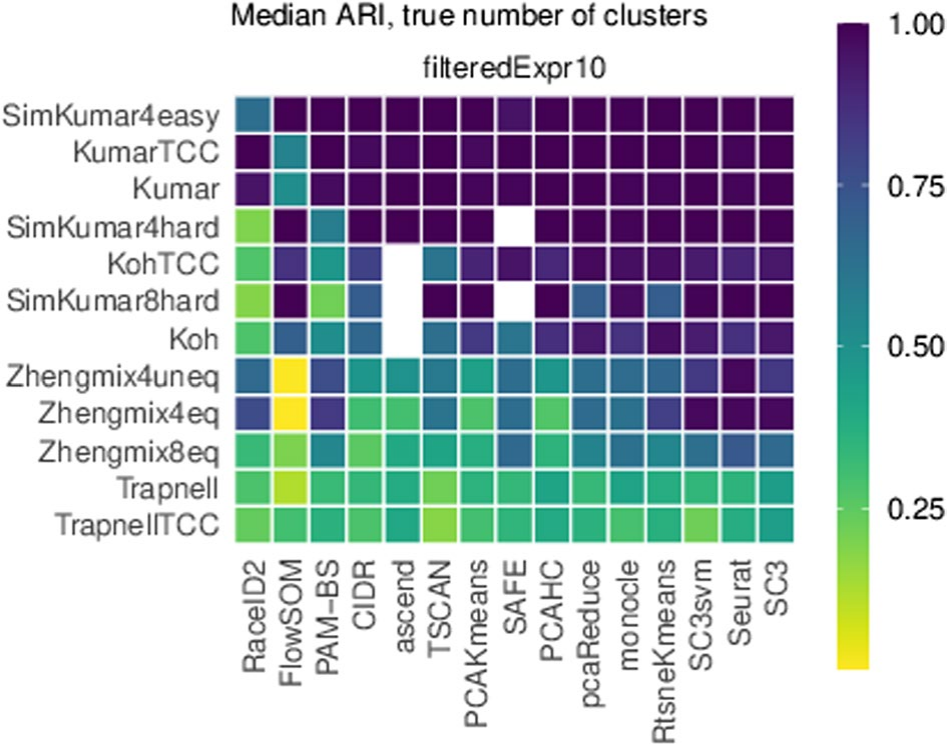
Heatmap of median ARI scores calculated by each method for each dataset. The order of appearance of the methods and the datasets was determined by plot_performance() and depends on ARI values

We obtained Fig. 2 using the function plot_performance() in [33].Figure 4 in Additional file 3) graphically compares PAM-BS with the other clustering methods. Each row corresponds to a dataset and each column to a clustering method. White squares indicate a method that failed to return clustering with the same number of groups as the true partition for a given dataset.

We executed PAM-BS setting ctype=L1 and normalization = rawn. Given the deterministic nature of PAM-BS, the median ARI is simply the ARI. Concerning ARI values for the remaining methods, they were obtained from the information retrieved from [33], as explained in the text.

As a qualitative summary of this section, PAM-BS does not display a significant difference ($\alpha =0.05$) with any other clustering methods for the studied datasets filtered by HVG10 or Expr10 when making an appropriate choice of its parameters (normalization type and distance/dissimilarity choice). However, SC3 performs significantly better than PAM-BS ($\alpha =0.05$) when genes are filtered using the M3Drop method. In general, no method outperforms the remaining methods for all datasets and gene filtering options.

### Human endometrium study

#### PAM-BS clustering and a likelihood-based differential abundance test for the detection of changes in human endometrial cell populations

The primary aim of this paper was to evaluate differential cell abundance throughout the natural menstrual cycle using the number of cells per sample and cluster as inputs. This study searches for possible associations between cluster counts and the phenotypic variables describing the biological samples. The counts and the clustering compactness depend on the selection of the normalization method, the dissimilarity measure, the number of HVGs, and the number of clusters. We evaluated such compactness using the mean silhouette of the clusters [30].

As previously stated, the R-package *scellpam* also provides a parallel calculation of the silhouette index for the resulting clustering. Silhouette width has previously been used in scRNA-seq literature to evaluate clustering performance [40].

**Fig. 3:**
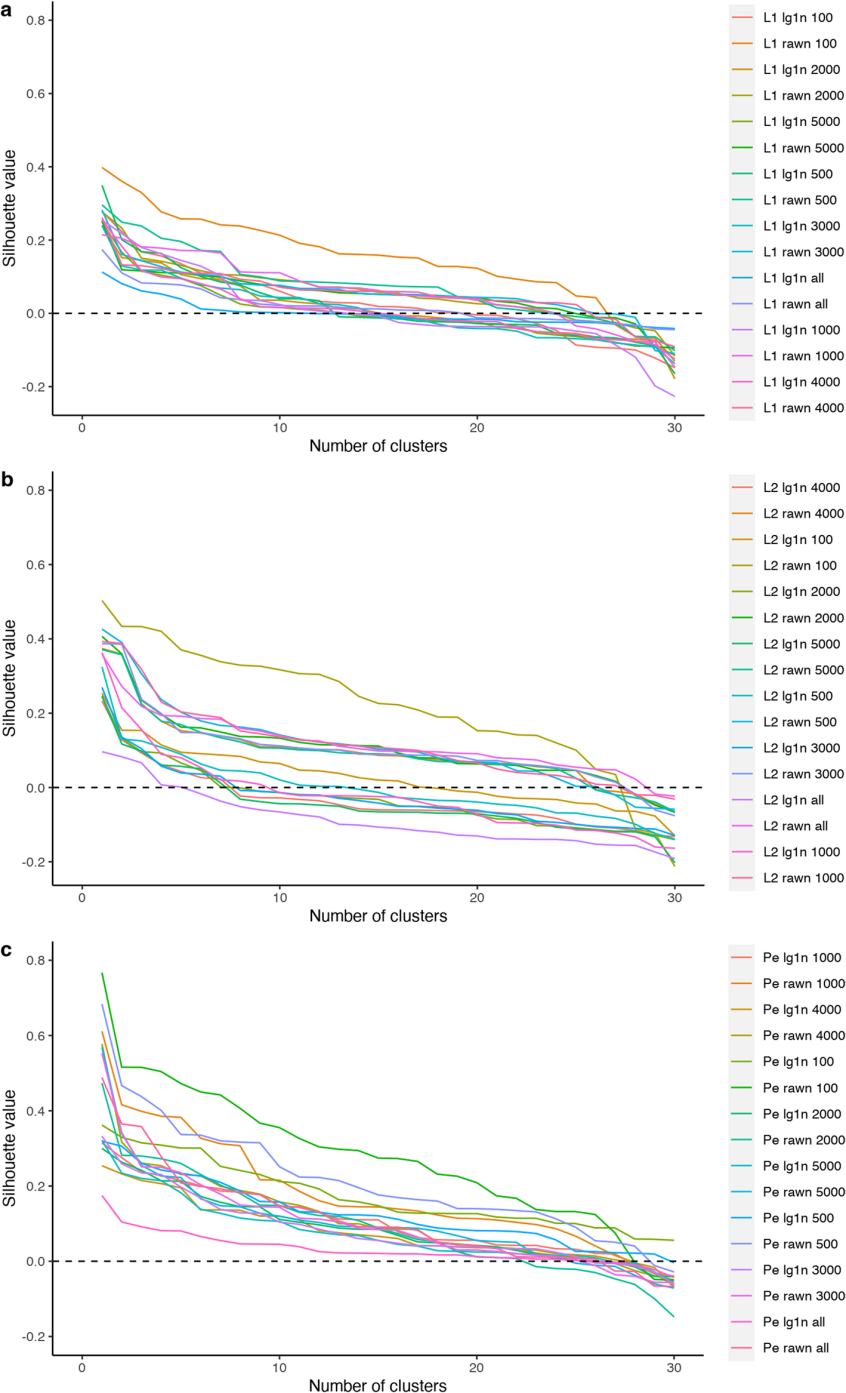
Mean silhouettes for different normalization methods and dissimilarities by taking into account the number of clusters. *L*1: Manhattan distance (a), *L*2: Euclidean distance (b), *Pe*: *Pearson* dissimilarity (c). rawn: refers to normalization. lg1n refers to log normalization. Numbers refer to the number of HVGs included in each combination

In all cases, the distance/dissimilarity used to calculate the silhouette is the same as that employed to build the cluster with PAM. Figure 3a–c demonstrate these mean silhouettes as a function of the number of clusters, dissimilarities, and normalization methods. The primary purpose of silhouette use was to support the choice of the normalization method, dissimilarity, and number of HVGs.

Although the silhouette provides a good proxy of cluster structure, outcomes must be validated by the biological coherence of cell groups, which must exhibit homogeneous expression patterns. We used the silhouette as a guide to set the clustering parameters. Among the combinations with high silhouette, we chose the combination that yielded the strongest signal of abundance changes for well-delimited cell types; thus, we chose normalization with the *log1n* method, *Pearson* dissimilarity and 100 highly significant genes.

We considered three different generalized linear models [7], each using a different predictor. The first model uses **time**, defined as the day of the menstrual cycle; the second model uses **time2**, a binary variable indicating if the day of the menstrual cycle is less than or equal to day 20 (value 0) or greater than 20 (value 1); while the third model uses the predictor **phase**, given in the sample description as the traditional classification of the menstrual cycle phases (level 3 = late proliferative canonical phase; level 4 = early- to mid-secretory canonical phase; and level 5 = late secretory canonical phase). In summary, we can evaluate menstrual time in three manners: one uses the day, while the others employ relevant biological knowledge.

We tested the null hypothesis of no effect of the chosen predictor (i.e., the corresponding coefficient equal to zero) for the two first models, which evaluates the cluster counts’ dependence on the corresponding predictor. The third model has a categorical predictor - the menstrual phase - with three levels; the phase is coded using two dummy variables, and we tested if both coefficients can be jointly considered null. Additionally, we considered comparisons of each pair of levels.

We applied two of the testing procedures implemented in the *edgeR* package. The first [7] assumes a generalized linear model with a negative binomial response. The second quasi-likelihood approach, modifying the mean-variance relationship of the negative binomial model, is proposed in [8]. In our case, the number of significant clusters detected with the quasi-likelihood approach remains lower than with the likelihood approach. Additional file 4 contains the results for both approaches; from this point forward, we will use the likelihood approach.

**Fig. 4:**
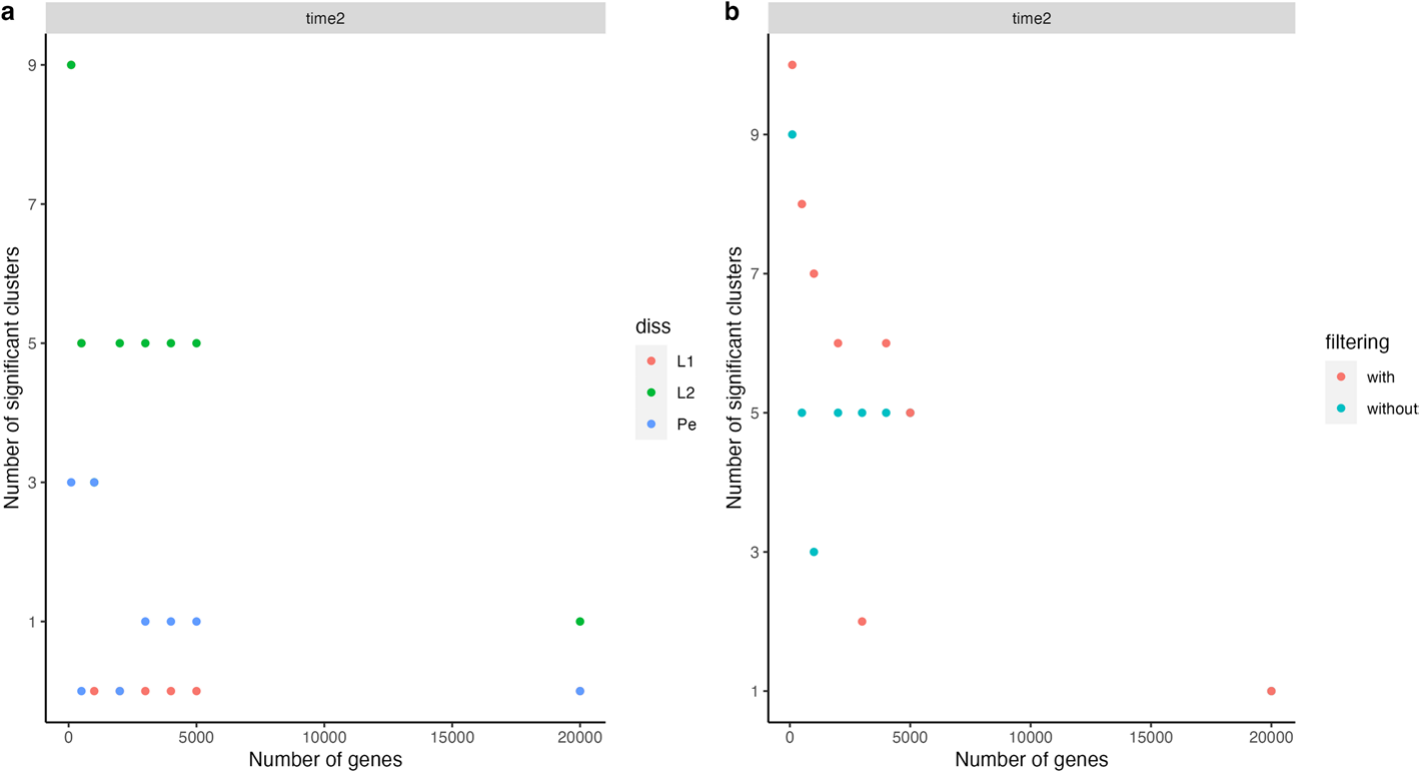
(a). Number of significant clusters plotted against the number of genes using raw normalization and considering the models with the binary time as a predictor. (b). An analogous plot after filtering by silhouette

We observed more significant clusters using raw count normalization instead of logarithmic normalization. Figure 4a displays the number of significant clusters obtained using different dissimilarity measures with **time2** as the predictor. Overall, Euclidean distance appears to provide more significant clusters, followed by the *Pearson* dissimilarity, and finally, the Manhattan metric ($L_1$). The different criteria to choose the normalization and metric seem reasonable, which makes this an open question. Different choices should be evaluated in each separate case.

Figure 4b reports the number of significant clusters using the whole set of cells (labeled **without**) and using a filtered set of cells, considering the silhouette values (i.e., cells with low silhouette values are removed) (labeled **with**). Specifically, we applied successive steps of removing the 15% of cells with a lower silhouette until at least 60% of the remaining cells have a silhouette higher than 0.7 were applied. Cell filtering provides more significant clusters when fitting the models with time and time2; however, this statement does not remain true using the model with phase (as shown in Additional file 4).

Although the model using time2 as a predictor variable encounters fewer significant clusters than the model using phase as a predictor, the first method is preferred given the consistency of results. The number of significant clusters remains similar with and without filtering using the model with time2, while the model with phase as a predictor finds almost all clusters significant without filtering (28 to 30 using different normalization methods and metric/dissimilarity) but a number from 3 to 8 with filtering.

We compared the distinct phases of the menstrual cycle with the corresponding comparisons. Only comparing the two last phases (3 and 4) provided significant clusters using the likelihood approach. Comparisons of time2 levels and phase levels 3 and 4 remain biologically similar since they overlap the natural time frames of the menstrual cycle. Intriguingly, while a few clusters display significance when comparing 3 and 4 phases, the global evaluation of the predictor indicates the significance of almost all clusters. The unbalanced number of samples between phases could yield this result. Additional file 4 contains the global evaluation and the comparisons.

**Fig. 5:**
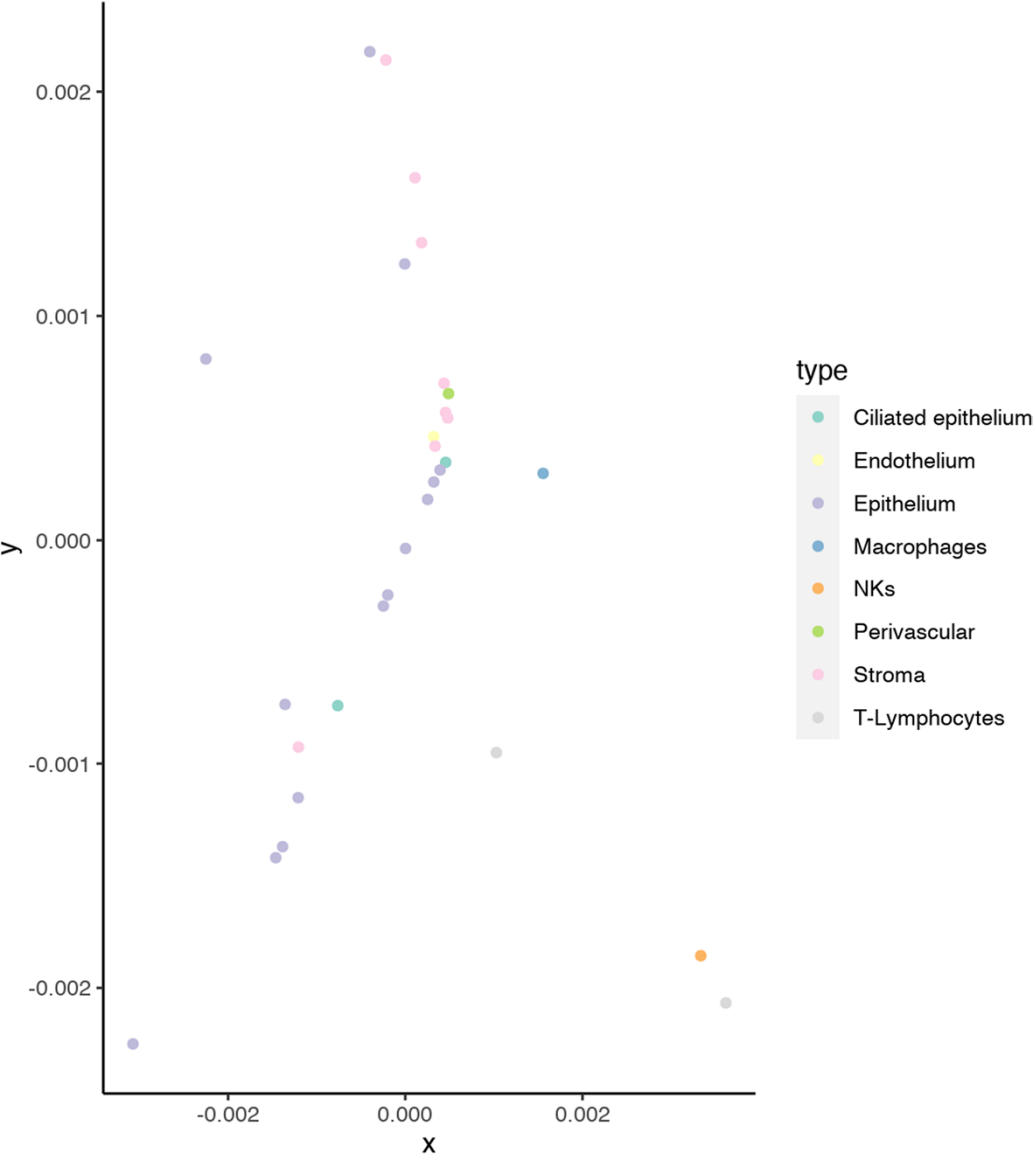
Multidimensional scaling corresponding to obtained medoids using log1 normalization and the *Pearson* dissimilarity

Due to its superior stability across numerous factors, such as choice of normalization, metric/dissimilarity, and silhouette filtering, we used the model with “time2” as the predictor variable in subsequent analyses. We made this choice in favor of consistency and reliability. Figure 5 displays a multidimensional scaling plot corresponding to the medoids obtained using log normalization with a *Pearson* dissimilarity. Medoids from the two most abundant cell types (epithelial and stromal) display trends in opposite directions and increasing distances from the center of the scaling plot, which we interpret as cell states in progression through the menstrual cycle. We observed natural killer (NK) cells and T-lymphocyte medoids positioned far from the center, denoting their distinct immune nature compared to the remaining cell types.

**Fig. 6:**
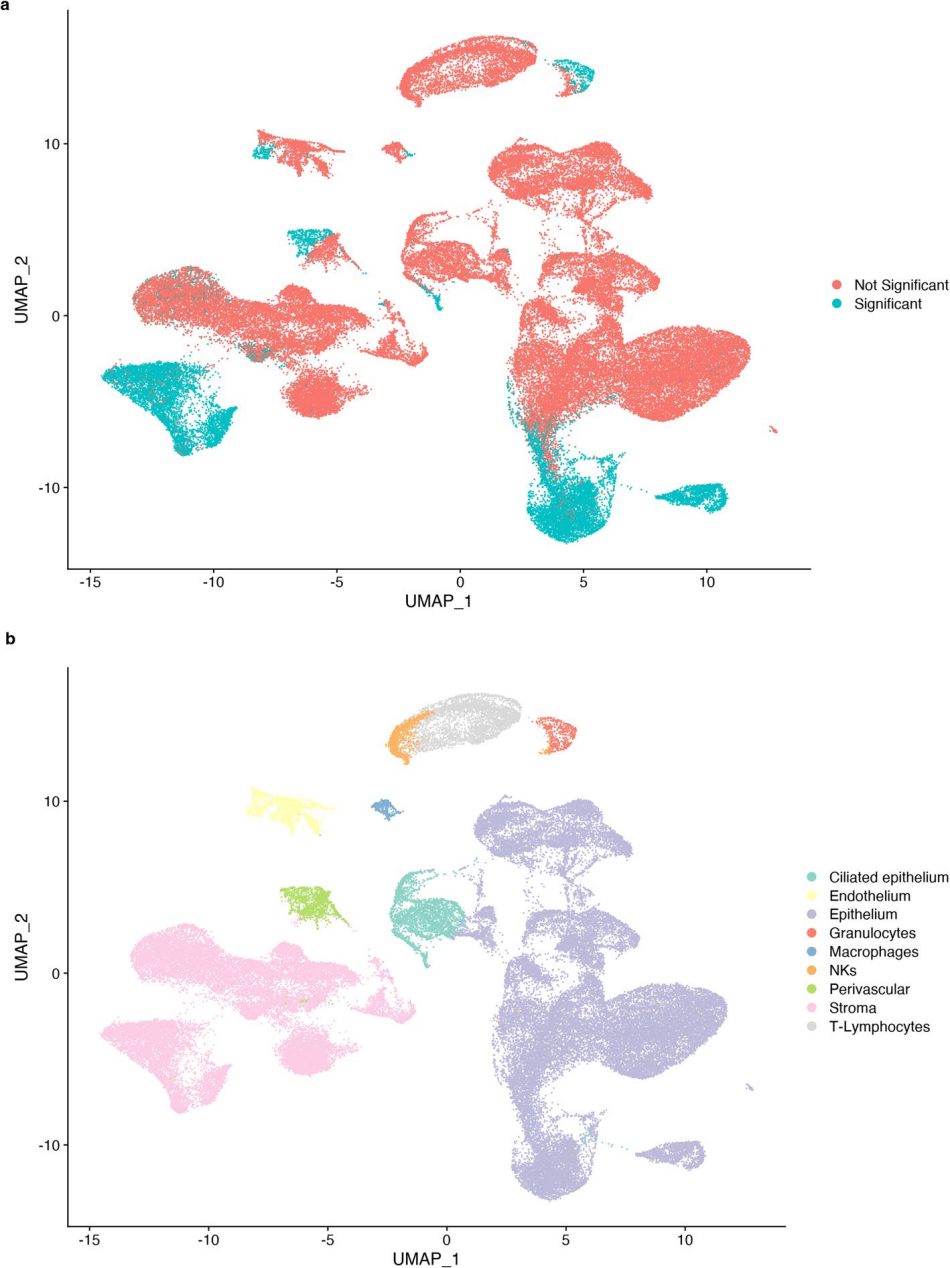
UMAP (uniform manifold approximation and projection) representing endometrial single cells colored by significance after generalized linear model testing of **a** abundance and **b** annotated cell type

Most endometrial cell types annotated in our dataset possessed clusters that reported a significant p-value after day 20 of the menstrual cycle (i.e., epithelium, stroma, endothelium, perivascular cells, and immune cells (Fig. 6b). These cells (colored blue in Fig. 6a) represent the changing subpopulations of each primary cell type that modify their abundance during this time phase.

#### Differentially expressed genes of changing populations have direct involvements in endometrial receptivity

**Fig. 7:**
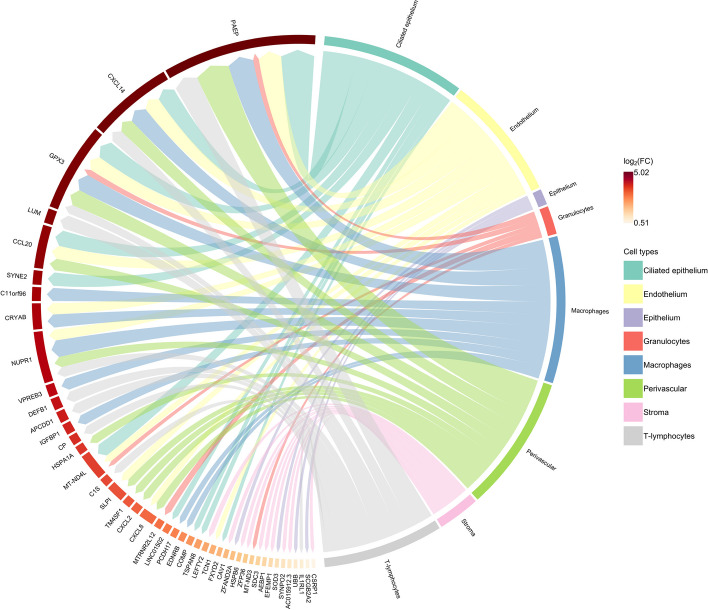
Circos plot of differentially expressed genes by each annotated cell type, comparing each significant cluster at time2 and their peer cells within the same cell type

We performed a differential expression evaluation between each significant population from the abundance test against its peer non-significant cells within each cell type considered. Fig. 7 summarizes the obtained results.

The PAEP, GPX3, and CXCL14 genes, which displayed overexpression in most cell types analyzed, represent reportedly robust markers of the receptive endometrium [41, 42]. PAEP (progestagen-associated endometrial protein or Glycodelin) is a progesterone-regulated gene that regulates critical fertilization steps [43, 44]. The gene encodes four glycoforms differing concerning glycosylation patterns - glycodelin-S, -A, -F, and -C. These glycoforms have distinct, essential roles in maintaining a uterine environment suitable for pregnancy and in the timing and occurrence of the appropriate sequence of events in the fertilization process [45], including immunomodulatory activities. The presence of glycodelins primarily associates with the epithelial compartment [27, 46, 47]; however, our analysis detected the presence of PAEP expression at the single-cell resolution beyond the limits of the epithelium, with significantly altered levels observed in populations within the vascular endothelium, perivascular compartment, and immune cell types (macrophages, T-lymphocytes and granulocytes).

Differential expression analysis in the endothelium also identified genes related to cellular stress (NUPR1) [48], heat shock-related proteins (CRYAB) [49], mitochondria genes (MT-ND4L), and genes related to immune activation and pro-inflammatory response (SLPI and CXCL8). This gene set relates closely to the inflammatory processes in the late secretory phase. This inflammatory phenomenon plays a dual role: first, the endothelial recruitment of immune cells to infiltrate the endometrium and activate the production of embryo adhesion molecules by the luminal epithelial cells [50], and second, the promotion of a controlled pro-inflammatory environment that later supports the regeneration and healing of the endometrium during menstruation [51]. Furthermore, we discovered that a subpopulation of perivascular cells -resident cells of the surrounding blood vessels—expressed CXCL2 and CXCL8, contributing to the pro-inflammatory microenvironment [52]. In short, the identified transcriptomic signature supports using PAM-BS as an effective tool to detect changes in cell abundances between different biological states.

## Discussion

As part of this study, we aimed to investigate how cellular populations change in abundance in a dynamically modulated tissue, the human endometrium, throughout the menstrual cycle. The cellular dynamics of a highly renewable tissue remain challenging to dissect. Physiologically, the endometrium enters a narrow window of receptivity -known as the window of implantation (WOI) [53]- that is structurally and biochemically ideal for embryo implantation [54] during the second half of the menstrual cycle (functionally known as the secretory phase). The duration and precise timing of the WOI are subject to broad inter- and intra-individual variation [24, 55], and colossal efforts have been devoted to determining the gene expression patterns that control this timing [25, 26]. The development of single-cell strategies has provided in-depth knowledge of gene expression patterns categorized by tissue cell-type strata; however, population-centered changes remained previously unapproached (to the best of our knowledge).

Due to the general importance associated with fertility and assisted reproductive technologies, we also aimed to study the remodeling of cell populations during the WOI by developing a robust procedure to cluster cells and statistically evaluate changes in their abundance.

Our results prove our ability to determine changing cell populations during the WOI. The genes associated with these subpopulations denote their transcriptional transition to a different cellular state. Like cell cycle genes, the described behavior of WOI genes and their temporal expression patterns in different cell types might interfere with further data analysis, becoming a potential source of unwanted variation. Management and proceedings related to cell cycle genes have already been implemented as routine in well-established analysis pipelines [56, 57]. Their expression allows for the detection of cells in active division, but their removal is highly recommended for downstream steps such as trajectory inference analysis.

To achieve our primary goal, we implemented PAM as PAM-BS on the dataset of our interest. We evaluated the performance of PAM-BS by comparing it to a benchmark study by Duó et al [9]. The datasets available can be considered small or medium-sized, and the results obtained by applying PAM-BS, with an appropriate parameter selection, remained similar to those of the additional similar methods.

## Conclusions

From a technical point of view, this paper contributes to the field by describing a sound and well-tested clustering method, PAM, through our PAM-BS implementation to large sets of data (assemblies of tens or even hundreds of thousands of cells with the expression of thousands of genes) thanks to efficient memory use and automatic parallelization for the most widely available hardware (multicore processors). The complete workflow for analyzing scRNA-seq datasets involves choosing the normalization procedure, the distance/dissimilarity type, and the number of features (which, in this case, corresponds to the number of HVGs). Notably, such choices should be made for each dataset. Filtering by silhouette represents an optional available procedure that may guide the clustering method in some cases. In this bioinformatics context, clustering success must be evaluated by exploring the biological meaning of the obtained cell groups, as the final purpose is to capture insight from each dataset to answer different biological questions (e.g., to describe a set of cell subpopulations that carry out a unique biological process in the tissue under study). We captured transcriptional profiles associated with the principal cell compartments (the epithelium and stroma) and less-studied cell populations within the endometrium and the WOI context. These less-studied cell populations include the endothelium, perivascular cells, and distinct immune cell types. We detected cellular abundance changes in all main cell lineages entering the WOI using the proposed approach. We employed the *scellpam* package to fully understand the transcriptional landscape within the endometrium and the WOI, thus providing an alternative insight into the intricate molecular processes in these specific biological contexts. In summary, we contribute to both the fields of reproductive biology and computational biology; we support a better understanding of the dynamics of endometrial remodeling in reproductive biology and report a ready-to-use novel methodology for single-cell data analysis in computational biology.

### Supplementary Information

**Additional file 1**. Scellpam package installation and workflow. 

**Additional file 2**. Tables with additional comparisons for PAM-BS execution times. 

**Additional file 3**. Graphical and numerical comparisons among PAM-BS and other methods. 

**Additional file 4**. R code and results for the differential cell abundance analysis. 

## Data Availability

The datasets analyzed during the current study are available in the GEO and ArrayExpress repositories: scRNA-seq on human endometrium across the natural menstrual cycle; *N = 71,032* cells. NCBI GEO accession number GSE111976 [27]. scRNA-seq of endometrial superficial biopsies; *N = 100,307* cells. ArrayExpress accession number E-MTAB-10287 [31]. scRNA-seq of endometriosis; *N = 118,144* cells. NCBI GEO accession number GSE213216 [32]. No cell lines were used in this study.

## Abbreviations

ARI: Adjusted Rand index
HVG: Highly variable genes
PAM: Partitioning Around Medoids
PAM-BS: PAM with BUILD + SWAP
PCA: Principal Component Analysis
scRNA-seq: Single-cell RNA sequencing
t-SNE: T-distributed Stochastic Neighbor Embedding
WOI: Window of implantation

## Code availability

The package developed for and used in this study, *scellpam*, is freely available under GNU license from the CRAN repository: https://cran.r-project.org/web/packages/scellpam/index.html.
