# Supplementary material for: A cell abundance analysis based on efficient PAM clustering for a better understanding of the dynamics of endometrial remodelling
Source: BMC Bioinformatics. 2023 Nov 22;24:440. doi: 10.1186/s12859-023-05569-6 (PMC10664584; doi:10.1186/s12859-023-05569-6)
Supplement: Supplementary file 1 — Additional file 1. Scellpam package installation and workflow. [file 12859_2023_5569_MOESM1_ESM.pdf]

Supplementary Material 1

Domingo, J.; Kutsyr, O.; León, T.; Perez, R.; Ayala, G. and Rosón, B.

2023-11-12

Table of contents

Package instalation 1
Load packages 1
scellpam workflow 2
Step 0: set debug to get messages 2
Step 1: Data load/storage 2
Step 2: Write the data to scellpam binary format (what we have called `jmatrix`) 3
Step 4: Filtering genes 4
Step 5: Calculating the distance/dissimilarity matrices, applying PAM and calculation of silhouette 5

Package instalation

To be done once. `scellpam` is currently in CRAN and should be installed/compiled without problems.

```
# install.packages("scellpam")
```

Load packages

```
library(scellpam)
library(Seurat)
```

scellpam workflow

Step 0: set debug to get messages

Turn off/on informative messages from the package

```
# Debugging set to ON for biological part and for the dissimilarty
# calculation and PAM
ScellpamSetDebug(TRUE,TRUE)
```

Step 1: Data load/storage

Read c.10x data from S4 object created by `Seurat` package stored in a `.rds` file and write it to binary. The load of `Seurat` package is implicitly done by this load but the user can do it before.

```
q <- readRDS("c.10x.rds")
```

This function from the scellpam package gets the groups (different groups or samples) present inside the Seurat object q as `q@meta.data$orig.ident`.

WARNING: even we (the authors of scellpam) have tried to be compatible with `Seurat` as much as possible, this function might not work, depending on the internal organization of the Seurat object.

```
Gr <- GetSeuratGroups(q)
```

Find variable features to filter dataset by genes. This is done using the `Seurat` function but the user may choose another procedure.

```
q <- FindVariableFeatures(q, selection.method = "vst", nfeatures = 5000)
```

Identify 100, 500, 1000, 2000, 3000, 4000 and 5000 highly variable genes and store it in a list of seven elements.

```
vlchoices = c(100, 500, 1000, 2000, 3000, 4000, 5000)
variable_list <- list()
for (v in 1:length(vlchoices))
{
  variable_list[[v]] <- head(VariableFeatures(q), vlchoices[v])
```

```
}
```

All results are now accessible as `variable_list[[1]]` to `[[7]]` (or `variable_list$'100'`, etc.)

Step 2: Write the data to scellpam binary format (what we have called `jmatrix`)

Parameters:

- **`mtype`**: its default value is sparse, since it is generated from a matrix (`q@assays$RNA@counts`) that we know to be sparse. But you could store it in binary as full, it you want.

- **`ctype`**: can be set to `rawn` or to `log1n` for raw normalization or log(c+1) normalization.

- **`valuetype`**: its default value if 'float' but you can used double if you suspect yu need higher precision. To calculate dissimilarity matrix and later PAM clustering, float should be enough and used memory is reduced in half.

- **`transpose`**: the binary matrix must be transposed. This is a requirement to calculate dissimilarity matrix later.

- **`comment`**: use a sensible comment. It will help when you get information about the generated binary matrix.

- For raw counts normalization:

```
outname_base_rawn <- "Wang_rawn_trasp"
outname_all_rawn <-  paste0(outname_base_rawn, "_fall.bin")
dgCMatToJMat(
  q@assays$RNA@counts,
  outname_all_rawn,
  ctype = "rawn",
  transpose = TRUE,
  comment = "Wang data with all genes, normalized to sum of raw counts
   ↪  by cell. After normalization, matrix is transposed to have cells
   ↪  as rows and genes as columns"
)
```

- For log1 normalization:

```
outname_base_log1 <- "Wang_log1n_trasp"
outname_all_log1 <-  paste0(outname_base_log1, "_fall.bin")
dgCMatToJMat(
```

```
    q@assays$RNA@counts,
    outname_all_log1,
    ctype = "log1n",
    transpose = TRUE,
    comment = "Wang data with all genes, normalized to sum of log2 by
     ↪  cell. After normalization, matrix is transposed to have cells as
     ↪  rows and genes as columns"
)
```

To store in a text file or print in the console the information about a stored binary files:

```
JMatInfo(outname_all_rawn,
         paste0(outname_base_rawn, "_fall_Info.txt")) # rawn
JMatInfo(outname_all_log1,
         paste0(outname_base_log1, "_fall_Info.txt")) # log1n
```

If you execute simply `JMatInfo(outname_all_rawn)` you get the information in the console, namely:

- **File**: Wang_log1n_trasp_fall.bin

- **Matrix type**: SparseMatrix

- **Number of elements**: 1980585256

- **Data type**: float

- **Endianness**: little endian (same as this machine)

- **Number of rows**: 71032

- **Number of columns**: 27883

- **Metadata**: Stored names of rows and columns.

- **Metadata comment**: "Wang data with all genes, normalized to sum of log2 by cell. After normalization, matrix is transposed to have cells as rows and genes as columns"

- **Binary data size**: 1433012656 bytes, which is 18.0882% of the full matrix size (which would be 7922341024 bytes).

Last line show the advantage of having used sparse for this matrix.

Step 4: Filtering genes

Build seven filtered versions with appropriate file names:

```
fvals = c("100", "500", "1000", "2000", "3000", "4000", "5000")
```

- For raw counts normalization:

```
for (f in 1:length(fvals)) {
  outname_now <- paste0(outname_base_rawn, "_f", fvals[f], ".bin")
  # The list of genes to be kept is at variable_list[[1]] to [[7]].
  # The namesat value cols is because we transposed the matrix before
  # binary writing.
  FilterJMatByName(outname_all_rawn, variable_list[[f]], outname_now,
                   namesat = "cols")
}
```

- For log1 normalization:

```
for (f in 1:length(fvals)) {
  outname_now <- paste0(outname_base_log1, "_f", fvals[f], ".bin")
  # The list of genes to be kept is at variable_list[[1]] to [[7]].
  # The namesat value cols is because we transposed the matrix before
  # binary writing.
  FilterJMatByName(outname_all_log1, variable_list[[f]], outname_now,
                   namesat = "cols")
}
```

Step 5: Calculating the distance/dissimilarity matrices, applying PAM and calculation of silhouette

WARNING: this is calculated in two nested loops for all reasonable metrics/dissimilarities (Pearson, L1 and L2), for several possible numbers of genes (from 100 to all) and twice, one for the data with raw normalization and other for the data with log1 normalization, which is very slow. All results are kept in Rdata files to be analyzed to choose the best option, but you may have additional information that rules out some possibilities. In such case, cut the lists of possible_metrics and fvals.

```
options(nwarnings = 20000)
possible_metrics = c("Pearson", "L1", "L2")
```

The raw data now include also the file with all genes (no filtering).

```
fvals = c("100", "500", "1000", "2000", "3000", "4000", "5000", "all")
```

- For raw counts normalization:

```
for (met in 1:length(possible_metrics)) {
  for (f in 1:length(fvals)) {
    inname_now <- paste0(outname_base_rawn, "_f", fvals[f], ".bin")

    dt <- possible_metrics[met]

    outname_now <-
      paste0("Wang_rawn_f", fvals[f], "_dis", dt, ".bin")
    comment_now <-
      paste0(
        "Dissimilarity matrix with ",
        dt,
        " dissimilarity for Wang data rawcount-norm. and filtered
         ↪  keeping ",
        fvals[f],
        " genes."
      )

    # Calculating the distance/dissimilarity matrices.
    # Clear the warnings buffer.
    warning("First warning")
    assign("last.warning", NULL, envir = baseenv())

    # The parameters are count file name, distance file name,
    # type of distance/dissimiarity, comment to be stored in the
    # distance matrix and nthreads. Use of nthreads=0 (default value)
    # makes the program chooses the number of threads accordingly to
    # the number of cores in your machine.
    CalcAndWriteDissimilarityMatrix(
      inname_now,
      outname_now,
      distype = dt,
      comment = comment_now,
      nthreads = 0
    )

    # The warnings are stored in files, just in case.
```

```
    warnfile_now <-
      paste0("DissimilarityWarningslog1_", dt, "_", fvals[f], ".log")
    save(last.warning, file = warnfile_now, ascii = TRUE)

    # Apply PAM.
    L = ApplyPAM(
      outname_now,
      k = 30,
      init_method = "BUILD",
      max_iter = 1000,
      nthreads = 0
    )
    save(L, file = paste0("Wang_rawn_f", fvals[f], "_", dt, ".rda"))

    # Calculate and save abundance matrices.
    M = BuildAbundanceMatrix(L$clasif, Gr)
    save(M, file = paste0("Wang_rawn_f", fvals[f], "_", dt,
     ↪  "_inicl.Rdata"))

    # Calculate and save Silhouette of the cluster. This may help in
    # the choice of the best combination of parameters (normalization
    # mode, distance/dissimilarity used and number of genes kept).
    S = CalculateSilhouette(L$clasif, outname_now, nthreads = 0)
    save(S,
         file = paste0("Wang_rawn_f", fvals[f], "_", dt,
          ↪  "_silhouette.Rdata"))
  }
}
```

- For log1 normalization:

```
for (met in 1:length(possible_metrics)) {
  for (f in 1:length(fvals)) {
    inname_now <- paste0(outname_base_log1, "_f", fvals[f], ".bin")

    dt <- possible_metrics[met]

    outname_now <- paste0("Wang_log1n_f", fvals[f], "_dis", dt, ".bin")
    comment_now <-
      paste0(
        "Dissimilarity matrix with ",
```

```
            dt,
            " dissimilarity for Wang data log1-norm. and filtered keeping ",
            fvals[f],
            " genes."
          )

        # Calculating the distance/dissimilarity matrices.
        # Clear the warnings buffer.
        warning("First warning")
        assign("last.warning", NULL, envir = baseenv())
        CalcAndWriteDissimilarityMatrix(inname_now,
                                        outname_now,
                                        distype = dt,
                                        comment = comment_now)

        warnfile_now <-
          paste0("DissimilarityWarningslog1_", dt, "_", fvals[f], ".log")
        save(last.warning, file = warnfile_now, ascii = TRUE)

        # Apply PAM.
        L = ApplyPAM(
          outname_now,
          k = 30,
          init_method = "BUILD",
          max_iter = 1000,
          nthreads = 0
        )
        save(L, file = paste0("Wang_log1n_f", fvals[f], "_", dt, ".rda"))

        # Save abundance matrices.
        M = BuildAbundanceMatrix(L$clasif, Gr)
        save(M, file = paste0("Wang_log1n_f", fvals[f], "_", dt,
         ↪  "_inicl.Rdata"))

        # Calculating Silhouette.
        S = CalculateSilhouette(L$clasif, outname_now, nthreads = 0)
        save(S,
             file = paste0("Wang_log1n_f", fvals[f], "_", dt,
              ↪  "_silhouette.Rdata"))
      }
    }
```
