# Supplementary material for: A cell abundance analysis based on efficient PAM clustering for a better understanding of the dynamics of endometrial remodelling
Source: BMC Bioinformatics. 2023 Nov 22;24:440. doi: 10.1186/s12859-023-05569-6 (PMC10664584; doi:10.1186/s12859-023-05569-6)
Supplement: Supplementary file 2 — Additional file 2. Tables with additional comparisons for PAM-BS execution times. [file 12859_2023_5569_MOESM2_ESM.pdf]

Supplementary Material 2

Domingo, J.; Kutsyr, O.; Leon, T.; Perez, R.; Ayala, G. and Rosón, B.

30/08/2023

PAM-BS execution time comparison tables

Varying the number of cells and threads and comparison with package cluster

Table 1 presents the elapsed time in seconds for the entire execution of PAM-BS. This comprises initialization using the BUILD method and optimization until the minimum is obtained.

The individuals (cells) in the subsamples of the total set of cells were randomly selected and the same test sample was used for both packages, *cluster* and *scellpam*.

The cluster package was executed with the `pamonce=5` option, which the manual states as the fastest. This package cannot be applied to a sample size of $71,032$ due to the $65,535$ cell limit, as shown by the NA in the last row.

Table 1: Time in s for PAM-BS to complete execution.

Sample size	Package and number of threads					
	cluster serial	scellpam serial	scellpam 8 th	scellpam 32 th	scellpam 64 th	scellpam 128 th
8,879	14.2	12.2	2.8	1.1	0.7	0.6
17,758	60.7	52.0	9.6	3.2	2.2	2.1
35,516	289.8	271.8	49.8	16.8	13.9	17.0
71,032	NA	1764.8	560.6	210.6	197.1	255.2

In all cases and for both packages, the execution time increases with the sample size and generally decreases as the number of threads increases, with the exception of 64 and 128 threads. This is due to the fact that the tests were conducted on a machine with 64 cores, and the experiment involving 128 threads utilizes hyperthreading, which does not result in any significant improvement for $8,879$ and $17,758$ cells, and is even detrimental for $35,516$ and $71,032$. Hyperthreading is especially advantageous for programs with multiple input/output operations, but it confers no advantages for computationally intensive numerical calculations.

Varying the number of cells and medoids

Table 2 shows the time in seconds for the complete execution of PAM-BS using *scellpam* with 64 threads. As in the previous case, it includes initialization with the BUILD method and optimization to minimum using the same subsamples.

In this experiment, the number of medoids is tested for a range between 26 and 45.

Table 2: Time in s for the complete execution of PAM-BS with different number of medoids.

Number of medoids	Sample size			
	8,879	17,758	35,516	71,032
26	0.50	1.65	11.20	158.92
27	0.48	1.70	11.54	163.31
28	0.47	1.92	11.11	168.44
29	0.47	1.97	11.75	172.91
30	0.50	2.04	11.33	182.15
31	0.53	2.01	12.09	191.78
32	0.58	2.14	12.18	196.22
33	0.57	2.13	12.13	201.08
34	0.61	2.24	12.59	210.39
35	0.61	2.31	13.18	215.07
36	0.64	2.33	13.82	210.22
37	0.67	2.47	14.32	205.58
38	0.68	2.53	14.47	219.67
39	0.67	2.68	15.14	239.07
40	0.68	2.75	15.13	238.74
41	0.70	2.80	15.77	248.13
42	0.70	2.88	15.59	248.25
43	0.72	2.84	15.70	252.85
44	0.75	2.89	15.72	252.71
45	0.76	3.03	15.74	252.16

The time required for execution typically rises with the number of medoids, although this is not always true. The total time is determined by the number of iterations executed in the optimization phase and, as a result, is reliant on the starting medoids: a greater number of medoids may lead to a faster convergence by grouping the samples more tightly. As expected, the execution time increases in relation to sample size due to the larger loop limits in the algorithm.
