# Supplementary material for: A cell abundance analysis based on efficient PAM clustering for a better understanding of the dynamics of endometrial remodelling
Source: BMC Bioinformatics. 2023 Nov 22;24:440. doi: 10.1186/s12859-023-05569-6 (PMC10664584; doi:10.1186/s12859-023-05569-6)
Supplement: Supplementary file 3 — Additional file 3. Graphical and numerical comparisons among PAM-BS and other methods. [file 12859_2023_5569_MOESM3_ESM.pdf]

Supplementary Material 3

Domingo, J.; Kutsyr, O.; León, T.; Perez, R.; Ayala, G. and Rosón, B.

2023-11-13

Table of contents

Packages . . . 1
Data . . . 1
Functions . . . 5
Calculating ARI scores . . . 10
Results . . . 32
Supplementary Figure 1 . . . 33
Supplementary Figure 2 . . . 34
Supplementary Figure 3 . . . 35
Supplementary Figure 4 . . . 44

Packages

```
pacman::p_load(SingleCellExperiment, scellpam, DuoClustering2018, fossil,
    mclust, xtable, tidyverse, PMCMRplus, knitr, kableExtra)
```

Data

The following functions and R Scripts are useful for both a graphical and a numerical comparison among the performance of PAM-BS and the other methods considered in Duò, Robinson, and Soneson (2020) (in terms of the ARI).

To facilitate the reading of this document, let us briefly explain the structure of the objects that contain both the databases and the results of the clustering methods in the Duò and Soneson (2023) package.

Each data set data is given as a SingleCellExperiment object. For example, the description of `sce_filteredHVG10_Koh()` is: "Gene or TCC counts for a scRNA-seq data set from Koh et

al. (2016), consisting of in vitro cultured H7 embryonic stem cells (WiCell) and H7-derived downstream early mesoderm progenitors." `HVG10` stands for the gene filtering method used.

The authors have considered methods for reducing the number of genes provided as input to the clustering method. The method named `Expr10` retains 10% of the original number of genes (with a non-zero count in at least one cell) in the respective data sets. The selected genes correspond with the highest average expression (log-normalized count) value across all cells. For `HVG10` they retain only the most highly variable genes and they used `M3Drop` to model the drop-out rate of the genes as a function of the mean expression level.

We have renamed the data sets as follows:

```
# Filtering method: Expr10
## Kumar
KuE <- sce_filteredExpr10_Kumar()
## KumarTCC
KuTCCE <- sce_filteredExpr10_KumarTCC()
## Koh
KoE <- sce_filteredExpr10_Koh()
## KohTCC
KoTCCE <- sce_filteredExpr10_KohTCC()
## Trapnell
TrapE <- sce_filteredExpr10_Trapnell()
## TrapnellTCC
TrapTCCE <- sce_filteredExpr10_TrapnellTCC()
## Zheng
Z4eqE <- sce_filteredExpr10_Zhengmix4eq()
Z4uE <- sce_filteredExpr10_Zhengmix4uneq()
Z8uE <- sce_filteredExpr10_Zhengmix8eq()
## Sim Kumar
SiKu4easyE <- sce_filteredExpr10_SimKumar4easy()
SiKu4hE <- sce_filteredExpr10_SimKumar4hard()
SiKu8hE <- sce_filteredExpr10_SimKumar8hard()

# Filtering method: HVG10
## Kumar
KuH <- sce_filteredHVG10_Kumar()
## KumarTCC
KuTCCH <- sce_filteredHVG10_KumarTCC()
## Koh
KoH <- sce_filteredHVG10_Koh()
## KohTCC
KoTCCH <- sce_filteredHVG10_KohTCC()
```

```r
## Trapnell
TrapH <- sce_filteredHVG10_Trapnell()
## TrapnellTCC
TrapTCCH <- sce_filteredHVG10_TrapnellTCC()
## Zheng
Z4eqH <- sce_filteredHVG10_Zhengmix4eq()
Z4uH <- sce_filteredHVG10_Zhengmix4uneq()
Z8H <- sce_filteredHVG10_Zhengmix8eq()
## Sim Kumar
SiKu4easyH <- sce_filteredHVG10_SimKumar4easy()
SiKu4hH <- sce_filteredHVG10_SimKumar4hard()
SiKu8hH <- sce_filteredHVG10_SimKumar8hard()

# Filtering method : M3Drop10
## Kumar
KuM <- sce_filteredM3Drop10_Kumar()
## KumarTCC
KuTCCM <- sce_filteredM3Drop10_KumarTCC()
## Koh
KoM <- sce_filteredM3Drop10_Koh()
## KohTCC
KoTCCM <- sce_filteredM3Drop10_KohTCC()
## Trapnell
TrapM <- sce_filteredM3Drop10_Trapnell()
## TrapnellTCC
TrapTCCM <- sce_filteredM3Drop10_TrapnellTCC()
## Zheng
Z4eqM <- sce_filteredM3Drop10_Zhengmix4eq()
Z4uM <- sce_filteredM3Drop10_Zhengmix4uneq()
Z8M <- sce_filteredM3Drop10_Zhengmix8eq()
## Sim Kumar
SiKu4easyM <- sce_filteredM3Drop10_SimKumar4easy()
SiKu4hM <- sce_filteredM3Drop10_SimKumar4hard()
SiKu8hM <- sce_filteredM3Drop10_SimKumar8hard()
```

The objects we rename as `res1`, ..., `res36`, contain the clustering results from the performance evaluation of clustering methods for the scRNA-seq data.

In the R Documentation of the package, we find that: "The clustering results are provided as a `data.frame` object containing 10 variables (columns) named `dataset`, `method`, `cell`, `run`, `k`, `resolution`, `cluster`, `trueclass`, `est_k` and `elapsed`".

```
# CLUSTERING RESULTS
# Filtering method: Expr10
## Kumar
res1 <- clustering_summary_filteredExpr10_Kumar_v2()
## KumarTCC
res2 <- clustering_summary_filteredExpr10_KumarTCC_v2()
## Koh
res3 <- clustering_summary_filteredExpr10_Koh_v2()
## KohTCC
res4 <- clustering_summary_filteredExpr10_KohTCC_v2()
## Trapnell
res5 <- clustering_summary_filteredExpr10_Trapnell_v2()
## TrapnellTCC
res6 <- clustering_summary_filteredExpr10_TrapnellTCC_v2()
## Zheng
res7 <- clustering_summary_filteredExpr10_Zhengmix4eq_v2()
res8 <- clustering_summary_filteredExpr10_Zhengmix4uneq_v2()
res9 <- clustering_summary_filteredExpr10_Zhengmix8eq_v2()
##  Sim Kumar
res10 <- clustering_summary_filteredExpr10_SimKumar4easy_v2()
res11 <- clustering_summary_filteredExpr10_SimKumar4hard_v2()
res12 <- clustering_summary_filteredExpr10_SimKumar8hard_v2()

# Filtering method: HVG10
## Kumar
res13 <- clustering_summary_filteredHVG10_Kumar_v2()
## KumarTCC
res14 <- clustering_summary_filteredHVG10_KumarTCC_v2()
## Koh
res15 <- clustering_summary_filteredHVG10_Koh_v2()
## KohTCC
res16 <- clustering_summary_filteredHVG10_KohTCC_v2()
## Trapnell
res17 <- clustering_summary_filteredHVG10_Trapnell_v2()
## TrapnellTCC
res18 <- clustering_summary_filteredHVG10_TrapnellTCC_v2()
## Zheng
res19 <- clustering_summary_filteredHVG10_Zhengmix4eq_v2()
res20 <- clustering_summary_filteredHVG10_Zhengmix4uneq_v2()
res21 <- clustering_summary_filteredHVG10_Zhengmix8eq_v2()
## Sim Kumar
```

```r
res22 <- clustering_summary_filteredHVG10_SimKumar4easy_v2()
res23 <- clustering_summary_filteredHVG10_SimKumar4hard_v2()
res24 <- clustering_summary_filteredHVG10_SimKumar8hard_v2()

# Filtering method: M3Drop10
## Kumar
res25 <- clustering_summary_filteredM3Drop10_Kumar_v2()
## KumarTCC
res26 <- clustering_summary_filteredM3Drop10_KumarTCC_v2()
## Koh
res27 <- clustering_summary_filteredM3Drop10_Koh_v2()
## KohTCC
res28 <- clustering_summary_filteredM3Drop10_KohTCC_v2()
## Trapnell
res29 <- clustering_summary_filteredM3Drop10_Trapnell_v2()
## TrapnellTCC
res30 <- clustering_summary_filteredM3Drop10_TrapnellTCC_v2()
## Zheng
res31 <- clustering_summary_filteredM3Drop10_Zhengmix4eq_v2()
res32 <- clustering_summary_filteredM3Drop10_Zhengmix4uneq_v2()
res33 <- clustering_summary_filteredM3Drop10_Zhengmix8eq_v2()
## Sim Kumar
res34 <- clustering_summary_filteredM3Drop10_SimKumar4easy_v2()
res35 <- clustering_summary_filteredM3Drop10_SimKumar4hard_v2()
res36 <- clustering_summary_filteredM3Drop10_SimKumar8hard_v2()
```

Functions

The function `testPAMBUILDforDuo` allows to add the results obtained with `PAM-BS`to these data frames. In this way we can use the function `plot_performance()`in Duò and Soneson (2023) to generate a plot of the agreement between each partition of the cells and the one considered as true in terms of the adjusted Rand Index (ARI).

`testPAMBUILDforDuo()` only requires a distance/dissimilarity matrix, because `PAM-BS` runs very fast once this matrix is provided.

Depending on the choice of the normalization method (selected when the function `SceToJMat()` is called) and the metrics/dissimilarity in `CalcAndWriteDissimilarityMatrix()`, `PAM-BS` may return different partitions, therefore different data frames can be obtained.

```
#' testPAMBUILDforDuo
#'
#' Runs the PAM-BS algorithm from the scellpam package on a dataset of
#' a single cell experiment and creates the dataframe in the format used
#' by the DuoClustering2018 package so that the adjusted rand index (ARI)
#' scores can be obtained and comparative plots can be generated.
#'
#' @param   dsname     A string with the name of the dataset used
#' @param   dissfile   A string with the name of the dissimilarity/distance
#' matrix
#' @param   cellnames  A string obtained as colnames(sceobject)
#' @param   kvalues    A vector with the values of the number of clusters
#' to try successively
#' @param   celltclass A string with the labels of the true classes each
#' cell belongs to. It should be obtained from a sce object:
#' sceobject$phenoid.
#' @return  A dataframe with as many rows as (number of cells by number
#' of k values) and 10 columns, as described in the paper from Duó.
#' Namely:
#'          dataset: Name of the dataset, same for all cells and k values
#'          method:  In this case, BUILDLAB for all cells and k values
#'          cell:    The cell identifier. Each cell will appear (number
#'                   of different k values) times.
#'          run:     The number of run. In this case, 1 for all cells and
#'                   k values. This is because we use just one run, since
#'                   this method is deterministic and therefore all runs
#'                   would get the same result.
#'          k:       The number of clusters. Same for all cells in each
#'                   test of k-value.
#           resolution: NA for all cells and k values (only used for Seurat)
#'          cluster: Cluster assigned to each cell in each k value. A string.
#'          trueclass: Cluster to which each cell really belongs to. A
#'                   string.
#'          est_k:   NA for all cells and k values (we do not make an
#'                   estimation of the optimal number of clusters in this
#'                   function). Nevertheless, see vignette of package
#'                   scellpam on estimation based on cluster silhouette.
#'          elapsed: Time spent in the clustering process. Same for all cells
#'                   in each test of k-value.
#'
#' @seealso [scellpam::ApplyPAM] which is the main function called by this
```

```r
#' one.\cr
#'
#' @export
testPAMBUILDforDuo <- function(dsname,
                               dissfile,
                               cellnames,
                               kvalues,
                               celltclass)
  {
    if (!("scellpam" %in% (.packages()))) {
      library(scellpam)
    }
    numcells <- length(cellnames)
    numtc <- length(celltclass)
    if (numcells != numtc) {
      stop(
        "Number of elements in file of cell names ",
        cellnames,
        ", which is ",
        numcells,
        " and in file of true classification ",
        trueclass[i],
        ",
        which is ",
        numtc,
        ", are different."
      )
    }

    for (i in 1:length(kvalues)) {
      t1 <- Sys.time()
      L = ApplyPAM(dissfile, kvalues[i], init_method = "BUILD")
      t2 <- Sys.time()
      dt <- as.numeric(t2 - t1)

      df <- data.frame(
        rep(dsname, numcells),
        rep("PAM-BS", numcells),
        cellnames,
        as.integer(rep(1, numcells)),
        rep(kvalues[i], numcells),
```

```
        as.numeric(rep(NA, numcells)),
        as.character(L$clasif),
        celltclass,
        as.numeric(rep(NA, numcells)),
        rep(dt, numcells)
      )

      colnames(df) <-
        c("dataset", "method", "cell", "run", "k", "resolution", "cluster",
          "trueclass", "est_k", "elapsed"
        )
      if (i == 1) {
        testPAMBUILDforDuo <- df

      }  else  {
        testPAMBUILDforDuo <- rbind(testPAMBUILDforDuo, df)
      }
    }
    return(testPAMBUILDforDuo)
  }
```

The next function, named `DuoAnalysis`, obtains the distance matrices and the partitions using `PAM-BS` for the data sets in Duò and Soneson (2023) and returns the ARI scores.

```
#' Distance matrices and clustering with PAM-BS
#' @description
#' This function evaluates distance matrices and obtains clusters
#' for Duo datasets (the number of clusters is set as the number of
#' clusters of the "true partition". It returns a list containing the ARIs
#' to compare the partitions obtained by applying PAM-BS with the "true
#' partition".
#' @param datafun The name of the single cell experiment object to analyze
#' (see DuoClustering2018)
#' @param namefun Base name for output file
#' @param outputdir Directory to save
#' @param distypes Distances to be used
#' @param do.dist If TRUE the distance matrices are calculated
#' @param do.pam If TRUE PAM-BS is used to find clusters and ARIs
#' @export
#'
DuoAnalysis = function(datafun,
                       namefun,
```

```
                     outputdir,
                     distypes = c("Pearson", "L1", "L2"),
                     do.dist = FALSE,
                     do.pam = FALSE) {
resP = resL1 = resL2 = NA
cat("Reading data \n")
datafun
cat("Generating binary matrix\n")
M <- as.matrix(counts(datafun)) # genes filas, celulas columnas
SceToJMat(
  M,
  paste0(outputdir, namefun, ".bin"),
  rownames = rownames(datafun),
  colnames = colnames(datafun) ,
  mtype = "sparse",
  ctype = "rawn",
  transpose = TRUE
)
if (do.dist) {
  cat("Evaluating distances matrices\n")
  if (is.element("Pearson", distypes))
    CalcAndWriteDissimilarityMatrix(
      paste0(outputdir, namefun, ".bin"),
      paste0(outputdir, namefun, "P", ".bin"),
      distype = "Pearson",
      nthreads = 0
    )

  if (is.element("L1", distypes))
    CalcAndWriteDissimilarityMatrix(
      paste0(outputdir, namefun, ".bin"),
      paste0(outputdir, namefun, "L1", ".bin"),
      distype = "L1",
      nthreads = 0
    )
  if (is.element("L2", distypes))
    CalcAndWriteDissimilarityMatrix(
      paste0(outputdir, namefun, ".bin"),
      paste0(outputdir, namefun, "L2", ".bin"),
      distype = "L2",
      nthreads = 0
```

```
      )
    }
    if (do.pam) {
      if (is.element("Pearson", distypes)) {
        LP <- ApplyPAM(paste0(outputdir, namefun, "P", ".bin"),
                       k = length(table(datafun$phenoid)))
        resP = adj.rand.index(LP$clasif, as.numeric(factor(datafun$phenoid)))
      }
      if (is.element("L1", distypes)) {
        LP <- ApplyPAM(paste0(outputdir, namefun, "L1", ".bin"),
                       k = length(table(datafun$phenoid)))
        resL1 = adj.rand.index(LP$clasif, as.numeric(factor(datafun$phenoid)))
      }
      if (is.element("L2", distypes)) {
        LP <- ApplyPAM(paste0(outputdir, namefun, "L2", ".bin"),
                       k = length(table(datafun$phenoid)))
        resL2 = adj.rand.index(LP$clasif, as.numeric(factor(datafun$phenoid)))
      }
    }
    list(resL1 =  resL1,
         resP = resP,
         resL2 = resL2)
}

# Example
# DuoAnalysis(datafun = sce_filteredExpr10_Kumar(), namefun = "KuE",
# outputdir = "", distypes = c("L1", "Pearson", "L2"), do.dist = TRUE,
# do.pam = TRUE)
```

Calculating ARI scores

The calls can be read and executed sequentially with the following code.

```
myvarnames <- list()
myprepcalls <- list()
mymaincalls <- list()
mycleancalls <- list()

# FilteredExpr data sets
## Kumar
```

```
myvarnames[1] <- "KuE"
myprepcalls[1] <- expression(KuE <- sce_filteredExpr10_Kumar())
mymaincalls[1] <- expression(
  ret <- DuoAnalysis(
    datafun = KuE,
    namefun = "KuE",
    outputdir = "",
    distypes = c("L1", "Pearson", "L2"),
    do.dist = TRUE,
    do.pam = TRUE
  )
)
mycleancalls[1] <- expression(rm(KuE))

## KumarTCC
myvarnames[2] <- "KuTCCE"
myprepcalls[2] <- expression(KuTCCE <- sce_filteredExpr10_KumarTCC())
mymaincalls[2] <-
  expression(
    ret <- DuoAnalysis(
      datafun = KuTCCE,
      namefun = "KuTCCE",
      outputdir = "",
      distypes = c("L1", "Pearson", "L2"),
      do.dist = TRUE,
      do.pam = TRUE
    )
  )
mycleancalls[2] <- expression(rm(KuTCCE))

## Koh
myvarnames[3] <- "KoE"
myprepcalls[3] <- expression(KoE <- sce_filteredExpr10_Koh())
mymaincalls[3] <-
  expression(
    ret <- DuoAnalysis(
      datafun = KoE,
      namefun = "KoE",
      outputdir = "",
      distypes = c("L1", "Pearson", "L2"),
      do.dist = TRUE,
```

```
      do.pam = TRUE
    )
  )
mycleancalls[3] <- expression(rm(KoE))

## KohTCC
myvarnames[4] <- "KoTCCE"
myprepcalls[4] <- expression(KoTCCE <- sce_filteredExpr10_KohTCC())
mymaincalls[4] <-
  expression(
    ret <- DuoAnalysis(
      datafun = KoTCCE,
      namefun = "KoTCCE",
      outputdir = "",
      distypes = c("L1", "Pearson", "L2"),
      do.dist = TRUE,
      do.pam = TRUE
    )
  )
mycleancalls[4] <- expression(rm(KoTCCE))

## Trapnell
myvarnames[5] <- "TrapE"
myprepcalls[5] <- expression(TrapE <- sce_filteredExpr10_Trapnell())
mymaincalls[5] <-
  expression(
    ret <- DuoAnalysis(
      datafun = TrapE,
      namefun = "TrapE",
      outputdir = "",
      distypes = c("L1", "Pearson", "L2"),
      do.dist = TRUE,
      do.pam = TRUE
    )
  )
mycleancalls[5] <- expression(rm(TrapE))

## TrapnellTCC
myvarnames[6] <- "TrapTCCE"
myprepcalls[6] <-
  expression(TrapTCCE <- sce_filteredExpr10_TrapnellTCC())
```

```r
mymaincalls[6] <-
  expression(
    ret <- DuoAnalysis(
      datafun = TrapTCCE,
      namefun = "TrapTCCE",
      outputdir = "",
      distypes = c("L1", "Pearson", "L2"),
      do.dist = TRUE,
      do.pam = TRUE
    )
  )
mycleancalls[6] <- expression(rm(TrapTCCE))

## Zheng
myvarnames[7] <- "Z4eqE"
myprepcalls[7] <-
  expression(Z4eqE <- sce_filteredExpr10_Zhengmix4eq())
mymaincalls[7] <-
  expression(
    ret <- DuoAnalysis(
      datafun = Z4eqE,
      namefun = "Z4eqE",
      outputdir = "",
      distypes = c("L1", "Pearson", "L2"),
      do.dist = TRUE,
      do.pam = TRUE
    )
  )
mycleancalls[7] <- expression(rm(Z4eqE))

myvarnames[8] <- "Z4uE"
myprepcalls[8] <-
  expression(Z4uE <- sce_filteredExpr10_Zhengmix4uneq())
mymaincalls[8] <-
  expression(
    ret <- DuoAnalysis(
      datafun = Z4uE,
      namefun = "Z4uE",
      outputdir = "",
      distypes = c("L1", "Pearson", "L2"),
      do.dist = TRUE,
```

```
      do.pam = TRUE
    )
  )
mycleancalls[8] <- expression(rm(Z4uE))

myvarnames[9] <- "Z8uE"
myprepcalls[9] <- expression(Z8uE <- sce_filteredExpr10_Zhengmix8eq())
mymaincalls[9] <-
  expression(
    ret <- DuoAnalysis(
      datafun = Z8uE,
      namefun = "Z8uE",
      outputdir = "",
      distypes = c("L1", "Pearson", "L2"),
      do.dist = TRUE,
      do.pam = TRUE
    )
  )
mycleancalls[9] <- expression(rm(Z8uE))

## Sim Kumar
myvarnames[10] <- "SiKu4easyE"
myprepcalls[10] <-
  expression(SiKu4easyE <- sce_filteredExpr10_SimKumar4easy())
mymaincalls[10] <- expression(
  ret <- DuoAnalysis(
    datafun = SiKu4easyE,
    namefun = "SiKu4easyE",
    outputdir = "",
    distypes = c("L1", "Pearson", "L2"),
    do.dist = TRUE,
    do.pam = TRUE
  )
)
mycleancalls[10] <- expression(rm(SiKu4easyE))

myvarnames[11] <- "SiKu4hE"
myprepcalls[11] <-
  expression(SiKu4hE <- sce_filteredExpr10_SimKumar4hard())
mymaincalls[11] <-
  expression(
```

```
    ret <- DuoAnalysis(
      datafun = SiKu4hE,
      namefun = "SiKu4hE",
      outputdir = "",
      distypes = c("L1", "Pearson", "L2"),
      do.dist = TRUE,
      do.pam = TRUE
    )
  )
mycleancalls[11] <- expression(rm(SiKu4hE))

myvarnames[12] <- "SiKu8hE"
myprepcalls[12] <-
  expression(SiKu8hE <- sce_filteredExpr10_SimKumar8hard())
mymaincalls[12] <-
  expression(
    ret <- DuoAnalysis(
      datafun = SiKu8hE,
      namefun = "SiKu8hE",
      outputdir = "",
      distypes = c("L1", "Pearson", "L2"),
      do.dist = TRUE,
      do.pam = TRUE
    )
  )
mycleancalls[12] <- expression(rm(SiKu8hE))

# FilteredHVG data sets
## Kumar
myvarnames[13] <- "KuH"
myprepcalls[13] <- expression(KuH <- sce_filteredHVG10_Kumar())
mymaincalls[13] <-
  expression(
    ret <- DuoAnalysis(
      datafun = KuH,
      namefun = "KuH",
      outputdir = "",
      distypes = c("L1", "Pearson", "L2"),
      do.dist = TRUE,
      do.pam = TRUE
    )
```

```
  )
mycleancalls[13] <- expression(rm(KuH))

## KumarTCC
myvarnames[14] <- "KuTCCH"
myprepcalls[14] <- expression(KuTCCH <- sce_filteredHVG10_KumarTCC())
mymaincalls[14] <-
  expression(
    ret <- DuoAnalysis(
      datafun = KuTCCH,
      namefun = "KuTCCH",
      outputdir = "",
      distypes = c("L1", "Pearson", "L2"),
      do.dist = TRUE,
      do.pam = TRUE
    )
  )
mycleancalls[14] <- expression(rm(KuTCCH))

## Koh
myvarnames[15] <- "KoH"
myprepcalls[15] <- expression(KoH <- sce_filteredHVG10_Koh())
mymaincalls[15] <-
  expression(
    ret <- DuoAnalysis(
      datafun = KoH,
      namefun = "KoH",
      outputdir = "",
      distypes = c("L1", "Pearson", "L2"),
      do.dist = TRUE,
      do.pam = TRUE
    )
  )
mycleancalls[15] <- expression(rm(KoH))

## KohTCC
myvarnames[16] <- "KoTCCH"
myprepcalls[16] <- expression(KoTCCH <- sce_filteredHVG10_KohTCC())
mymaincalls[16] <-
  expression(
    ret <- DuoAnalysis(
```

```
      datafun = KoTCCH,
      namefun = "KoTCCH",
      outputdir = "",
      distypes = c("L1", "Pearson", "L2"),
      do.dist = TRUE,
      do.pam = TRUE
    )
  )
mycleancalls[16] <- expression(rm(KoTCCH))

## Trapnell
myvarnames[17] <- "TrapH"
myprepcalls[17] <- expression(TrapH <- sce_filteredHVG10_Trapnell())
mymaincalls[17] <-
  expression(
    ret <- DuoAnalysis(
      datafun = TrapH,
      namefun = "TrapH",
      outputdir = "",
      distypes = c("L1", "Pearson", "L2"),
      do.dist = TRUE,
      do.pam = TRUE
    )
  )
mycleancalls[17] <- expression(rm(TrapH))

## TrapnellTCC
myvarnames[18] <- "TrapTCCH"
myprepcalls[18] <-
  expression(TrapTCCH <- sce_filteredHVG10_TrapnellTCC())
mymaincalls[18] <-
  expression(
    ret <- DuoAnalysis(
      datafun = TrapTCCH,
      namefun = "TrapTCCH",
      outputdir = "",
      distypes = c("L1", "Pearson", "L2"),
      do.dist = TRUE,
      do.pam = TRUE
    )
  )
```

```r
mycleancalls[18] <- expression(rm(TrapTCCH))

## Zheng
myvarnames[19] <- "Z4eqH"
myprepcalls[19] <-
  expression(Z4eqH <- sce_filteredHVG10_Zhengmix4eq())
mymaincalls[19] <-
  expression(
    ret <- DuoAnalysis(
      datafun = Z4eqH,
      namefun = "Z4eqH",
      outputdir = "",
      distypes = c("L1", "Pearson", "L2"),
      do.dist = TRUE,
      do.pam = TRUE
    )
  )
mycleancalls[19] <- expression(rm(Z4eqH))

myvarnames[20] <- "Z4uH"
myprepcalls[20] <-
  expression(Z4uH <- sce_filteredHVG10_Zhengmix4uneq())
mymaincalls[20] <-
  expression(
    ret <- DuoAnalysis(
      datafun = Z4uH,
      namefun = "Z4uH",
      outputdir = "",
      distypes = c("L1", "Pearson", "L2"),
      do.dist = TRUE,
      do.pam = TRUE
    )
  )
mycleancalls[20] <- expression(rm(Z4uH))
myvarnames[21] <- "Z8H"
myprepcalls[21] <- expression(Z8H <- sce_filteredHVG10_Zhengmix8eq())
mymaincalls[21] <-
  expression(
    ret <- DuoAnalysis(
      datafun = Z8H,
      namefun = "Z8H",
```

```
      outputdir = "",
      distypes = c("L1", "Pearson", "L2"),
      do.dist = TRUE,
      do.pam = TRUE
    )
  )
mycleancalls[21] <- expression(rm(Z8H))

## Sim Kumar
myvarnames[22] <- "SiKu4easyH"
myprepcalls[22] <-
  expression(SiKu4easyH <- sce_filteredHVG10_SimKumar4easy())
mymaincalls[22] <- expression(
  ret <- DuoAnalysis(
    datafun = SiKu4easyH,
    namefun = "SiKu4easyH",
    outputdir = "",
    distypes = c("L1", "Pearson", "L2"),
    do.dist = TRUE,
    do.pam = TRUE
  )
)
mycleancalls[22] <- expression(rm(SiKu4easyH))

myvarnames[23] <- "SiKu4hH"
myprepcalls[23] <-
  expression(SiKu4hH <- sce_filteredHVG10_SimKumar4hard())
mymaincalls[23] <-
  expression(
    ret <- DuoAnalysis(
      datafun = SiKu4hH,
      namefun = "SiKu4hH",
      outputdir = "",
      distypes = c("L1", "Pearson", "L2"),
      do.dist = TRUE,
      do.pam = TRUE
    )
  )
mycleancalls[23] <- expression(rm(SiKu4hH))

myvarnames[24] <- "SiKu8hH"
```

```
myprepcalls[24] <-
  expression(SiKu8hH <- sce_filteredHVG10_SimKumar8hard())
mymaincalls[24] <-
  expression(
    ret <- DuoAnalysis(
      datafun = SiKu8hH,
      namefun = "SiKu8hH",
      outputdir = "",
      distypes = c("L1", "Pearson", "L2"),
      do.dist = TRUE,
      do.pam = TRUE
    )
  )
mycleancalls[24] <- expression(rm(SiKu8hH))

# FilteredM3Drop10 data sets
## Kumar
myvarnames[25] <- "KuM"
myprepcalls[25] <- expression(KuM <- sce_filteredM3Drop10_Kumar())
mymaincalls[25] <-
  expression(
    ret <- DuoAnalysis(
      datafun = KuM,
      namefun = "KuM",
      outputdir = "",
      distypes = c("L1", "Pearson", "L2"),
      do.dist = TRUE,
      do.pam = TRUE
    )
  )
mycleancalls[25] <- expression(rm(KuM))

## KumarTCC
myvarnames[26] <- "KuTCCM"
myprepcalls[26] <-
  expression(KuTCCM <- sce_filteredM3Drop10_KumarTCC())
mymaincalls[26] <-
  expression(
    ret <- DuoAnalysis(
      datafun = KuTCCM,
      namefun = "KuTCCM",
```

```
      outputdir = "",
      distypes = c("L1", "Pearson", "L2"),
      do.dist = TRUE,
      do.pam = TRUE
    )
  )
mycleancalls[26] <- expression(rm(KuTCCM))

## Koh
myvarnames[27] <- "KoM"
myprepcalls[27] <- expression(KoM <- sce_filteredM3Drop10_Koh())
mymaincalls[27] <-
  expression(
    ret <- DuoAnalysis(
      datafun = KoM,
      namefun = "KoM",
      outputdir = "",
      distypes = c("L1", "Pearson", "L2"),
      do.dist = TRUE,
      do.pam = TRUE
    )
  )
mycleancalls[27] <- expression(rm(KoM))

## KohTCC
myvarnames[28] <- "KoTCCM"
myprepcalls[28] <- expression(KoTCCM <- sce_filteredM3Drop10_KohTCC())
mymaincalls[28] <-
  expression(
    ret <- DuoAnalysis(
      datafun = KoTCCM,
      namefun = "KoTCCM",
      outputdir = "",
      distypes = c("L1", "Pearson", "L2"),
      do.dist = TRUE,
      do.pam = TRUE
    )
  )
mycleancalls[28] <- expression(rm(KoTCCM))

## Trapnell
```

```r
myvarnames[29] <- "TrapM"
myprepcalls[29] <-
  expression(TrapM <- sce_filteredM3Drop10_Trapnell())
mymaincalls[29] <-
  expression(
    ret <- DuoAnalysis(
      datafun = TrapM,
      namefun = "TrapM",
      outputdir = "",
      distypes = c("L1", "Pearson", "L2"),
      do.dist = TRUE,
      do.pam = TRUE
    )
  )
mycleancalls[29] <- expression(rm(TrapM))

## TrapnellTCC
myvarnames[30] <- "TrapTCCM"
myprepcalls[30] <-
  expression(TrapTCCM <- sce_filteredM3Drop10_TrapnellTCC())
mymaincalls[30] <-
  expression(
    ret <- DuoAnalysis(
      datafun = TrapTCCM,
      namefun = "TrapTCCM",
      outputdir = "",
      distypes = c("L1", "Pearson", "L2"),
      do.dist = TRUE,
      do.pam = TRUE
    )
  )
mycleancalls[30] <- expression(rm(TrapTCCM))

## Zheng
myvarnames[31] <- "Z4eqM"
myprepcalls[31] <-
  expression(Z4eqM <- sce_filteredM3Drop10_Zhengmix4eq())
mymaincalls[31] <-
  expression(
    ret <- DuoAnalysis(
      datafun = Z4eqM,
```

```
      namefun = "Z4eqM",
      outputdir = "",
      distypes = c("L1", "Pearson", "L2"),
      do.dist = TRUE,
      do.pam = TRUE
    )
  )
mycleancalls[31] <- expression(rm(Z4eqM))

myvarnames[32] <- "Z4uM"
myprepcalls[32] <-
  expression(Z4uM <- sce_filteredM3Drop10_Zhengmix4uneq())
mymaincalls[32] <-
  expression(
    ret <- DuoAnalysis(
      datafun = Z4uM,
      namefun = "Z4uM",
      outputdir = "",
      distypes = c("L1", "Pearson", "L2"),
      do.dist = TRUE,
      do.pam = TRUE
    )
  )
mycleancalls[32] <- expression(rm(Z4uM))
myvarnames[33] <- "Z8M"
myprepcalls[33] <-
  expression(Z8M <- sce_filteredM3Drop10_Zhengmix8eq())
mymaincalls[33] <-
  expression(
    ret <- DuoAnalysis(
      datafun = Z8M,
      namefun = "Z8M",
      outputdir = "",
      distypes = c("L1", "Pearson", "L2"),
      do.dist = TRUE,
      do.pam = TRUE
    )
  )
mycleancalls[33] <- expression(rm(Z8M))

## Sim Kumar
```

```
myvarnames[34] <- "SiKu4easyM"
myprepcalls[34] <-
  expression(SiKu4easyM <- sce_filteredM3Drop10_SimKumar4easy())
mymaincalls[34] <- expression(
  ret <- DuoAnalysis(
    datafun = SiKu4easyM,
    namefun = "SiKu4easyM",
    outputdir = "",
    distypes = c("L1", "Pearson", "L2"),
    do.dist = TRUE,
    do.pam = TRUE
  )
)
mycleancalls[34] <- expression(rm(SiKu4easyM))

myvarnames[35] <- "SiKu4hM"
myprepcalls[35] <-
  expression(SiKu4hM <- sce_filteredM3Drop10_SimKumar4hard())
mymaincalls[35] <-
  expression(
    ret <- DuoAnalysis(
      datafun = SiKu4hM,
      namefun = "SiKu4hM",
      outputdir = "",
      distypes = c("L1", "Pearson", "L2"),
      do.dist = TRUE,
      do.pam = TRUE
    )
  )
mycleancalls[35] <- expression(rm(SiKu4hM))

myvarnames[36] <- "SiKu8hM"
myprepcalls[36] <-
  expression(SiKu8hM <- sce_filteredM3Drop10_SimKumar8hard())
mymaincalls[36] <-
  expression(
    ret <- DuoAnalysis(
      datafun = SiKu8hM,
      namefun = "SiKu8hM",
      outputdir = "",
      distypes = c("L1", "Pearson", "L2"),
```

```
      do.dist = TRUE,
      do.pam = TRUE
    )
  )
mycleancalls[36] <- expression(rm(SiKu8hM))

##  full data sets
## Kumar
myvarnames[37] <- "KuF"
myprepcalls[37] <- expression(KuF <- sce_full_Kumar())
mymaincalls[37] <-
  expression(
    ret <- DuoAnalysis(
      datafun = KuF,
      namefun = "KuF",
      outputdir = "",
      distypes = c("L1", "Pearson", "L2"),
      do.dist = TRUE,
      do.pam = TRUE
    )
  )
mycleancalls[37] <- expression(rm(KuF))

## KumarTCC
myvarnames[38] <- "KuTCCF"
myprepcalls[38] <- expression(KuTCCF <- sce_full_KumarTCC())
mymaincalls[38] <-
  expression(
    ret <- DuoAnalysis(
      datafun = KuTCCF,
      namefun = "KuTCCF",
      outputdir = "",
      distypes = c("L1", "Pearson", "L2"),
      do.dist = TRUE,
      do.pam = TRUE
    )
  )
mycleancalls[38] <- expression(rm(KuTCCF))

## Koh
myvarnames[39] <- "KoF"
```

```r
myprepcalls[39] <- expression(KoF <- sce_full_Koh())
mymaincalls[39] <-
  expression(
    ret <- DuoAnalysis(
      datafun = KoF,
      namefun = "KoF",
      outputdir = "",
      distypes = c("L1", "Pearson", "L2"),
      do.dist = TRUE,
      do.pam = TRUE
    )
  )
mycleancalls[39] <- expression(rm(KoF))

## KohTCC
myvarnames[40] <- "KoTCCF"
myprepcalls[40] <- expression(KoTCCF <- sce_full_KohTCC())
mymaincalls[40] <-
  expression(
    ret <- DuoAnalysis(
      datafun = KoTCCF,
      namefun = "KoTCCF",
      outputdir = "",
      distypes = c("L1", "Pearson", "L2"),
      do.dist = TRUE,
      do.pam = TRUE
    )
  )
mycleancalls[40] <- expression(rm(KoTCCF))

## Trapnell
myvarnames[41] <- "TrapF"
myprepcalls[41] <- expression(TrapF <- sce_full_Trapnell())
mymaincalls[41] <-
  expression(
    ret <- DuoAnalysis(
      datafun = TrapF,
      namefun = "TrapF",
      outputdir = "",
      distypes = c("L1", "Pearson", "L2"),
      do.dist = TRUE,
```

```
      do.pam = TRUE
    )
  )
mycleancalls[41] <- expression(rm(TrapF))

## TrapnellTCC
myvarnames[42] <- "TrapTCCF"
myprepcalls[42] <- expression(TrapTCCF <- sce_full_TrapnellTCC())
mymaincalls[42] <-
  expression(
    ret <- DuoAnalysis(
      datafun = TrapTCCF,
      namefun = "TrapTCCF",
      outputdir = "",
      distypes = c("L1", "Pearson", "L2"),
      do.dist = TRUE,
      do.pam = TRUE
    )
  )
mycleancalls[42] <- expression(rm(TrapTCCF))

## Zheng
myvarnames[43] <- "Z4eqF"
myprepcalls[43] <- expression(Z4eqF <- sce_full_Zhengmix4eq())
mymaincalls[43] <-
  expression(
    ret <- DuoAnalysis(
      datafun = Z4eqF,
      namefun = "Z4eqF",
      outputdir = "",
      distypes = c("L1", "Pearson", "L2"),
      do.dist = TRUE,
      do.pam = TRUE
    )
  )
mycleancalls[43] <- expression(rm(Z4eqF))

myvarnames[44] <- "Z4uF"
myprepcalls[44] <- expression(Z4uF <- sce_full_Zhengmix4uneq())
mymaincalls[44] <-
  expression(
```

```
    ret <- DuoAnalysis(
      datafun = Z4uF,
      namefun = "Z4uF",
      outputdir = "",
      distypes = c("L1", "Pearson", "L2"),
      do.dist = TRUE,
      do.pam = TRUE
    )
  )
mycleancalls[44] <- expression(rm(Z4uF))

myvarnames[45] <- "Z8F"
myprepcalls[45] <- expression(Z8F <- sce_full_Zhengmix8eq())
mymaincalls[45] <-
  expression(
    ret <- DuoAnalysis(
      datafun = Z8F,
      namefun = "Z8F",
      outputdir = "",
      distypes = c("L1", "Pearson", "L2"),
      do.dist = TRUE,
      do.pam = TRUE
    )
  )
mycleancalls[45] <- expression(rm(Z8F))

## Sim Kumar
myvarnames[46] <- "SiKu4easyF"
myprepcalls[46] <- expression(SiKu4easyF <- sce_full_SimKumar4easy())
mymaincalls[46] <- expression(
  ret <- DuoAnalysis(
    datafun = SiKu4easyF,
    namefun = "SiKu4easyF",
    outputdir = "",
    distypes = c("L1", "Pearson", "L2"),
    do.dist = TRUE,
    do.pam = TRUE
  )
)
mycleancalls[46] <- expression(rm(SiKu4easyF))
```

```
myvarnames[47] <- "SiKu4hF"
myprepcalls[47] <- expression(SiKu4hF <- sce_full_SimKumar4hard())
mymaincalls[47] <-
  expression(
    ret <- DuoAnalysis(
      datafun = SiKu4hF,
      namefun = "SiKu4hF",
      outputdir = "",
      distypes = c("L1", "Pearson", "L2"),
      do.dist = TRUE,
      do.pam = TRUE
    )
  )
mycleancalls[47] <- expression(rm(SiKu4hF))

myvarnames[48] <- "SiKu8hF"
myprepcalls[48] <- expression(SiKu8hF <- sce_full_SimKumar8hard())
mymaincalls[48] <-
  expression(
    ret <- DuoAnalysis(
      datafun = SiKu8hF,
      namefun = "SiKu8hF",
      outputdir = "",
      distypes = c("L1", "Pearson", "L2"),
      do.dist = TRUE,
      do.pam = TRUE
    )
  )
mycleancalls[48] <- expression(rm(SiKu8hF))
```

The execution of the next chunk takes some time, therefore we provide the file `DuoARI.Rda` containing the results obtained from reading and executing sequentially the function `DuoAnalysis()` for all the data sets. The file is in the supplementary material folder.

```
rawnlist <- list()
for (i in 1:length(myvarnames)) {
  ## Loading distances matrices
  fsname <- paste0("./TEST_WITH_RAWN/", myvarnames[[i]], "_ret.Rda")
  load(fsname)
  rawnlist[myvarnames[[i]]] <- list(ret)
  rm(ret)
```

```
}

log1nlist <- list()
for (i in 1:length(myvarnames)) {
  ## Loading distances matrices
  fsname <- paste0("./TEST_WITH_LOG1N/", myvarnames[[i]], "_ret.Rda")
  load(fsname)
  log1nlist[myvarnames[[i]]] <- list(ret)
  rm(ret)
}
save(rawnlist, log1nlist, file = "DuoARI.Rda")
```

Next we load these results and for each `distype` ("Pearson","L1" or "L2") and normalization method ("log1n" or "rawn") we compare the ARI scores.

```
load("Supl/DuoARI.Rda")
```

```
# Accessing the results stored in DuoARI.Rda
dlogp = NULL
drawp = NULL
dlog1 = NULL
draw1 = NULL
dlog2 = NULL
draw2 = NULL
for (i in 1:length(myvarnames)) {
  dlogp[i] <- log1nlist[[myvarnames[[i]]]]$resP
  drawp[i] <- rawnlist[[myvarnames[[i]]]]$resP
  dlog1[i] <- log1nlist[[myvarnames[[i]]]]$resL1
  draw1[i] <- rawnlist[[myvarnames[[i]]]]$resL1
  dlog2[i] <- log1nlist[[myvarnames[[i]]]]$resL2
  draw2[i] <- rawnlist[[myvarnames[[i]]]]$resL2
}
```

As we have mentioned above, the functions `clustering_summary_..._v2()` in the package Duò and Soneson (2023) contain clustering results obtained by Duò *et al.*

The next function, called `duoARI1` retrieves the partitions obtained by the authors when the number of groups `k` is set to be equal to the number of subpopulations and calculates the median ARIs. It also recovers the ARIs obtained by using our method and performs a Friedman test and the pairwise comparisons.

More precisely, we have used the Friedman rank sum test to test for differences between the clustering methods and, for the post-hoc analysis, we have used the implementation by Eisinga

et al. Eisinga et al. (2017) to perform exact all pairs comparisons tests.

```
metodo=c("RaceID2", "FlowSOM", "CIDR", "TSCAN", "ascend", "PCAKmeans",
         "PCAHC", "SAFE", "pcaReduce", "RtsneKmeans",  "monocle", "SC3svm",
         "Seurat", "SC3")
banco=  c("KohTCC", "Koh", "KumarTCC", "Kumar", "SimKumar4easy",
          "SimKumar4hard", "SimKumar8hard", "TrapnellTCC", "Trapnell",
          "Zhengmix4eq", "Zhengmix4uneq", "Zhengmix8eq")
subp <- c(9, 9, 3, 3, 4, 4, 8, 3, 3, 4, 4, 8)
```

```
duoARI1 = function(sel, des, foutput) {
  result <- matrix(NA, nrow = 12, ncol = 14)
  for (i in 1:14) {
    for (j in 1:length(des)) {
      ar <- rep(0, 5)
      l <- vector("list", 5)
      tc <- vector("list", 5)
      for (r in 1:5) {
        l[[r]] <- des[[j]]$cluster[des[[j]]$method == metodo[i] &
                                   des[[j]]$run == r &
                                   des[[j]]$k == subp[j]]
        tc[[r]] <- des[[j]]$trueclass[des[[j]]$method == metodo[i] &
                                      des[[j]]$run == r &
                                      des[[j]]$k == subp[j]]
        ar[r] <- adjustedRandIndex(l[[r]], tc[[r]])
        result[j, i] <- round(median(ar, na.rm = TRUE), 2)
      }
    }
  }
  result <- t(result)
  colnames(result) = banco
  rownames(result) = metodo
  write.table(result, foutput, col.names = banco)

  # In order to compare the ARIs obtained by applying `PAM-BS` to
  # the ones obtained by the other methods we load our results.
  # `PB_lg1` refers to `PAM-BS` with logaritmic normalization and
  # distance L1, `PB_r1`  is `PAM-BS` with rawn normalization and
  # distance L1,  analogously  `PB_lg2`  (logarimic, L2),
  # `PB_r2`  (rawn L2), `PB_lgp` (logaritmic, Pearson), `PB_rp`
  # (rawn, Pearson).
```

```
  PB_lg1 <- round(dlog1[sel], 2)
  PB_r1 <- round(draw1[sel], 2)
  PB_lg2 <- round(dlog2[sel], 2)
  PB_r2 <- round(draw2[sel], 2)
  PB_lgp <- round(dlogp[sel], 2)
  PB_rp <- round(drawp[sel], 2)
  M <- rbind(PB_lg1, PB_r1, PB_lg2, PB_r2, PB_lgp, PB_rp, result)
  rownames(M) = c("PB_lg1", "PB_r1", "PB_lg2", "PB_r2", "PB_lgp", "PB_rp",
                  metodo)
  ARImean <- round(apply(M, 1, mean, na.rm = TRUE), 2)
  MM <- cbind(M, ARImean)
  MRED = na.omit(M)
  MRED <- data.frame(MRED)
  MRED$Methods <- rownames(MRED)
  Fri <- MRED %>%
    pivot_longer(!Methods,
                 names_to = "datasets",
                 values_to = "ARI") %>%  data.frame()
  fR.chi <- friedmanTest(Fri$ARI, Fri$Methods, Fri$datasets) # PMCMRplus
  ## friedman.test(Fri$ARI,Fri$Methods,Fri$datasets) #the same as fR.chi
  fR.Con <- friedmanTest(Fri$ARI, Fri$Methods, Fri$datasets,
                         method = "Fdist")
  fR.Con  # the same results than using PMCMRplus
  Mult_comp <- frdAllPairsExactTest(Fri$ARI, Fri$Methods, Fri$datasets,
                                    p.adjust.method = "BH")
  Mult_compBY <- frdAllPairsExactTest(Fri$ARI, Fri$Methods, Fri$datasets,
                                      p.adjust.method = "BY")
  M1 <- Mult_comp$p.value
  M1B <- round(Mult_compBY$p.value, 3)
  list(MM, fR.chi, fR.Con, M1B)
}
```

Results

Setting `distype= "Pearson"`, the next graph allows to compare the performance of `PAM-BS` depending on the normalization method chosen ("rawn" or "log1n").

```
summary(dlogp - drawp)
```

```
   Min.  1st Qu.   Median     Mean  3rd Qu.     Max.
```

```
-0.29479 -0.02631  0.02846  0.11263  0.27761  0.57921
```

Supplementary Figure 1

```
plot(
  dlogp - drawp,
  xlab = "data set",
  ylab = "arilog-ariraw",
  ylim = c(-0.6, 0.6),
  main = "Pearson"
)
abline(h = 0, col = "blue")
```

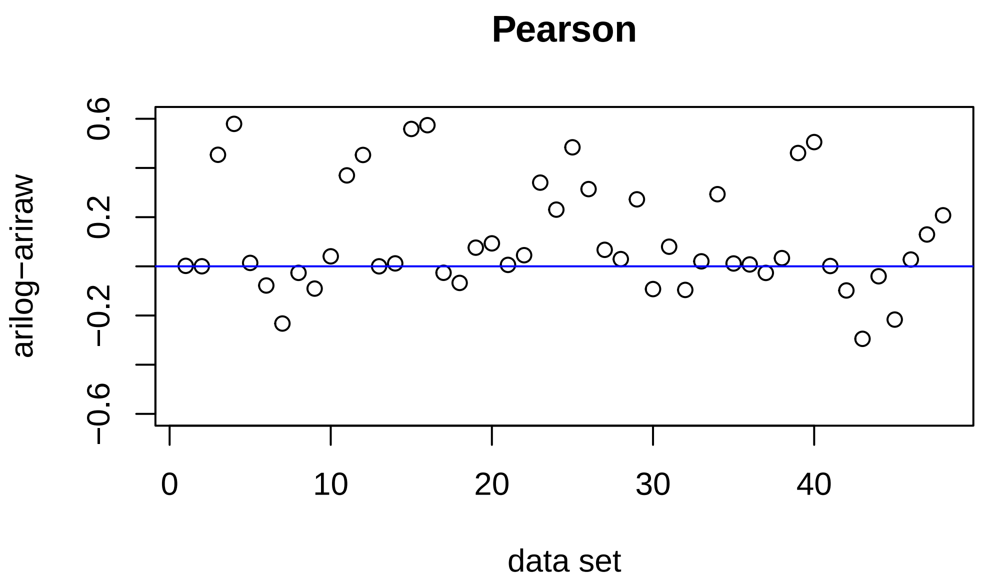

Figure 1: Supplementary Figure 1: Clustering using Pearson. Differences of ARI scores choosing log1n and rawn.

If `distype="Pearson"` the “log1n” normalization provides slightly better results.

Instead, if we set `distype="L1"` the best results are obtained using “rawn”.

```
summary(dlog1 - draw1)
```

```
     Min.   1st Qu.    Median      Mean   3rd Qu.      Max.
-0.251067 -0.097355 -0.024020 -0.030202  0.004936  0.386862
```

Supplementary Figure 2

```
plot(
  dlog1 - draw1,
  xlab = "data set",
  ylab = "arilog-ariraw",
  ylim = c(-0.6, 0.6),
  main = "L1"
)
abline(h = 0, col = "blue")
```

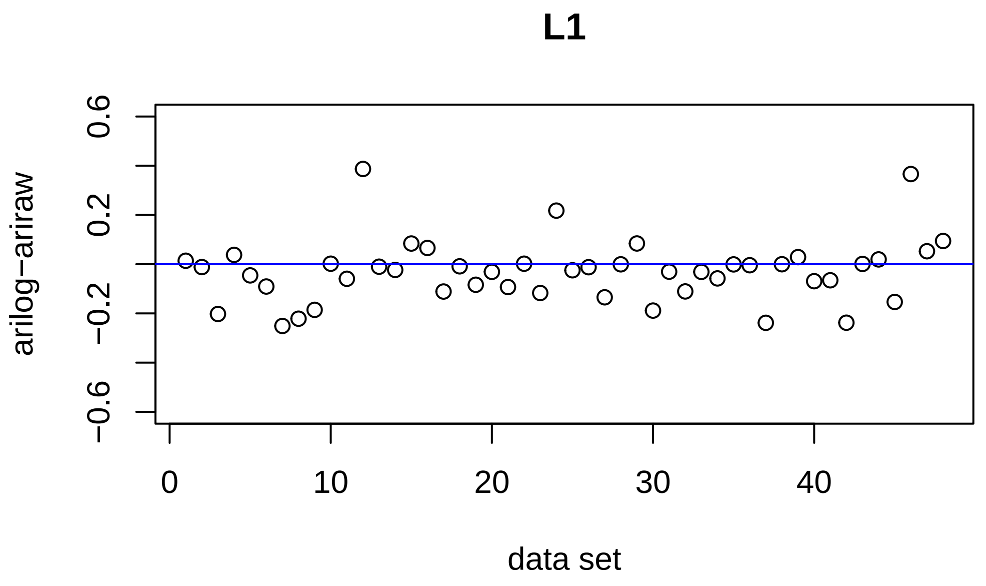

Figure 2: Supplementary Figure 2: Clustering using L1. Differences of ARI scores choosing log1n and rawn.

For `distype="L2"`, we also find better results using “rawn”:

```
summary(dlog2 - draw2)
```

```
    Min.   1st Qu.    Median      Mean   3rd Qu.      Max. 
-0.56160 -0.12915 -0.05943 -0.04478  0.06270  0.50638 
```

Supplementary Figure 3

```
plot(
  dlog2 - draw2,
  xlab = "data set",
  ylab = "arilog-ariraw",
  main = "L2",
  ylim = c(-0.6, 0.6)
)
abline(h = 0, col = "blue")
```

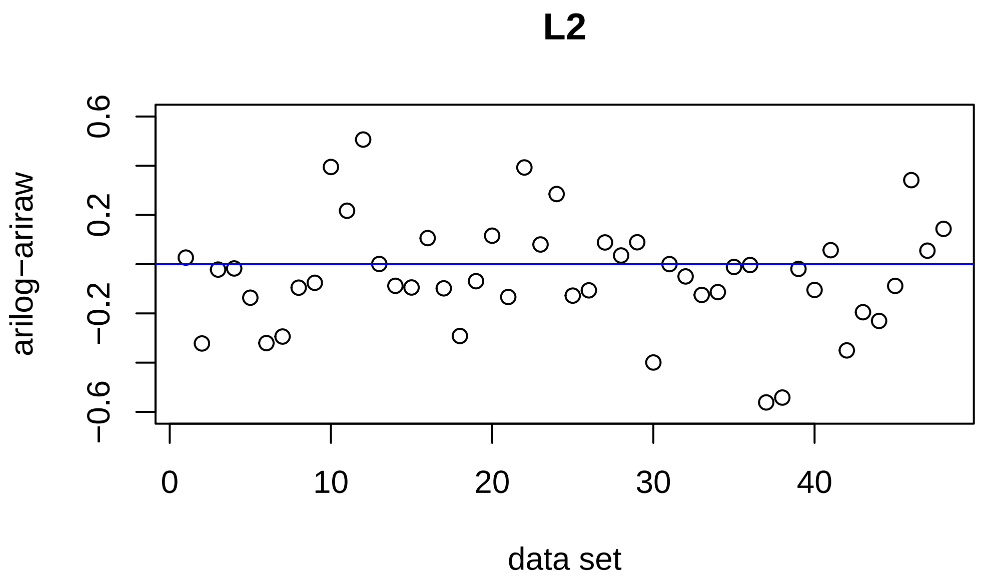

Figure 3: Supplementary Figure 3: Clustering using L2. Differences of ARI scores choosing log1n and rawn.

Next we compare the performance of the different methods analyzed in Duò and Soneson (2023) and `PAM-BS` (with different parameter selections). Each gene filtering method provides different data sets therefore we use `duoARI1`three times.

Firstly we compare the different clustering methods when the gene filtering methods is `Expr10`.

```
## Expr10
sel = c(4, 3, 2, 1, 10, 11, 12, 6, 5, 7, 8, 9)
des = vector("list", 12)
for (i in 1:12)
  des[[i]] = get(paste0("res", sel[i]))

da1 = duoARI1(sel = sel, des = des, foutput = 'ARI_DUO_Expr10.txt')
```

The observed ARI for all methods and data sets.

```
da1[[1]]  # Tables 1 and 2
```

Table 1: Expr10 ARI results for all methods and data sets (table 1 of 2).

	KohTCC	Koh	KumarTCC	Kumar	SimKumar4easy	SimKumar4hard	SimKumar8hard
PB_lg1	0.51	0.31	0.99	0.99	1.00	0.52	0.60
PB_r1	0.47	0.51	1.00	0.97	1.00	0.58	0.22
PB_lg2	0.16	0.19	0.67	0.99	1.00	0.52	0.57
PB_r2	0.18	0.21	0.99	0.96	0.61	0.31	0.06
PB_lgp	0.80	0.76	0.98	0.98	0.64	0.53	0.62
PB_rp	0.22	0.30	0.98	0.98	0.60	0.16	0.17
RaceID2	0.28	0.28	1.00	0.95	0.64	0.19	0.18
FlowSOM	0.85	0.70	0.56	0.51	1.00	1.00	1.00
CIDR	0.81	0.67	0.98	0.99	1.00	1.00	0.71
TSCAN	0.62	0.64	1.00	1.00	1.00	1.00	1.00
ascend	NA	NA	0.99	1.00	1.00	1.00	NA
PCAKmeans	0.90	0.84	0.98	0.99	1.00	1.00	1.00
PCAHC	0.89	0.87	1.00	1.00	1.00	1.00	1.00
SAFE	0.95	0.61	1.00	0.99	0.95	NA	NA
pcaReduce	0.98	0.94	1.00	1.00	1.00	1.00	0.70
RtsneKmeans	0.97	0.97	1.00	0.99	1.00	1.00	0.71
monocle	0.96	0.86	1.00	0.99	1.00	0.99	0.97
SC3svm	0.93	0.93	1.00	1.00	1.00	1.00	0.99
Seurat	0.90	0.86	0.99	0.99	1.00	1.00	1.00
SC3	0.94	0.94	1.00	1.00	1.00	1.00	1.00

We can see that some data sets as `Trapnel` and `TrapnellTCC` are harder to classify than others for all the methods.

The Friedman test is applied to compare the performance of the different methods.

```
da1[[2]]
```

```
	Friedman rank sum test

data:  y, groups and blocks
```

Table 2: Expr10 ARI results for all methods and data sets (table 2 of 2).

	TrapnellTCC	Trapnell	Zhengmix4eq	Zhengmix4uneq	Zhengmix8eq	ARImean
PB_lg1	0.27	0.28	0.58	0.54	0.35	0.58
PB_r1	0.36	0.32	0.83	0.77	0.54	0.63
PB_lg2	0.00	0.13	0.61	0.71	0.47	0.50
PB_r2	0.32	0.26	0.90	0.81	0.55	0.51
PB_lgp	0.26	0.33	0.66	0.59	0.50	0.64
PB_rp	0.34	0.32	0.90	0.61	0.59	0.51
RaceID2	0.24	0.28	0.77	0.66	0.33	0.48
FlowSOM	0.30	0.12	0.00	0.01	0.20	0.52
CIDR	0.28	0.34	0.31	0.48	0.25	0.65
TSCAN	0.18	0.22	0.62	0.60	0.42	0.69
ascend	0.40	0.39	0.30	0.49	0.40	0.66
PCAKmeans	0.30	0.36	0.28	0.44	0.37	0.70
PCAHC	0.39	0.42	0.27	0.48	0.35	0.72
SAFE	0.35	0.34	0.65	0.65	0.66	0.72
pcaReduce	0.36	0.33	0.66	0.64	0.55	0.76
RtsneKmeans	0.37	0.38	0.81	0.67	0.54	0.78
monocle	0.29	0.42	0.63	0.65	0.63	0.78
SC3svm	0.22	0.34	0.98	0.83	0.64	0.82
Seurat	0.38	0.36	0.98	0.98	0.72	0.85
SC3	0.44	0.45	0.97	0.83	0.66	0.85

```
Friedman chi-squared = 84.243, df = 17, p-value = 6.699e-11
```

In this case we have excluded the methods `ascend` and `SAFE` from the comparison because they have failed for some data sets (`KohTCC`, `Koh` and `SimKumar8hard`) to return a partition with the "true" number of groups.

Our conclusion is to reject the null hypothesis of no difference between methods.

The results of performing exact all pairs comparisons tests are:

```
da1[[4]]  # Tables 3 and 4
```

For $\alpha = 0.05$ we find that:

- `SC3` performs significantly better than `CIDR`, `FlowSOM`, `PB_lg1`, `PB_lg2`, `PB_lgp`, `PB_r2`, `PB_rp` and `RaceID2`.
- `Seurat` performs significantly better than `FlowSOM`, `PB_lg1`, `PB_lg2`, `PB_lgp`, `PB_rp` and `RaceID2`..
- `SC3svm` performs significantly better than `FlowSOM`, `PB_lg1`, `PB_lg2`, `PB_r2`, `PB_rp` and 'RaceID2".
- `RtsneKmeans` performs significantly better than `FlowSOM`, `PB_lg2` and `RaceID2`.
- `pcaReduce` performs significantly better than `PB_lg2`.

Table 3: Expr10 p-values of performing exact all pairs comparisons tests (table 1 of 2).

	CIDR	FlowSOM	monocle	PB_lg1	PB_lg2	PB_lgp	PB_r1	PB_r2	PB_rp
FlowSOM	1.00	NA	NA	NA	NA	NA	NA	NA	NA
monocle	0.92	0.22	NA	NA	NA	NA	NA	NA	NA
PB_lg1	1.00	1.00	0.25	NA	NA	NA	NA	NA	NA
PB_lg2	1.00	1.00	0.09	1.00	NA	NA	NA	NA	NA
PB_lgp	1.00	1.00	0.45	1.00	1.00	NA	NA	NA	NA
PB_r1	1.00	1.00	1.00	1.00	1.00	1.00	NA	NA	NA
PB_r2	1.00	1.00	0.27	1.00	1.00	1.00	1.00	NA	NA
PB_rp	1.00	1.00	0.25	1.00	1.00	1.00	1.00	1.00	NA
PCAHC	1.00	0.25	1.00	0.29	0.09	0.48	1.00	0.32	0.29
PCAKmeans	1.00	1.00	1.00	1.00	0.88	1.00	1.00	1.00	1.00
pcaReduce	0.42	0.08	1.00	0.09	0.03	0.18	1.00	0.09	0.09
RaceID2	1.00	1.00	0.09	1.00	1.00	1.00	1.00	1.00	1.00
RtsneKmeans	0.25	0.04	1.00	0.05	0.02	0.09	0.88	0.06	0.05
SC3	0.02	0.00	0.92	0.00	0.00	0.00	0.07	0.00	0.00
SC3svm	0.20	0.03	1.00	0.04	0.01	0.08	0.75	0.04	0.04
Seurat	0.10	0.02	1.00	0.02	0.01	0.04	0.45	0.03	0.02
TSCAN	1.00	1.00	1.00	1.00	0.98	1.00	1.00	1.00	1.00

Table 4: Expr10 p-values of performing exact all pairs comparisons tests (table 2 of 2).

	PCAHC	PCAKmeans	pcaReduce	RaceID2	RtsneKmeans	SC3	SC3svm	Seurat
FlowSOM	NA	NA	NA	NA	NA	NA	NA	NA
monocle	NA	NA	NA	NA	NA	NA	NA	NA
PB_lg1	NA	NA	NA	NA	NA	NA	NA	NA
PB_lg2	NA	NA	NA	NA	NA	NA	NA	NA
PB_lgp	NA	NA	NA	NA	NA	NA	NA	NA
PB_r1	NA	NA	NA	NA	NA	NA	NA	NA
PB_r2	NA	NA	NA	NA	NA	NA	NA	NA
PB_rp	NA	NA	NA	NA	NA	NA	NA	NA
PCAHC	NA	NA	NA	NA	NA	NA	NA	NA
PCAKmeans	1.00	NA	NA	NA	NA	NA	NA	NA
pcaReduce	1.00	1.00	NA	NA	NA	NA	NA	NA
RaceID2	0.10	0.92	0.03	NA	NA	NA	NA	NA
RtsneKmeans	1.00	0.98	1.00	0.02	NA	NA	NA	NA
SC3	0.86	0.09	1.00	0.00	1.00	NA	NA	NA
SC3svm	1.00	0.88	1.00	0.02	1.00	1.00	NA	NA
Seurat	1.00	0.57	1.00	0.01	1.00	1.00	1.0	NA
TSCAN	1.00	1.00	1.00	1.00	0.88	0.08	0.8	0.48

- We do not find any significant difference between `PAM-BS` with `normalization="rawn"` and `distance= "L1"` and `SC3`, `Seurat` or any other method.

Next, we perform the same analysis in case that the gene filtering method is `HVG10`.

```
## HVG10
sel = c(4, 3, 2, 1, 10, 11, 12, 6, 5, 7, 8, 9) + 12
des = vector("list", 12)
for (i in 1:12)
```

```
  des[[i]] = get(paste0("res", sel[i]))

da2 = duoARI1(sel = sel, des = des, foutput = 'ARI_DUO_HVG10.txt')
```

The observed ARI for all methods and data sets.

```
da2[[1]]  # Tables 5 and 6
```

Table 5: HVG10 ARI results for all methods and data sets (table 1 of 2).

	KohTCC	Koh	KumarTCC	Kumar	SimKumar4easy	SimKumar4hard	SimKumar8hard
PB_lg1	0.51	0.55	0.98	0.96	1.00	0.48	0.42
PB_r1	0.44	0.47	1.00	0.97	1.00	0.60	0.20
PB_lg2	0.29	0.17	0.89	0.96	1.00	0.38	0.35
PB_r2	0.18	0.26	0.98	0.96	0.61	0.30	0.06
PB_lgp	0.84	0.87	0.98	0.98	0.64	0.50	0.40
PB_rp	0.26	0.31	0.97	0.98	0.60	0.16	0.17
RaceID2	0.26	0.24	1.00	0.95	0.64	0.19	0.18
FlowSOM	0.83	0.87	0.99	0.99	1.00	1.00	0.99
CIDR	0.84	0.61	0.98	0.99	1.00	1.00	0.51
TSCAN	0.80	0.65	0.99	0.98	1.00	1.00	1.00
ascend	NA	NA	0.99	0.99	1.00	1.00	NA
PCAKmeans	0.89	0.91	0.98	0.99	1.00	1.00	1.00
PCAHC	0.93	0.90	1.00	0.99	1.00	0.99	1.00
SAFE	0.90	0.91	1.00	0.99	0.95	0.88	NA
pcaReduce	0.97	0.98	1.00	1.00	1.00	1.00	0.70
RtsneKmeans	0.96	0.99	1.00	0.99	1.00	1.00	0.70
monocle	0.94	0.98	0.85	0.97	1.00	1.00	0.99
SC3svm	0.93	0.93	1.00	1.00	1.00	1.00	0.98
Seurat	0.98	0.97	0.99	0.99	1.00	1.00	1.00
SC3	0.93	0.93	0.98	0.99	1.00	1.00	1.00

Again SAFE and ascend are excluded for the same reason.

```
da2[[2]]
```

```

	Friedman rank sum test

data:  y, groups and blocks
Friedman chi-squared = 57.363, df = 17, p-value = 2.843e-06
```

Our conclusion is to reject the null hypothesis of no difference between methods.

```
da2[[4]]  # Tables 7 and 8
```

For $\alpha = 0.05$ we find that:

- `SC3` performs significantly better than `RaceID2`.

Table 6: HVG10 ARI results for all methods and data sets (table 2 of 2).

	TrapnellTCC	Trapnell	Zhengmix4eq	Zhengmix4uneq	Zhengmix8eq	ARImean
PB_lg1	0.36	0.25	0.80	0.72	0.46	0.62
PB_r1	0.36	0.36	0.89	0.75	0.56	0.63
PB_lg2	0.05	0.22	0.82	0.79	0.41	0.53
PB_r2	0.34	0.31	0.89	0.67	0.54	0.51
PB_lgp	0.29	0.30	0.85	0.74	0.59	0.66
PB_rp	0.36	0.33	0.77	0.64	0.58	0.51
RaceID2	0.24	0.28	0.79	0.68	0.29	0.48
FlowSOM	0.30	0.29	0.00	0.01	0.14	0.62
CIDR	0.36	0.29	0.53	0.70	0.35	0.68
TSCAN	0.28	0.16	0.39	0.46	0.43	0.68
ascend	0.39	0.33	0.69	0.82	NA	0.78
PCAKmeans	0.28	0.31	0.31	0.20	0.23	0.68
PCAHC	0.29	0.31	0.31	0.40	0.30	0.70
SAFE	0.39	0.30	0.77	0.65	0.38	0.74
pcaReduce	0.29	0.31	0.68	0.65	0.44	0.75
RtsneKmeans	0.37	0.31	0.68	0.65	0.42	0.76
monocle	0.40	0.40	0.67	0.66	0.50	0.78
SC3svm	0.18	0.28	0.71	0.85	0.53	0.78
Seurat	0.38	0.40	0.95	0.97	0.70	0.86
SC3	0.53	0.45	0.71	0.84	0.49	0.82

Table 7: HVG10 p-values of performing exact all pairs comparisons tests (table 1 of 2).

	CIDR	FlowSOM	monocle	PB_lg1	PB_lg2	PB_lgp	PB_r1	PB_r2	PB_rp
FlowSOM	1.00	NA	NA	NA	NA	NA	NA	NA	NA
monocle	1.00	1.00	NA	NA	NA	NA	NA	NA	NA
PB_lg1	1.00	1.00	1.00	NA	NA	NA	NA	NA	NA
PB_lg2	1.00	1.00	0.38	1.00	NA	NA	NA	NA	NA
PB_lgp	1.00	1.00	1.00	1.00	1.00	NA	NA	NA	NA
PB_r1	1.00	1.00	1.00	1.00	0.66	1.00	NA	NA	NA
PB_r2	1.00	1.00	0.85	1.00	1.00	1.00	1.00	NA	NA
PB_rp	1.00	1.00	0.85	1.00	1.00	1.00	1.00	1.00	NA
PCAHC	1.00	1.00	1.00	1.00	1.00	1.00	1.00	1.00	1.00
PCAKmeans	1.00	1.00	1.00	1.00	1.00	1.00	1.00	1.00	1.00
pcaReduce	1.00	1.00	1.00	1.00	0.20	1.00	1.00	0.47	0.47
RaceID2	1.00	1.00	0.20	1.00	1.00	1.00	0.46	1.00	1.00
RtsneKmeans	1.00	1.00	1.00	1.00	0.16	1.00	1.00	0.46	0.46
SC3	1.00	0.47	1.00	0.46	0.06	1.00	1.00	0.12	0.12
SC3svm	1.00	1.00	1.00	1.00	0.20	1.00	1.00	0.47	0.47
Seurat	0.16	0.07	1.00	0.06	0.00	0.16	0.66	0.01	0.01
TSCAN	1.00	1.00	1.00	1.00	1.00	1.00	1.00	1.00	1.00

- `Seurat` performs significantly better than `PB_lg2`, `PB_r2`, `PB_rp` and `RaceID2`.
- We do not find any significant difference between `PB_lg1` or `PB_lgp` or `PB_r1` and `SC3`, `Seurat` or any other method.

And finally we perform the same analysis when the gene filtering method is `M3Drop`.

Table 8: HVG10 p-values of performing exact all pairs comparisons tests (table 2 of 2).

	PCAHC	PCAKmeans	pcaReduce	RaceID2	RtsneKmeans	SC3	SC3svm	Seurat
FlowSOM	NA	NA	NA	NA	NA	NA	NA	NA
monocle	NA	NA	NA	NA	NA	NA	NA	NA
PB__lg1	NA	NA	NA	NA	NA	NA	NA	NA
PB__lg2	NA	NA	NA	NA	NA	NA	NA	NA
PB__lgp	NA	NA	NA	NA	NA	NA	NA	NA
PB__r1	NA	NA	NA	NA	NA	NA	NA	NA
PB__r2	NA	NA	NA	NA	NA	NA	NA	NA
PB__rp	NA	NA	NA	NA	NA	NA	NA	NA
PCAHC	NA	NA	NA	NA	NA	NA	NA	NA
PCAKmeans	1.00	NA	NA	NA	NA	NA	NA	NA
pcaReduce	1.00	1.00	NA	NA	NA	NA	NA	NA
RaceID2	0.88	1.00	0.12	NA	NA	NA	NA	NA
RtsneKmeans	1.00	1.00	1.00	0.12	NA	NA	NA	NA
SC3	1.00	0.88	1.00	0.02	1	NA	NA	NA
SC3svm	1.00	1.00	1.00	0.12	1	1.00	NA	NA
Seurat	0.32	0.13	1.00	0.00	1	1.00	1	NA
TSCAN	1.00	1.00	1.00	1.00	1	0.47	1	0.07

```
## M3Drop
sel = c(4, 3, 2, 1, 10, 11, 12, 6, 5, 7, 8, 9) + 24
des = vector("list", 12)
for (i in 1:12)
  des[[i]] = get(paste0("res", sel[i]))

da3 = duoARI1(sel = sel, des = des, foutput = 'ARI_DUO_M3Drop.txt')
```

The observed ARI for all methods and data sets.

```
da3[[1]]  # Tables 9 and 10
```

As in Duò, Robinson, and Soneson (2020) (page 7) we observe that `M3Drop` filtering method ’ ’consistently led to a worse performance for the simulated data sets.

The Friedman test is applied to compare the performance of the different methods.

```
da3[[2]]
```

```

	Friedman rank sum test

data:  y, groups and blocks
Friedman chi-squared = 78.272, df = 17, p-value = 7.762e-10
```

And we perform exact all pairs comparisons tests.

Table 9: M3Drop10 ARI results for all methods and data sets (table 1 of 2).

	KohTCC	Koh	KumarTCC	Kumar	SimKumar4easy	SimKumar4hard	SimKumar8hard
PB_lg1	0.47	0.61	0.97	0.92	0.12	0.00	0.01
PB_r1	0.47	0.74	0.98	0.95	0.18	0.00	0.01
PB_lg2	0.23	0.19	0.53	0.58	0.06	0.01	0.00
PB_r2	0.19	0.10	0.64	0.71	0.17	0.02	0.01
PB_lgp	0.90	0.90	0.98	0.99	0.33	0.02	0.02
PB_rp	0.87	0.83	0.66	0.50	0.03	0.01	0.01
RaceID2	0.85	0.91	0.66	0.48	0.00	0.02	0.00
FlowSOM	0.91	0.92	0.99	1.00	0.86	0.10	0.00
CIDR	0.91	0.92	0.98	1.00	0.18	0.02	-0.01
TSCAN	0.74	0.73	0.99	1.00	0.72	0.15	0.03
ascend	NA	NA	0.98	0.99	0.57	NA	0.01
PCAKmeans	0.94	0.91	0.98	1.00	0.63	0.22	0.04
PCAHC	0.91	0.94	1.00	1.00	0.61	0.14	-0.01
SAFE	0.94	0.93	1.00	1.00	0.58	NA	NA
pcaReduce	0.94	0.92	1.00	1.00	0.63	0.15	0.06
RtsneKmeans	0.97	0.97	1.00	1.00	0.58	0.11	0.02
monocle	0.96	0.97	1.00	0.99	0.85	0.01	-0.01
SC3svm	0.93	0.93	1.00	1.00	0.63	0.13	-0.03
Seurat	0.96	0.97	0.99	1.00	0.62	0.10	0.07
SC3	0.92	0.98	1.00	1.00	0.95	0.32	0.07

Table 10: M3Drop10 ARI results for all methods and data sets (table 2 of 2).

	TrapnellTCC	Trapnell	Zhengmix4eq	Zhengmix4uneq	Zhengmix8eq	ARImean
PB_lg1	0.21	0.30	0.73	0.66	0.45	0.45
PB_r1	0.40	0.22	0.76	0.77	0.48	0.50
PB_lg2	0.00	0.10	0.59	0.68	0.23	0.27
PB_r2	0.40	0.01	0.59	0.73	0.35	0.33
PB_lgp	0.27	0.33	0.84	0.65	0.47	0.56
PB_rp	0.36	0.06	0.76	0.74	0.45	0.44
RaceID2	0.39	0.03	0.72	0.64	0.45	0.43
FlowSOM	0.25	0.31	0.00	0.01	0.08	0.45
CIDR	0.31	0.12	0.02	0.19	0.06	0.39
TSCAN	0.23	0.20	0.61	0.50	0.35	0.52
ascend	0.41	0.24	0.68	0.52	0.37	0.53
PCAKmeans	0.28	0.21	0.65	0.76	0.36	0.58
PCAHC	0.37	0.28	0.68	0.77	0.37	0.59
SAFE	0.29	0.30	0.06	0.73	0.14	0.60
pcaReduce	0.30	0.28	0.60	0.68	0.40	0.58
RtsneKmeans	0.34	0.34	0.94	0.66	0.49	0.62
monocle	0.30	0.39	0.93	0.67	0.33	0.62
SC3svm	0.15	0.15	0.95	0.77	0.57	0.60
Seurat	0.12	0.30	0.68	0.83	0.56	0.60
SC3	0.39	0.48	0.97	0.95	0.60	0.72

```
da3[[4]]  # Tables 11 and 12
```

For $\alpha = 0.05$, we find more significant differences in this case.

- `SC3` performs significantly better than `RaceID2`, `FlowSOM` and all of the six variants of

Table 11: M3Drop10 p-values of performing exact all pairs comparisons tests (table 1 of 2).

	CIDR	FlowSOM	monocle	PB_lg1	PB_lg2	PB_lgp	PB_r1	PB_r2	PB_rp
FlowSOM	1.00	NA	NA	NA	NA	NA	NA	NA	NA
monocle	0.68	1.00	NA	NA	NA	NA	NA	NA	NA
PB_lg1	1.00	1.00	0.50	NA	NA	NA	NA	NA	NA
PB_lg2	1.00	0.39	0.02	1.00	NA	NA	NA	NA	NA
PB_lgp	1.00	1.00	1.00	1.00	0.21	NA	NA	NA	NA
PB_r1	1.00	1.00	1.00	1.00	0.27	1.00	NA	NA	NA
PB_r2	1.00	1.00	0.30	1.00	1.00	1.00	1.00	NA	NA
PB_rp	1.00	1.00	0.95	1.00	1.00	1.00	1.00	1.00	NA
PCAHC	0.50	1.00	1.00	0.40	0.01	1.00	1.00	0.24	0.73
PCAKmeans	0.68	1.00	1.00	0.50	0.02	1.00	1.00	0.30	0.95
pcaReduce	0.32	1.00	1.00	0.27	0.01	1.00	1.00	0.14	0.50
RaceID2	1.00	1.00	0.50	1.00	1.00	1.00	1.00	1.00	1.00
RtsneKmeans	0.06	0.43	1.00	0.04	0.00	0.79	0.53	0.01	0.11
SC3	0.00	0.01	0.24	0.00	0.00	0.03	0.02	0.00	0.00
SC3svm	0.40	1.00	1.00	0.30	0.01	1.00	1.00	0.17	0.53
Seurat	0.17	0.85	1.00	0.12	0.00	1.00	1.00	0.05	0.26
TSCAN	1.00	1.00	1.00	1.00	0.32	1.00	1.00	1.00	1.00

Table 12: M3Drop10 p-values of performing exact all pairs comparisons tests (table 2 of 2).

	PCAHC	PCAKmeans	pcaReduce	RaceID2	RtsneKmeans	SC3	SC3svm	Seurat
FlowSOM	NA	NA	NA	NA	NA	NA	NA	NA
monocle	NA	NA	NA	NA	NA	NA	NA	NA
PB_lg1	NA	NA	NA	NA	NA	NA	NA	NA
PB_lg2	NA	NA	NA	NA	NA	NA	NA	NA
PB_lgp	NA	NA	NA	NA	NA	NA	NA	NA
PB_r1	NA	NA	NA	NA	NA	NA	NA	NA
PB_r2	NA	NA	NA	NA	NA	NA	NA	NA
PB_rp	NA	NA	NA	NA	NA	NA	NA	NA
PCAHC	NA	NA	NA	NA	NA	NA	NA	NA
PCAKmeans	1.0	NA	NA	NA	NA	NA	NA	NA
pcaReduce	1.0	1.00	NA	NA	NA	NA	NA	NA
RaceID2	0.4	0.50	0.26	NA	NA	NA	NA	NA
RtsneKmeans	1.0	1.00	1.00	0.04	NA	NA	NA	NA
SC3	0.3	0.24	0.46	0.00	1.00	NA	NA	NA
SC3svm	1.0	1.00	1.00	0.30	1.00	0.40	NA	NA
Seurat	1.0	1.00	1.00	0.12	1.00	0.90	1	NA
TSCAN	1.0	1.00	1.00	1.00	0.46	0.01	1	0.9

`PAM-BS`.

- `Seurat` performs significantly better than `PB_lg2` and `PB_r2`.
- `SC3svm` performs significantly better than `PB_lg2`.
- `monocle` performs significantly better than `PB_lg2`.
- `RtsneKmeans` performs significantly better than `PB_lg2` and `RaceID2`.
- `pcaReduce` performs significantly better than `PB_lg2`.
- `PCAHC` performs significantly better than `PB_lg2`.

- `PCAKmeans` performs significantly better than `PB_lg2`.

In this case, we can conclude that the variant of PAM-BS in which `normalization="log1n"` and `distance= "L2"` performs poorly.

As we have mentioned before, the function `testPAMBUILDforDuo()` can be used to obtain graphic representations of the ARI scores to compare `PAM-BS` with the other clustering methods.

For instance, the following chunk adds the results of `PAM-BS` ("L1", "rawn") to the data frame `clustering_summary_filteredExpr10_KohTCC_v2()` provided by Duò and Soneson (2023). The matrix "KoTCCEL1.bin" has been obtained using `DuoAnalysis()`.

```
# Example
## res4 KohTCC
B <- testPAMBUILDforDuo(
  dsname = res4$dataset[1],
  dissfile = paste0(dir, "KoTCCEL1.bin"),
  cellnames = colnames(KoTCCE),
  kvalues = seq(2, 10, 1),
  celltclass = KoTCCE$phenoid
)
resKoTCCE <- rbind(res9, B)
```

Supplementary Figure 4

We can represent the heatmap. The data frames have been previously obtained using `testPAMBUILDforDuo()`.

```
# Example: data sets filtered by Expr10
load("Supl/resKuE.Rda")
load("Supl/resKuTCCE.Rda")
load("Supl/resKoE.Rda")
load("Supl/resKoTCCE.Rda")
load("Supl/resTrapE.Rda")
load("Supl/resTrapTCCE.Rda")
load("Supl/resZ4eqE.Rda")
load("Supl/resZ4uE.Rda")
load("Supl/resZ8uE.Rda")
load("Supl/resSiKu4easyE.Rda")
load("Supl/resSiKu4hE.Rda")
load("Supl/resSiKu8hE.Rda")
```

```
perfB <- plot_performance(
  rbind(
    resZ4eqE,
    resZ4uE,
    resZ8uE,
    resKuE,
    resKuTCCE,
    resKoTCCE,
    resKoE,
    resTrapE,
    resTrapTCCE,
    resSiKu4easyE,
    resSiKu4hE,
    resSiKu8hE
  )
)
perfB$median_ari_heatmap_truek
```

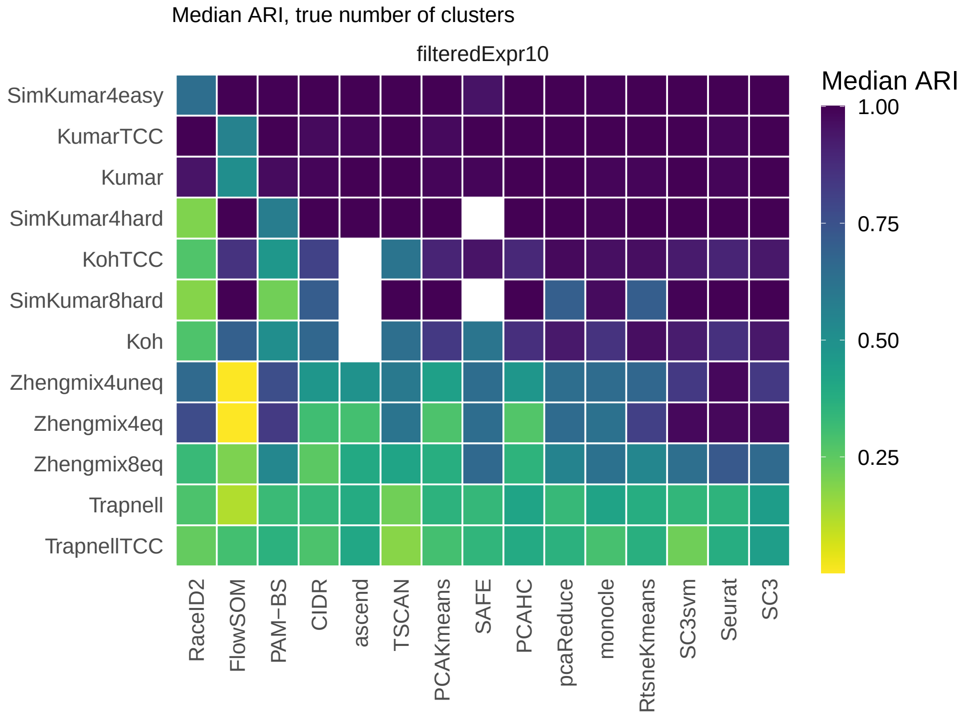

Figure 4: Supplementary Figure 4: Median ARI scores, to measure the agreement between the true partition and the one obtained by each method.

Duò, Angelo, Mark D. Robinson, and Charlotte Soneson. 2020. "A Systematic Performance Evaluation of Clustering Methods for Single-Cell RNA-Seq Data." *F1000Research* 7 (November): 1141. https://doi.org/10.12688/f1000research.15666.3.

Duò, Angelo, and Charlotte Soneson. 2023. *DuoClustering2018: Data, Clustering Results and Visualization Functions from Duò Et Al (2018).* https://doi.org/10.18129/B9.bioc.DuoClustering2018.

Eisinga, Rob, Tom Heskes, Ben Pelzer, and Manfred Te Grotenhuis. 2017. "Exact p-Values for Pairwise Comparison of Friedman Rank Sums, with Application to Comparing Classifiers." *BMC Bioinformatics* 18 (1): 68.

Koh, Pang Wei, Rahul Sinha, Amira A. Barkal, Rachel M. Morganti, Angela Chen, Irving L. Weissman, Lay Teng Ang, Anshul Kundaje, and Kyle M. Loh. 2016. "An Atlas of Transcriptional, Chromatin Accessibility, and Surface Marker Changes in Human Mesoderm Development." *Scientific Data* 3 (1). https://doi.org/10.1038/sdata.2016.109.
