# Supplementary material for: A cell abundance analysis based on efficient PAM clustering for a better understanding of the dynamics of endometrial remodelling
Source: BMC Bioinformatics. 2023 Nov 22;24:440. doi: 10.1186/s12859-023-05569-6 (PMC10664584; doi:10.1186/s12859-023-05569-6)
Supplement: Supplementary file 4 — Additional file 4. R code and results for the differential cell abundance analysis. [file 12859_2023_5569_MOESM4_ESM.pdf]

Supplementary Material 4

Domingo, J.; Kutsyr, O.; León, T.; Perez, R.; Ayala, G. and Rosón, B.

2023-11-13

Table of contents

Packages . . . 1
Functions . . . 1
Predictors . . . 7
Differential abundance . . . 7
Differential abundance with filtering . . . 17
Plots . . . 21
Multidimensional scaling over the medoids . . . 28

Packages

Loading packages needed later Maechler et al. (2022),Wickham et al. (2022).

```
pacman::p_load(scellpam, cluster, edgeR, ggplot2, ggfortify,
               knitr, kableExtra)
```

Functions

Some functions needed later. The function `DA()` performs a differential abundance analysis using the likelihood and the quasi-likelihood approaches proposed in McCarthy, Chen, and Smyth (2012) and Lund et al. (2012) respectively.

```
#' Differential abundance
#' @description
#' Differential abundance
#' @param abundance Abundance matrix
#' @param model_matrix Model matrix to analyzed
```

```r
#' @export
DA = function(abundance, model_matrix) {
  ## edgeR con GLM
  dge = edgeR::DGEList(counts = abundance)
  dge = estimateDisp(dge, design = model_matrix)
  ## edgeR with likelihood
  fit = glmFit(dge, design = model_matrix)
  ## edgeR with quasi-likelihood
  qlfit = glmQLFit(dge, design = model_matrix)
  list(abundance = abundance,
       fit = fit,
       qlfit = qlfit)
}
```

The following function `postDA()` analyzes the results obtained using the previous function `DA()`. It permits to test null coefficients or contrasts. The argument `filtering` provides the option to work instead of with the whole set of cells with the cells remaining after the filtering of those cells with low values of the silhouette.

```r
#' Analysis of DA results
#' @description
#' Analysis of DA results
#' @param hd Directory with DA outputs
#' @param filenames File names with the abundance matrices
#' @param dfilenames File with the description of \code{filenames}
#' @param model_matrix Model matrix of the linear model fitted
#' @param alpha False discovery rate
#' @param coefficient Coefficient(s) to be tested as null(s)
#' @param contrast Contrasts to evaluate
#' @param filtering Is there pruning using the silhouette?
postDA = function(hd,
                  filenames = paste0(hd, "names.txt"),
                  dfilenames = paste0(hd, "dnames.csv"),
                  model_matrix,
                  alpha = 0.05,
                  coefficient = NULL,
                  contrast = NULL,
                  filtering = FALSE) {
  res1 = res2 = sig1 = sig2 = NULL
  name0 = read.csv(filenames, header = FALSE)
  dname0 = read.table(dfilenames, sep = ";")
  abm = vector("list", nrow(name0))
```

```
names(abm) = name0[, 1]
for (i in 1:nrow(name0)) {
  load(paste0(hd, name0[i, 1]))
  if (filtering)
    abm[[i]] = DA(abundance = AbMfin, model_matrix = model_matrix)
  else
    abm[[i]] = DA(abundance = AbMini, model_matrix = model_matrix)
}

if (!is.null(coefficient)) {
  res1 = matrix(NA, nrow = length(abm), ncol = 4 + 2)
  res1 = as.data.frame(res1)
  names(res1) = c("db",
                  "normalization",
                  "ngenes",
                  "diss",
                  "sig.glm",
                  "sig.ql")
  sig1 = vector("list", length(abm))
  for (i in 1:length(abm)) {
    lrt1 = glmLRT(abm[[i]]$fit, coef = coefficient)
    a = topTags(lrt1, sort.by = "none", n = 100)
    p1 = a$table$PValue
    Ftest1 = glmQLFTest(abm[[i]]$qlfit, coef = coefficient)
    a = topTags(Ftest1, sort.by = "none", n = 100)[, "PValue"]
    p2 = a$table$PValue
    res1[i, 1:4] = dname0[i, 1:4]
    res1[i, 5] = sum(p.adjust(p1, method = "BH") <= alpha)
    res1[i, 6] =  sum(p.adjust(p2, method = "BH") <= alpha)
    sig1[[i]] = which(p.adjust(p1, method = "BH") <= alpha)
    sig2[[i]] = which(p.adjust(p2, method = "BH") <= alpha)
    names(sig1[i]) = names(sig2[i]) = names(abm)[i]
  }
  res1$sig.glm = as.numeric(res1$sig.glm)
  res1$sig.ql = as.numeric(res1$sig.ql)
}
if (!is.null(contrast)) {
  res2 = matrix(NA,
                nrow = length(abm),
                ncol = 4 + 2 * ncol(contrast))
  res2 = as.data.frame(res2)
```

```
  names(res2) = c(
    "db",
    "normalization",
    "ngenes",
    "diss",
    paste0("sig.glm", 1:ncol(contrast)),
    paste0("sig.ql", 1:ncol(contrast))
  )

  for (i in 1:length(abm)) {
    p1 = matrix(NA,
                nrow = nrow(abm[[i]]$abundance),
                ncol = ncol(contrast))
    for (j in 1:ncol(contrast)) {
      lrt1 = glmLRT(abm[[i]]$fit, contrast = contrast[, j])
      a = topTags(lrt1, sort.by = "none", n = 100)
      p1[, j] = a$table$PValue
    }
    p2 = matrix(NA,
                nrow = nrow(abm[[i]]$abundance),
                ncol = ncol(contrast))
    for (j in 1:ncol(contrast)) {
      lrt1 = glmQLFTest(abm[[i]]$qlfit, contrast = contrast[, j])
      a = topTags(lrt1, sort.by = "none", n = 100)
      p2[, j] = a$table$PValue
    }
    res2[i, 1:4] = dname0[i, 1:4]
    p11 = apply(p1, 2, function(y)
      sum(p.adjust(y, method = "BH") <= alpha))
    p22 = apply(p2, 2, function(y)
      sum(p.adjust(y, method = "BH") <= alpha))
    res2[i, 5:7] = p11
    res2[i, 8:10] = p22
  }
}
list(
  abm = abm,
  res1 = res1,
  sig1 = sig1,
  res2 = res2
)
```

```
}
```

The following function postDA2() analyzes the results obtained using the previous function DA(). It permits to test null coefficients or contrasts.

```
#' Analysis of DA results
#' @description
#' Analysis of DA results
#' @param filenames File names with the abundance matrices
#' @param model_matrix Model matrix of the linear model fitted
#' @param alpha False discovery rate
#' @param coefficient Coefficient(s) to be tested as null(s)
#' @param contrast Contrasts to evaluate
postDA2 = function(filenames,
                   model_matrix,
                   alpha = 0.05,
                   coefficient = NULL,
                   contrast = NULL) {
  res1 = res2 = sig1 = sig2 = NULL
  for (i in 1:length(filenames)) {
    load(filenames[i])
    abm = vector("list", length(ab))
    for (j in 1:length(ab))
      abm[[j]] = DA(abundance = ab[[j]]$abundance,
                    model_matrix = model_matrix)
  }

  if (!is.null(coefficient)) {
    res1 = matrix(NA, nrow = length(abm), ncol = length(ab[[1]]$pdata) + 2)
    sig1 = vector("list", length(abm))
    for (i in 1:length(abm)) {
      lrt1 = glmLRT(abm[[i]]$fit, coef = coefficient)
      a = topTags(lrt1, sort.by = "none", n = 100)
      p1 = a$table$PValue
      Ftest1 = glmQLFTest(abm[[i]]$qlfit, coef = coefficient)
      a = topTags(Ftest1, sort.by = "none", n = 100)[, "PValue"]
      p2 = a$table$PValue
      ff = ab[[i]]$pdata
      res1[i, ] =  c(ff,
                     sum(p.adjust(p1, method = "BH") <= alpha),
                     sum(p.adjust(p2, method = "BH") <= alpha))
```

```
    sig1[[i]] = list(pdata = ff, sig = which(p.adjust(p1, method = "BH") <=
                                              alpha))
    sig2[[i]] = list(pdata = ff, sig = which(p.adjust(p2, method = "BH") <=
                                              alpha))
    names(sig1[i]) = names(sig2[i]) = names(abm)[i]
  }
  res1 = as.data.frame(res1)
  names(res1) = c("diss",
                  "normalization",
                  "ngenes",
                  "state",
                  "nsig.glm",
                  "nsig.ql")
  res1$nsig.glm = as.numeric(res1$nsig.glm)
  res1$nsig.ql = as.numeric(res1$nsig.ql)

}
if (!is.null(contrast)) {
  res2 = matrix(NA,
                nrow = length(abm),
                ncol = length(ab[[1]]$pdata) + 2 * ncol(contrast))
  for (i in 1:length(abm)) {
    p1 = matrix(NA,
                nrow = nrow(abm[[i]]$abundance),
                ncol = ncol(contrast))
    for (j in 1:ncol(contrast)) {
      lrt1 = glmLRT(abm[[i]]$fit, contrast = contrast[, j])
      a = topTags(lrt1, sort.by = "none", n = 100)
      p1[, j] = a$table$PValue
    }
    p2 = matrix(NA,
                nrow = nrow(abm[[i]]$abundance),
                ncol = ncol(contrast))
    for (j in 1:ncol(contrast)) {
      lrt1 = glmQLFTest(abm[[i]]$qlfit, contrast = contrast[, j])
      a = topTags(lrt1, sort.by = "none", n = 100)
      p2[, j] = a$table$PValue
    }
    ff = ab[[i]]$pdata
    p11 = apply(p1, 2, function(y)
      sum(p.adjust(y, method = "BH") <= alpha))
```

```
      p22 = apply(p2, 2, function(y)
        sum(p.adjust(y, method = "BH") <= alpha))
      res2[i, ] =  c(ff, p11, p22)
    }
    res2 = as.data.frame(res2)
    res2[, (length(ab[[1]]$pdata) + 1):ncol(res2)] =
      sapply(res2[, (length(ab[[1]]$pdata) + 1):ncol(res2)], as.numeric)
    names(res2) = c(
      "diss",
      "normalization",
      "ngenes",
      "state",
      paste0("sig.glm", 1:ncol(contrast)),
      paste0("sig.ql", 1:ncol(contrast))
    )
    ## sig2
  }
  list(
    abm = abm,
    res1 = res1,
    sig1 = sig1,
    res2 = res2
  )
}
```

Predictors

Let us define the predictors used later: - `time` is the day of the menstrual cycle. - `time2` is binary variable indicating if the `time` is lesser or equal to the day 20 (0) or greater than 20 (1). The predictor `phase` is just the phase of the menstrual cycle. We will analyze each predictor separately.

```
time = c(17, 22, 22, 20, 23, 19, 24, 20, 26, 16)
time2 = cut(time, breaks = c(-Inf, 20, +Inf))
phase = factor(c(3, 4, 4, 4, 4, 4, 5, 4, 5, 3))
```

Differential abundance

This section contains the differential abundance analyses with the whole set of cells.

Set the directory where to found the abundance matrices.

```
dirAbundanceMatrices =
  "/home/gag/Nextcloud/GSE111976/data/ABUNDANCE_MATRICES/"
```

The following chunk will do the differential abundance analyses for two different normalizations (see details in the paper), different predictors (`time`, `time2` and `phase`) and different tests. The null hypothesis of a null coefficient is tested for the numeric variable `time` and the binary factor `time2`. If the predictor considered is the `phase` then the contrasts comparing each pair of levels of this variable are considered. Two different testing procedures are considered, the likelihood and the quasi-likelihood approaches proposed in McCarthy, Chen, and Smyth (2012) and Lund et al. (2012) respectively.

`time`

Differential abundance analyses with `time` predictor for two `raw` and `log` normalization:

```
model_matrix = model.matrix( ~ time)
df = postDA(
  hd = paste0(dirAbundanceMatrices, "ABM_NRAW/"),
  model_matrix = model_matrix,
  alpha = 0.05,
  coefficient = 2
)
```

```
df$res1  # Table 1
## df$sig1
```

```
save(df, file = paste0(
  paste0(dirAbundanceMatrices, "ABM_NRAW"),
  "_results_time.rda"
))
```

```
df = postDA(
  hd = paste0(dirAbundanceMatrices, "ABM_NLOG/"),
  model_matrix = model_matrix,
  alpha = 0.05,
  coefficient = 2
)
```

Table 1: The results correspond to model with predictor ‘time‘. Number of significant clusters using the likelihood approach (sig.glm) and quasi-likelihood (sig.ql) for database of study (db), ‘raw‘ normalization method (normalization), number of HVG (ngenes) and metric/dissimilarity (diss).

db	normalization	ngenes	diss	sig.glm	sig.ql
Wang	rawn	100	L1	2	2
Wang	rawn	100	L2	4	4
Wang	rawn	100	Pe	2	2
Wang	rawn	500	L1	2	0
Wang	rawn	500	L2	2	0
Wang	rawn	500	Pe	2	0
Wang	rawn	1000	L1	2	0
Wang	rawn	1000	L2	0	0
Wang	rawn	1000	Pe	0	0
Wang	rawn	2000	L1	0	0
Wang	rawn	2000	L2	2	0
Wang	rawn	2000	Pe	0	0
Wang	rawn	3000	L1	0	0
Wang	rawn	3000	L2	2	0
Wang	rawn	3000	Pe	0	0
Wang	rawn	4000	L1	0	0
Wang	rawn	4000	L2	2	0
Wang	rawn	4000	Pe	0	0
Wang	rawn	5000	L1	0	0
Wang	rawn	5000	L2	2	0
Wang	rawn	5000	Pe	0	0
Wang	rawn	20000	L1	0	0
Wang	rawn	20000	L2	0	0
Wang	rawn	20000	Pe	0	0

```
df$res1  # Table 2
## df$sig1
```

```
save(df, file = paste0(
  paste0(dirAbundanceMatrices, "ABM_NLOG"),
  "_results_time.rda"
))
```

`time2`

Differential abundance analyses with `time2` predictor for two `raw` and `log` normalization:

Table 2: The results correspond to model with predictor ‘time‘. Number of significant clusters using the likelihood approach (sig.glm) and quasi-likelihood (sig.ql) for database of study (db), ‘log‘ normalization method (normalization), number of HVG (ngenes) and metric/dissimilarity (diss).

db	normalization	ngenes	diss	sig.glm	sig.ql
Wang	lg1n	100	L1	0	0
Wang	lg1n	100	L2	1	0
Wang	lg1n	100	Pe	0	0
Wang	lg1n	500	L1	0	0
Wang	lg1n	500	L2	0	0
Wang	lg1n	500	Pe	0	0
Wang	lg1n	1000	L1	1	0
Wang	lg1n	1000	L2	0	0
Wang	lg1n	1000	Pe	0	0
Wang	lg1n	2000	L1	0	0
Wang	lg1n	2000	L2	0	0
Wang	lg1n	2000	Pe	0	0
Wang	lg1n	3000	L1	0	0
Wang	lg1n	3000	L2	0	0
Wang	lg1n	3000	Pe	0	0
Wang	lg1n	4000	L1	0	0
Wang	lg1n	4000	L2	0	0
Wang	lg1n	4000	Pe	0	0
Wang	lg1n	5000	L1	0	0
Wang	lg1n	5000	L2	0	0
Wang	lg1n	5000	Pe	0	0
Wang	lg1n	20000	L1	0	0
Wang	lg1n	20000	L2	0	0
Wang	lg1n	20000	Pe	0	0

```
model_matrix = model.matrix( ~ time2)
df = postDA(
  hd = paste0(dirAbundanceMatrices, "ABM_NRAW/"),
  model_matrix = model_matrix,
  alpha = 0.05,
  coefficient = 2
)
```

```
df$res1  # Table 3
## df$sig1
```

```
save(df, file = paste0(
  paste0(dirAbundanceMatrices, "ABM_NRAW"),
```

Table 3: The results correspond to model with predictor ‘time2‘. Number of significant clusters using the likelihood approach (sig.glm) and quasi-likelihood (sig.ql) for database of study (db), ‘raw‘ normalization method (normalization), number of HVG (ngenes) and metric/dissimilarity (diss).

db	normalization	ngenes	diss	sig.glm	sig.ql
Wang	rawn	100	L1	9	0
Wang	rawn	100	L2	9	0
Wang	rawn	100	Pe	3	0
Wang	rawn	500	L1	5	0
Wang	rawn	500	L2	5	0
Wang	rawn	500	Pe	0	0
Wang	rawn	1000	L1	0	0
Wang	rawn	1000	L2	3	0
Wang	rawn	1000	Pe	3	0
Wang	rawn	2000	L1	0	0
Wang	rawn	2000	L2	5	0
Wang	rawn	2000	Pe	0	0
Wang	rawn	3000	L1	0	0
Wang	rawn	3000	L2	5	0
Wang	rawn	3000	Pe	1	0
Wang	rawn	4000	L1	0	0
Wang	rawn	4000	L2	5	0
Wang	rawn	4000	Pe	1	0
Wang	rawn	5000	L1	0	0
Wang	rawn	5000	L2	5	0
Wang	rawn	5000	Pe	1	0
Wang	rawn	20000	L1	0	0
Wang	rawn	20000	L2	1	0
Wang	rawn	20000	Pe	0	0

```
  "_results_time2.rda"
))
```

```
df = postDA(
  hd = paste0(dirAbundanceMatrices, "ABM_NLOG/"),
  model_matrix = model_matrix,
  alpha = 0.05,
  coefficient = 2
)
```

```
df$res1  # Table 4
## df$sig1
```

Table 4: The results correspond to model with predictor 'time2'. Number of significant clusters using the likelihood approach (sig.glm) and quasi-likelihood (sig.ql) for database of study (db), 'log' normalization method (normalization), number of HVG (ngenes) and metric/dissimilarity (diss).

db	normalization	ngenes	diss	sig.glm	sig.ql
Wang	lg1n	100	L1	6	0
Wang	lg1n	100	L2	7	1
Wang	lg1n	100	Pe	5	0
Wang	lg1n	500	L1	2	1
Wang	lg1n	500	L2	1	0
Wang	lg1n	500	Pe	0	0
Wang	lg1n	1000	L1	0	0
Wang	lg1n	1000	L2	0	0
Wang	lg1n	1000	Pe	0	0
Wang	lg1n	2000	L1	0	0
Wang	lg1n	2000	L2	0	0
Wang	lg1n	2000	Pe	0	0
Wang	lg1n	3000	L1	0	0
Wang	lg1n	3000	L2	7	0
Wang	lg1n	3000	Pe	0	0
Wang	lg1n	4000	L1	0	0
Wang	lg1n	4000	L2	4	0
Wang	lg1n	4000	Pe	0	0
Wang	lg1n	5000	L1	0	0
Wang	lg1n	5000	L2	0	0
Wang	lg1n	5000	Pe	0	0
Wang	lg1n	20000	L1	0	0
Wang	lg1n	20000	L2	0	0
Wang	lg1n	20000	Pe	0	0

```
save(df, file = paste0(
  paste0(dirAbundanceMatrices, "ABM_NLOG"),
  "_results_time2.rda"
))
```

phase

Differential abundance analyses with `phase` predictor for two `raw` and `log` normalization:

```
model_matrix = model.matrix( ~ 0 + phase)
df = postDA(
  hd = paste0(dirAbundanceMatrices, "ABM_NRAW/"),
  model_matrix = model_matrix,
```

```
  alpha = 0.05,
  coefficient = 2:3
)
```

```
df$res1  # Table 5
## df$sig1
```

Table 5: The results correspond to model with predictor ‘phase‘. Number of significant clusters using the likelihood approach (sig.glm) and quasi-likelihood (sig.ql) for database of study (db), ‘raw‘ normalization method (normalization), number of HVG (ngenes) and metric/dissimilarity (diss).

db	normalization	ngenes	diss	sig.glm	sig.ql
Wang	rawn	100	L1	28	5
Wang	rawn	100	L2	27	4
Wang	rawn	100	Pe	27	22
Wang	rawn	500	L1	29	23
Wang	rawn	500	L2	30	29
Wang	rawn	500	Pe	30	28
Wang	rawn	1000	L1	30	21
Wang	rawn	1000	L2	30	29
Wang	rawn	1000	Pe	30	27
Wang	rawn	2000	L1	30	26
Wang	rawn	2000	L2	30	29
Wang	rawn	2000	Pe	29	27
Wang	rawn	3000	L1	30	27
Wang	rawn	3000	L2	30	29
Wang	rawn	3000	Pe	29	27
Wang	rawn	4000	L1	30	27
Wang	rawn	4000	L2	30	29
Wang	rawn	4000	Pe	30	28
Wang	rawn	5000	L1	29	24
Wang	rawn	5000	L2	30	29
Wang	rawn	5000	Pe	29	26
Wang	rawn	20000	L1	30	29
Wang	rawn	20000	L2	29	27
Wang	rawn	20000	Pe	30	28

```
save(df,
     file = paste0(dirAbundanceMatrices, "ABM_NRAW",
                   "_results_phase1.rda"))
```

```
contrast = makeContrasts(
  c1 = phase3 - phase4,
```

```
  c2 = phase3 - phase5,
  c3 = phase4 - phase5,
  levels = model_matrix
)
df = postDA(
  hd = paste0(dirAbundanceMatrices, "ABM_NRAW/"),
  model_matrix = model_matrix,
  alpha = 0.05,
  contrast = contrast
)
```

```
df$res2  # Table 6
```

Table 6: The results correspond to model with predictor ‘phase’ and tests the different contrasts comparing each pair of phases. Number of significant clusters using the likelihood approach (sig.glm1-3) and quasi-likelihood (sig.ql1-3) for database of study (db), ‘raw‘ normalization method (normalization), number of HVG (ngenes) and metric/dissimilarity (diss).

db	normalization	ngenes	diss	sig.glm1	sig.glm2	sig.glm3	sig.ql1	sig.ql2	sig.ql3
Wang	rawn	100	L1	0	0	2	0	0	1
Wang	rawn	100	L2	0	0	5	0	0	0
Wang	rawn	100	Pe	0	0	0	0	0	0
Wang	rawn	500	L1	0	0	2	0	0	0
Wang	rawn	500	L2	0	0	2	0	0	0
Wang	rawn	500	Pe	0	0	0	0	0	0
Wang	rawn	1000	L1	0	0	4	0	0	0
Wang	rawn	1000	L2	0	0	3	0	0	0
Wang	rawn	1000	Pe	0	0	0	0	0	0
Wang	rawn	2000	L1	0	0	2	0	0	2
Wang	rawn	2000	L2	0	0	2	0	0	0
Wang	rawn	2000	Pe	0	0	0	0	0	0
Wang	rawn	3000	L1	0	0	1	0	0	0
Wang	rawn	3000	L2	0	0	6	0	0	0
Wang	rawn	3000	Pe	0	0	0	0	0	0
Wang	rawn	4000	L1	0	0	1	0	0	1
Wang	rawn	4000	L2	0	0	6	0	0	0
Wang	rawn	4000	Pe	0	0	0	0	0	0
Wang	rawn	5000	L1	0	0	1	0	0	1
Wang	rawn	5000	L2	0	0	5	0	0	0
Wang	rawn	5000	Pe	0	0	0	0	0	0
Wang	rawn	20000	L1	0	0	2	0	0	2
Wang	rawn	20000	L2	0	0	1	0	0	0
Wang	rawn	20000	Pe	0	0	0	0	0	0

```
save(df, file = paste0(
  paste0(dirAbundanceMatrices, "ABM_NRAW"),
  "_results_phase2.rda"
))
```

```
df = postDA(
  hd = paste0(dirAbundanceMatrices, "ABM_NLOG/"),
  model_matrix = model_matrix,
  alpha = 0.05,
  coefficient = 2:3
)
```

```
df$res1  # Table 7
```

Table 7: The results correspond to model with predictor ‘phase‘. Number of significant clusters using the likelihood approach (sig.glm) and quasi-likelihood (sig.ql) for database of study (db), ‘log‘ normalization method (normalization), number of HVG (ngenes) and metric/dissimilarity (diss).

db	normalization	ngenes	diss	sig.glm	sig.ql
Wang	lg1n	100	L1	28	14
Wang	lg1n	100	L2	28	17
Wang	lg1n	100	Pe	29	12
Wang	lg1n	500	L1	29	13
Wang	lg1n	500	L2	27	14
Wang	lg1n	500	Pe	29	12
Wang	lg1n	1000	L1	29	18
Wang	lg1n	1000	L2	26	10
Wang	lg1n	1000	Pe	28	4
Wang	lg1n	2000	L1	30	16
Wang	lg1n	2000	L2	29	21
Wang	lg1n	2000	Pe	25	1
Wang	lg1n	3000	L1	30	17
Wang	lg1n	3000	L2	29	26
Wang	lg1n	3000	Pe	27	9
Wang	lg1n	4000	L1	30	18
Wang	lg1n	4000	L2	30	26
Wang	lg1n	4000	Pe	29	24
Wang	lg1n	5000	L1	30	18
Wang	lg1n	5000	L2	30	22
Wang	lg1n	5000	Pe	28	23
Wang	lg1n	20000	L1	30	28
Wang	lg1n	20000	L2	29	19
Wang	lg1n	20000	Pe	30	24

```
save(df,
     file = paste0(dirAbundanceMatrices, "ABM_NLOG",
                   "_results_phase1.rda"))
```

```
df = postDA(
  hd = paste0(dirAbundanceMatrices, "ABM_NLOG/"),
  model_matrix = model_matrix,
  alpha = 0.05,
  contrast = contrast
)
```

```
df$res2  # Table 8
```

Table 8: The results correspond to model with predictor 'phase' and tests the different contrasts comparing each pair of phases. Number of significant clusters using the likelihood approach (sig.glm1-3) and quasi-likelihood (sig.ql1-3) for database of study (db), 'log' normalization method (normalization), number of HVG (ngenes) and metric/dissimilarity (diss).

db	normalization	ngenes	diss	sig.glm1	sig.glm2	sig.glm3	sig.ql1	sig.ql2	sig.ql3
Wang	lg1n	100	L1	0	0	0	0	0	0
Wang	lg1n	100	L2	0	0	0	0	0	0
Wang	lg1n	100	Pe	0	0	0	0	0	0
Wang	lg1n	500	L1	0	0	0	0	0	0
Wang	lg1n	500	L2	0	0	0	0	0	0
Wang	lg1n	500	Pe	0	0	0	0	0	0
Wang	lg1n	1000	L1	0	0	2	0	0	0
Wang	lg1n	1000	L2	0	0	0	0	0	0
Wang	lg1n	1000	Pe	0	0	0	0	0	0
Wang	lg1n	2000	L1	0	0	0	0	0	0
Wang	lg1n	2000	L2	0	0	0	0	0	0
Wang	lg1n	2000	Pe	0	0	0	0	0	0
Wang	lg1n	3000	L1	0	0	0	0	0	0
Wang	lg1n	3000	L2	0	0	0	0	0	0
Wang	lg1n	3000	Pe	0	0	0	0	0	0
Wang	lg1n	4000	L1	0	0	0	0	0	0
Wang	lg1n	4000	L2	0	0	0	0	0	0
Wang	lg1n	4000	Pe	0	0	0	0	0	0
Wang	lg1n	5000	L1	0	0	0	0	0	0
Wang	lg1n	5000	L2	0	0	0	0	0	0
Wang	lg1n	5000	Pe	0	0	0	0	0	0
Wang	lg1n	20000	L1	0	0	0	0	0	0
Wang	lg1n	20000	L2	0	0	0	0	0	0
Wang	lg1n	20000	Pe	0	0	0	0	0	0

```
save(df, file = paste0(
  paste0(dirAbundanceMatrices, "ABM_NLOG"),
  "_results_phase2.rda"
))
```

Differential abundance with filtering

This section contains the differential abundance analyses with a filtered set of cells. In particular, cells with a low silhouette are removed from the analysis.

`time`

Differential abundance analyses with `time` predictor for `raw` normalization:

```
model_matrix = model.matrix( ~ time)
df = postDA(
  hd = paste0(dirAbundanceMatrices, "ABM_NRAW_FILT_SIL/"),
  model_matrix = model_matrix,
  alpha = 0.05,
  coefficient = 2,
  filtering = TRUE
)
```

```
df$res1  # Table 9
## df$sig1
```

Table 9: The results correspond to model with predictor ‘time‘ with filtered set. Number of significant clusters using the likelihood approach (sig.glm) and quasi-likelihood (sig.ql) for database of study (db), ‘raw‘ normalization method (normalization), number of HVG (ngenes) and metric/dissimilarity (diss).

db	normalization	ngenes	diss	sig.glm	sig.ql
Wang	rawn	100	L2	4	5
Wang	rawn	500	L2	4	0
Wang	rawn	1000	L2	2	0
Wang	rawn	2000	L2	3	3
Wang	rawn	3000	L2	2	0
Wang	rawn	4000	L2	2	0
Wang	rawn	5000	L2	2	1
Wang	rawn	20000	L2	0	0

```
save(df, file = paste0(
  paste0(dirAbundanceMatrices, "ABM_NRAW_FILT_SIL"),
  "_results_time.rda"
))
```

time2

Differential abundance analyses with `time` predictor for `raw` normalization:

```
model_matrix = model.matrix( ~ time2)
df = postDA(
  hd = paste0(dirAbundanceMatrices, "ABM_NRAW_FILT_SIL/"),
  model_matrix = model_matrix,
  alpha = 0.05,
  coefficient = 2,
  filtering = TRUE
)
```

```
df$res1  # Table 10
## df$sig1
```

Table 10: The results correspond to model with predictor 'time2' with filtered set. Number of significant clusters using the likelihood approach (sig.glm) and quasi-likelihood (sig.ql) for database of study (db), 'raw' normalization method (normalization), number of HVG (ngenes) and metric/dissimilarity (diss).

db	normalization	ngenes	diss	sig.glm	sig.ql
Wang	rawn	100	L2	10	0
Wang	rawn	500	L2	8	1
Wang	rawn	1000	L2	7	0
Wang	rawn	2000	L2	6	0
Wang	rawn	3000	L2	2	0
Wang	rawn	4000	L2	6	0
Wang	rawn	5000	L2	5	0
Wang	rawn	20000	L2	1	0

```
save(df,
     file = paste0(
       dirAbundanceMatrices,
       "ABM_NRAW_FILT_SIL",
       "_results_time2.rda"
```

```
      ))
```

`phase`

Differential abundance analyses with `phase` predictor for `raw` normalization:

```
model_matrix = model.matrix( ~ phase)
df = postDA(
  hd = paste0(dirAbundanceMatrices, "ABM_NRAW_FILT_SIL/"),
  model_matrix = model_matrix,
  alpha = 0.05,
  coefficient = 2:3,
  filtering = TRUE
)
```

```
df$res1  # Table 11
## df$sig1
```

Table 11: The results correspond to model with predictor 'phase' with filtered set. Number of significant clusters using the likelihood approach (sig.glm) and quasi-likelihood (sig.ql) for database of study (db), 'raw' normalization method (normalization), number of HVG (ngenes) and metric/dissimilarity (diss).

db	normalization	ngenes	diss	sig.glm	sig.ql
Wang	rawn	100	L2	5	0
Wang	rawn	500	L2	3	0
Wang	rawn	1000	L2	2	0
Wang	rawn	2000	L2	7	2
Wang	rawn	3000	L2	8	1
Wang	rawn	4000	L2	6	1
Wang	rawn	5000	L2	6	1
Wang	rawn	20000	L2	3	0

```
save(df,
     file = paste0(
       dirAbundanceMatrices,
       "ABM_NRAW_FILT_SIL",
       "_results_phase1.rda"
     ))
```

```
model_matrix = model.matrix( ~ 0 + phase)
contrast = makeContrasts(
  c1 = phase3 - phase4,
  c2 = phase3 - phase5,
  c3 = phase4 - phase5,
  levels = model_matrix
)
df = postDA(
  hd = paste0(dirAbundanceMatrices, "ABM_NRAW_FILT_SIL/"),
  model_matrix = model_matrix,
  alpha = 0.05,
  contrast = contrast
)
```

```
df$res2  # Table 12
```

Table 12: The results correspond to model with predictor 'phase' with filtered set and tests the different contrasts comparing each pair of phases. Number of significant clusters using the likelihood approach (sig.glm1-3) and quasi-likelihood (sig.ql1-3) for database of study (db), 'raw' normalization method (normalization), number of HVG (ngenes) and metric/dissimilarity (diss).

db	normalization	ngenes	diss	sig.glm1	sig.glm2	sig.glm3	sig.ql1	sig.ql2	sig.ql3
Wang	rawn	100	L2	0	0	5	0	0	0
Wang	rawn	500	L2	0	0	2	0	0	0
Wang	rawn	1000	L2	0	0	3	0	0	0
Wang	rawn	2000	L2	0	0	2	0	0	0
Wang	rawn	3000	L2	0	0	6	0	0	0
Wang	rawn	4000	L2	0	0	6	0	0	0
Wang	rawn	5000	L2	0	0	5	0	0	0
Wang	rawn	20000	L2	0	0	1	0	0	0

```
save(df,
     file = paste0(
       dirAbundanceMatrices,
       "ABM_NRAW_FILT_SIL",
       "_results_phase.rda"
     ))
```

Plots

The following plot shows the number of significant clusters using the raw normalization for different number of previously chosen genes.

First, we analyze `time2`.

```
load(paste0(dirAbundanceMatrices, "ABM_NRAW", "_results_time2.rda"))
df1 = matrix(NA, nrow = 24, ncol = 7)
df1 = as.data.frame(df1)
df1[1:24, 1:6] = df$res1
df1[1:24, 7] = rep("time2", 24)
names(df1) = c(names(df$res1), "predictor")
View(df1)
df1$diss = factor(df1$diss)
df1$predictor = factor(df1$predictor)
p = ggplot(df1, aes(x = ngenes, y = sig.glm, color = diss)) + geom_point()
p = p + facet_grid(. ~ predictor)
p = p + xlab("Number of genes") + ylab("Number of significant clusters") +
  theme(
    panel.grid.major = element_blank(),
    panel.grid.minor = element_blank(),
    panel.background = element_blank(),
    axis.line = element_line(colour = "black")
  )
ggsave("SigClust_nraw_time2.png", p)
```

```
Saving 5.5 x 3.5 in image
```

Now, we compare the results with `time` and `time2`.

```
load(paste0(dirAbundanceMatrices, "ABM_NRAW", "_results_time.rda"))
df1 = matrix(NA, nrow = 48, ncol = 7)
df1 = as.data.frame(df1)
df1[1:24, 1:6] = df$res1
df1[1:24, 7] = rep("time", 24)

load(paste0(dirAbundanceMatrices, "ABM_NRAW", "_results_time2.rda"))
df1[25:48, 1:6] = df$res1
df1[25:48, 7] = rep("time2", 24)
names(df1) = c(names(df$res1), "predictor")
View(df1)
```

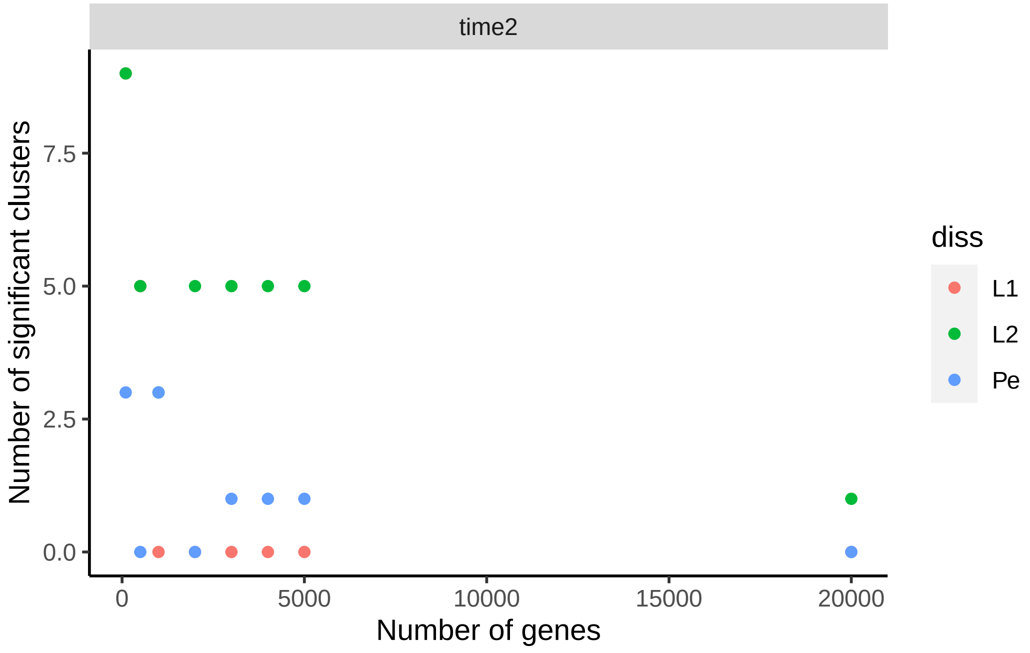

Figure 1: Number of significant clusters using the `raw` normalization with `time2` predictor.

```
df1$diss = factor(df1$diss)
df1$predictor = factor(df1$predictor)
p = ggplot(df1, aes(x = ngenes, y = sig.glm, color = diss)) + geom_point()
p = p + facet_grid(. ~ predictor)
p = p + xlab("Number of genes") + ylab("Number of significant clusters") +
  theme(
    panel.grid.major = element_blank(),
    panel.grid.minor = element_blank(),
    panel.background = element_blank(),
    axis.line = element_line(colour = "black")
  )
ggsave("SigClust_nraw_time.png", p)
```

```
Saving 5.5 x 3.5 in image
```

The following plot shows the number of significant clusters using filtered cell sets where the predictors `time` and `time2` are compared for the Euclidean metric.

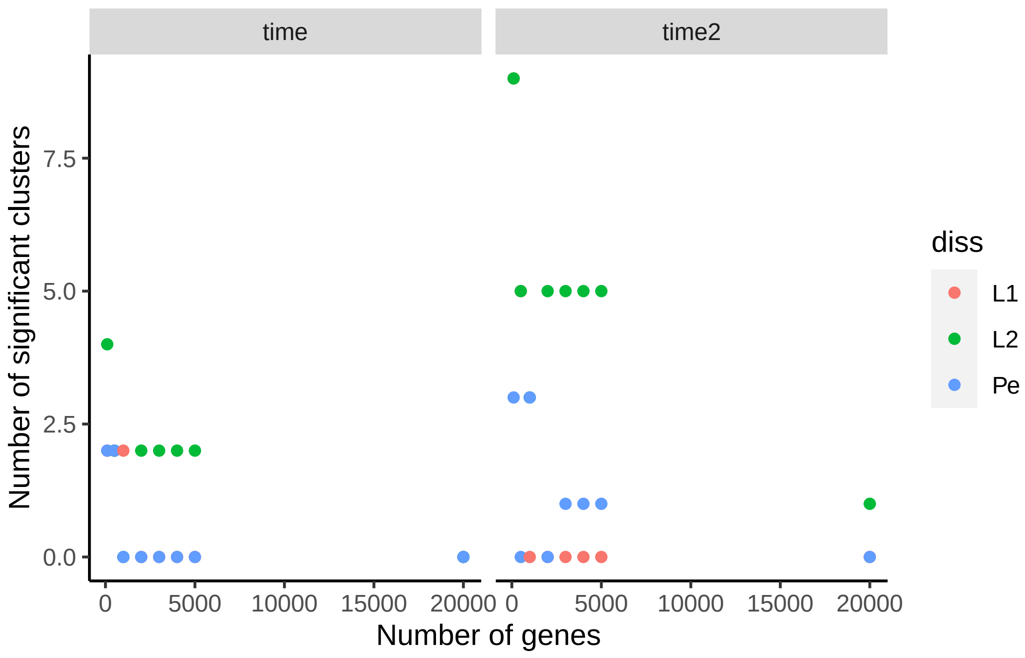

Figure 2: Number of significant clusters using the `raw` normalization comparing the results with `time` and `time2` predictors.

```
load(paste0(
  dirAbundanceMatrices,
  "ABM_NRAW_FILT_SIL",
  "_results_time2.rda"
))
df1 = matrix(NA, nrow = 8, ncol = 7)
df1 = as.data.frame(df1)
df1[1:8, 1:6] = df$res1
df1[1:8, 7] = rep("time2", 8)

names(df1) = c(names(df$res1), "type")
View(df1)

df1$type = factor(df1$type)
p = ggplot(df1, aes(x = ngenes, y = sig.glm, color = type)) + geom_point()
p = p + xlab("Number of genes") + ylab("Number of significant clusters") +
  theme(
    panel.grid.major = element_blank(),
    panel.grid.minor = element_blank(),
```

```
    panel.background = element_blank(),
    axis.line = element_line(colour = "black")
  )
ggsave("SigClust_nraw_time_filtering.png", p)
```

```
Saving 5.5 x 3.5 in image
```

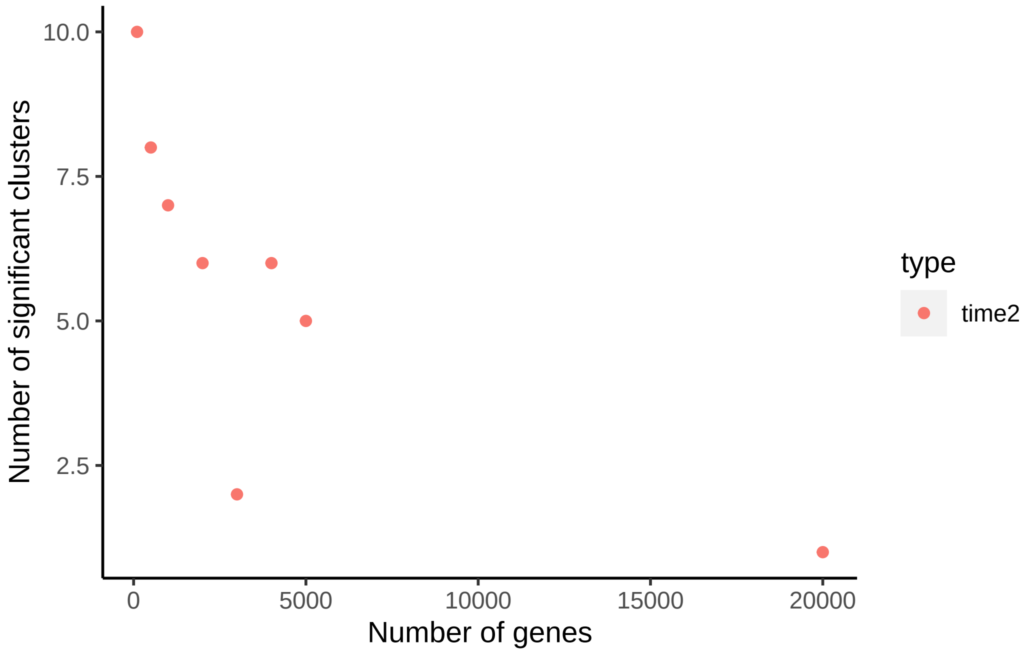

Figure 3: Number of significant clusters using filtered cell sets where the predictors `time` and `time2` are compared for the Euclidean metric.

Now we compare the results with and without filtering.

```
load(paste0(dirAbundanceMatrices, "ABM_NRAW", "_results_time2.rda"))
df1 = matrix(NA, nrow = 16, ncol = 8)
df1 = as.data.frame(df1)
df1[1:8, 1:6] = df$res1[df$res1$diss == "L2", ]
df1[1:8, 7] = rep("time2", 8)
df1[1:8, 8] = rep("without", 8)

load(paste0(
  dirAbundanceMatrices,
```

```
    "ABM_NRAW_FILT_SIL",
    "_results_time2.rda"
  ))
  df1[9:16, 1:6] = df$res1
  df1[9:16, 7] = rep("time2", 8)
  df1[9:16, 8] = rep("with", 8)

  names(df1) = c(names(df$res1), "type", "filtering")

  df1$type = factor(df1$type)
  df1$filtering = factor(df1$filtering)

  p = ggplot(df1, aes(x = ngenes, y = sig.glm, color = filtering)) + geom_point()
  p = p + facet_grid(. ~ type)
  p = p + xlab("Number of genes") + ylab("Number of significant clusters") +
    theme(
      panel.grid.major = element_blank(),
      panel.grid.minor = element_blank(),
      panel.background = element_blank(),
      axis.line = element_line(colour = "black")
    )
  ggsave("SigClust_nraw_L2_filtering_time2.png", p)
```

```
Saving 5.5 x 3.5 in image
```

```
  load(paste0(dirAbundanceMatrices, "ABM_NRAW", "_results_time.rda"))
  df1 = matrix(NA, nrow = 32, ncol = 8)
  df1 = as.data.frame(df1)
  df1[1:8, 1:6] = df$res1[df$res1$diss == "L2", ]
  df1[1:8, 7] = rep("time", 8)
  df1[1:8, 8] = rep("without", 8)

  load(paste0(dirAbundanceMatrices, "ABM_NRAW", "_results_time2.rda"))
  df1[9:16, 1:6] = df$res1[df$res1$diss == "L2", ]
  df1[9:16, 7] = rep("time2", 8)
  df1[9:16, 8] = rep("without", 8)

  load(paste0(
    dirAbundanceMatrices,
    "ABM_NRAW_FILT_SIL",
    "_results_time.rda"
```

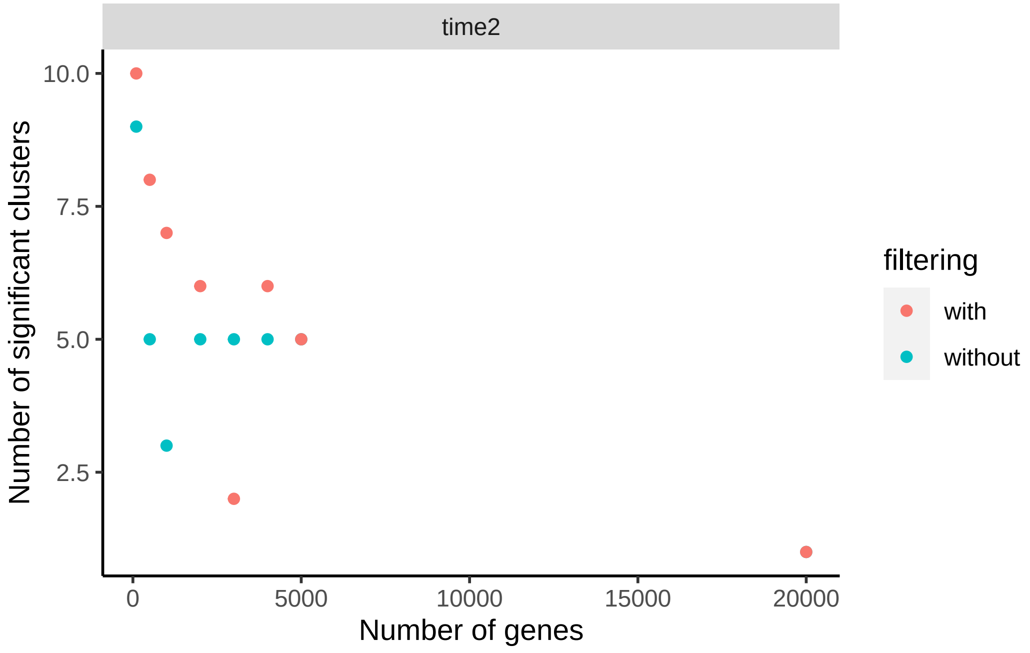

Figure 4: Number of significant clusters using the predictor `time2`, where the results with and without filtering cell sets are compared for the Euclidean metric.

```
))
df1[17:24, 1:6] = df$res1
df1[17:24, 7] = rep("time", 8)
df1[17:24, 8] = rep("with", 8)

load(paste0(
  dirAbundanceMatrices,
  "ABM_NRAW_FILT_SIL",
  "_results_time2.rda"
))
df1[25:32, 1:6] = df$res1
df1[25:32, 7] = rep("time2", 8)
df1[25:32, 8] = rep("with", 8)

names(df1) = c(names(df$res1), "type", "filtering")

df1$type = factor(df1$type)
df1$filtering = factor(df1$filtering)
```

```
p = ggplot(df1, aes(x = ngenes, y = sig.glm, color = filtering)) + geom_point()
p = p + facet_grid(. ~ type)
p = p + xlab("Number of genes") + ylab("Number of significant clusters") +
  theme(
    panel.grid.major = element_blank(),
    panel.grid.minor = element_blank(),
    panel.background = element_blank(),
    axis.line = element_line(colour = "black")
  )
ggsave("SigClust_nraw_L2_filtering.png", p)
```

```
Saving 5.5 x 3.5 in image
```

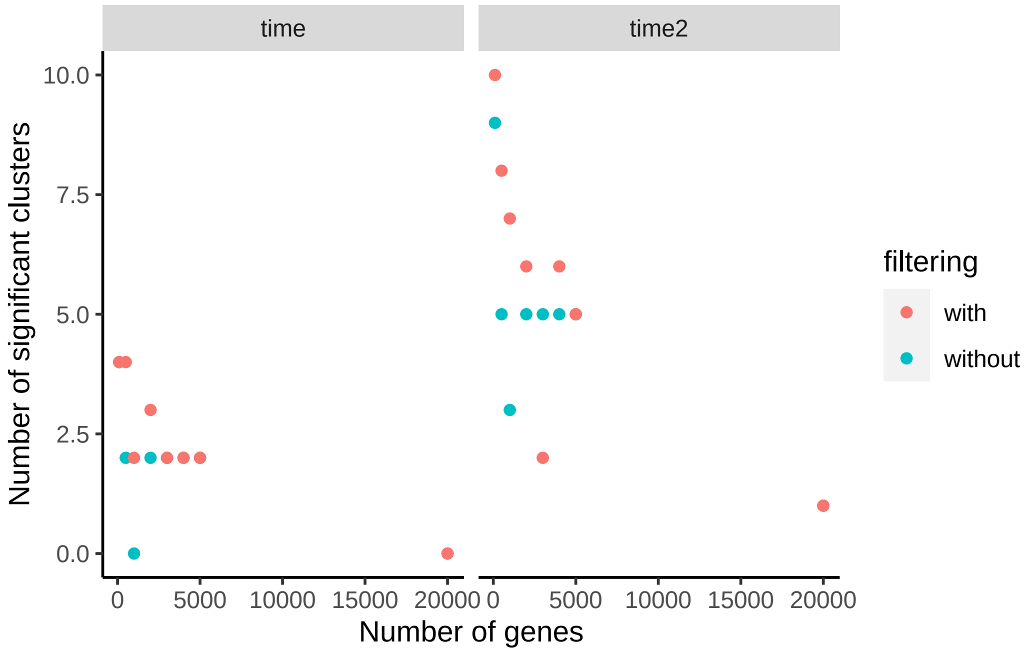

Figure 5: Number of significant clusters using filtered (with) and unfiltered (without) cell sets where the predictors `time` and `time2` are compared for the Euclidean metric.

Multidimensional scaling over the medoids

```
finput1 = "../../../../BinaryData/COUNT_MATRICES/Wang_lg1n_f0100.bin"
finput2 = "../../../../RESULTS_PAM_CLUSTERING/Wang_lg1n_f0100_Pe.rda"
load(finput2)
load("Supl/c.10x_metadata.RData")
CoordMed = GetJManyRows(finput1, L$med)
med.clas = L$clasif[which(is.element(names(L$clasif), rownames(CoordMed)))]
xy = cmdscale(dist(CoordMed))
xy = data.frame(xy, med.clas)
names(xy) = c("x", "y", "class")
type = metadata[match(rownames(xy), rownames(metadata)), "cell_type"]
xy = data.frame(xy, type)
p = ggplot(xy, aes(x = x, y = y, color = type)) + geom_point() +
  theme(panel.background = element_rect(fill = "white"),
        axis.line = element_line(colour = "black")) +
  scale_color_manual(
    values = c(
      "#8DD3C7",
      "#FFFFB3",
      "#BEBADA",
      "#80B1D3",
      "#FDB462",
      "#B3DE69",
      "#FCCDE5",
      "#D9D9D9"
    )
  )
ggsave("Wang_lg1n_f0100_mds.png", p)
```


Lund, Steven P., Dan Nettleton, Davis J. McCarthy, and Gordon K. Smyth. 2012. "Detecting Differential Expression in RNA-Sequence Data Using Quasi-Likelihood with Shrunken Dispersion Estimates." *Statistical Applications in Genetics and Molecular Biology* 11 (5). https://doi.org/doi:10.1515/1544-6115.1826.

Maechler, Martin, Peter Rousseeuw, Anja Struyf, and Mia Hubert. 2022. *Cluster: "Finding Groups in Data": Cluster Analysis Extended Rousseeuw Et Al.* https://svn.r-project.org/R-packages/trunk/cluster/.

McCarthy, Davis J., Yunshun Chen, and Gordon K. Smyth. 2012. "Differential Expression Analysis of Multifactor RNA-Seq Experiments with Respect to Biological Variation." *Nucleic Acids Research* 40 (10): 4288–97. https://doi.org/10.1093/nar/gks042.

Wickham, Hadley, Winston Chang, Lionel Henry, Thomas Lin Pedersen, Kohske Takahashi, Claus Wilke, Kara Woo, Hiroaki Yutani, and Dewey Dunnington. 2022. *Ggplot2: Create*

Elegant Data Visualisations Using the Grammar of Graphics. https://CRAN.R-project.org/package=ggplot2.
